# Divergent roles of SPOP and CHD1 in ACSL4 regulation reveal context-dependent vulnerabilities for targeting ferroptosis

**DOI:** 10.1038/s41467-026-75010-y

**Published:** 2026-06-30

**Authors:** Feiyu Chen, Qidong Li, Qianlin Gu, Javier Leo, Xin Liang, Naayaa Mehta, Estefania Labanca, Peter Shepherd, Iqbal Mahmud, Yin Wang, Francisco R. Saenz, Maya M. Phillips, Wei Shi, Chenling Meng, Jie Zhang, Daniel E. Frigo, Yue Lu, Boyi Gan, Di Zhao

**Affiliations:** 1 Department of Experimental Radiation Oncology, The University of Texas MD Anderson Cancer Center, Houston, TX USA; 2 UTHealth Graduate School of Biomedical Sciences, The University of Texas MD Anderson Cancer Center, Houston, TX USA; 3 Department of Genitourinary Medical Oncology, Division of Cancer Medicine, The University of Texas MD Anderson Cancer Center, Houston, TX USA; 4 Metabolomics Core Facility, Department of Bioinformatics and Computational Biology, The University of Texas MD Anderson Cancer Center, Houston, TX USA; 5 Department of Radiation Oncology, The University of Texas MD Anderson Cancer Center, Houston, TX USA; 6 Department of Cancer Systems Imaging, The University of Texas MD Anderson Cancer Center, Houston, TX USA; 7 Department of Epigenetics and Molecular Carcinogenesis, The University of Texas MD Anderson Cancer Center, Houston, TX USA

**Keywords:** Prostate cancer, Cell death, Cancer genetics

## Abstract

Genetic heterogeneity contributes to the variable therapeutic responses in cancers. Frequent *SPOP* mutations and recurrent *CHD1* deletions define distinct molecular subtypes of prostate cancer (PCa) with differential responses to anti-androgen therapy. Ferroptosis, an iron-dependent cell death mechanism driven by lipid peroxidation, has emerged as a promising anticancer strategy. Here, we identify *SPOP* mutations and *CHD1* deletion as key genetic determinants of ferroptosis susceptibility in PCa. Using genetically engineered human and murine models, we show that *SPOP* mutations enhance, whereas *CHD1* deletion impairs, the efficacy of ferroptosis inducers targeting GPX4. Mechanistically, SPOP and CHD1 exert opposing effects on ferroptosis by antagonistically regulating the MYC–ACSL4 axis. Furthermore, we demonstrate that targeting cholesterol metabolism with cholesterol-lowering agents restores ACSL4 expression and re-sensitizes *SPOP/CHD1* co-deficient tumors to ferroptosis-inducing therapy. Our findings establish SPOP/CHD1 as upstream genetic regulators of ferroptosis and provide biomarker-driven combinatorial strategies to enhance ferroptosis-based therapy in men with advanced PCa.

## Abstract (web summary)

SPOP mutations and CHD1 deletion frequently co-occur in prostate cancer. Here the authors report that SPOP mutations enhance, whereas CHD1 deletion impairs, the efficacy of ferroptosis inducers in human and mouse prostate cancer models.

## Introduction

Prostate cancer (PCa) is the most commonly diagnosed malignancy in men in the United States^1^. PCa exhibits substantial interpatient genetic heterogeneity, as evidenced by genomic studies that identify a spectrum of recurrent somatic alterations in both primary and advanced disease^2–4^. This genetic diversity presents opportunities for discovering context-dependent therapeutic vulnerabilities, enabling precision oncology interventions. While some genetic events have been incorporated into the molecular subclassification of PCa and have exhibited prognostic values^5,6^, only a limited number have been functionally characterized or translated into effective therapies. It highlights the crucial need to define actionable genetic alterations in PCa and to uncover therapeutic vulnerabilities in each molecular subtype.

Nearly 15% of primary and metastatic PCa contain loss-of-function *SPOP* (Speckle-type BTB/POZ protein) mutations, which define a distinct molecular subtype^5,7–9^. As a substrate adaptor of the Cullin3-RING ubiquitin E3 ligase, SPOP contributes to proteasomal degradation of various oncogenic proteins, including MYC^10^, AR^11,12^, and AR co-activators such as ERG^13,14^, TRIM24^15^, and SRC-3^16^. Recurrent mutations in the substrate-binding MATH domain of SPOP (e.g., Y87C, F102C, F125L, W131G, F133V) impair substrate recognition, thereby stabilizing those oncogenic proteins and activating signaling pathways that promote tumorigenesis^17,18^. Several clinical studies reported that PCa patients with *SPOP* mutations exhibit improved survival following first-line androgen deprivation therapy (ADT)^19–21^ and better response to AR axis-targeted therapy^22,23^. These preclinical and clinical studies demonstrate the prognostic value of *SPOP* mutations in treating PCa.

As one of the most frequent co-occurring genetic events, loss of *CHD1* (Chromodomain helicase DNA-binding protein 1) exists in nearly half of *SPOP*-mutated PCa^3,4,24^. CHD1 is a chromatin remodeler involved in nucleosome positioning and transcriptional regulation^25–27^. Emerging studies, including ours, have demonstrated that *CHD1* loss coordinates with oncogenic pathways in a context-dependent manner and contributes to therapeutic resistance^28–36^. Recently, we reported that *CHD1* deletion promotes prostate tumorigenesis and confers castration resistance in SPOP-mutated PCa by reprogramming cholesterol metabolism^36^. These findings underscore the distinct effects of *SPOP* mutations and *CHD1* deletion on therapy response, laying the foundation for using them as genetic biomarkers for personalized medicine in men with advanced PCa.

Ferroptosis is a regulated form of cell death triggered by excessive iron-dependent lipid peroxidation in the cellular membrane^37,38^. Accordingly, a key step in ferroptosis is the regulation of the levels of lipid substrates that can be readily oxidized. The enzyme ACSL4 (acyl-CoA synthetase long-chain family member 4) ligates free PUFAs (polyunsaturated fatty acids) with CoA to produce PUFA-CoA, a critical precursor for synthesizing PUFA-PLs (PUFA-containing phospholipids), which are particularly susceptible to lipid peroxidation. Thus, ACSL4 levels serve as a predictor for ferroptosis sensitivity^39^. Conversely, the cystine-GSH-GPX4 regulatory axis serves as a canonical anti-ferroptosis mechanism by neutralizing PLOOHs (phospholipid hydroperoxides)^38^. Ferroptosis inducers targeting cystine/glutamate transporter (erastin) or GPX4 (RSL3, ML162, and JKE1674) have garnered considerable attention due to their anti-cancer potential. Increasing evidence suggests that inducing ferroptosis enhances therapeutic benefits, particularly in cancers with specific molecular features^40–44^. However, genetic events that influence PCa’s response to ferroptosis-targeted therapy remain understudied.

In this study, we identified *SPOP* mutations and *CHD1* deletion as genetic determinants of ferroptosis susceptibility in PCa. Using CRISPR-Cas9-engineered isogeneic cell lines, genetically engineered mouse models (GEMMs), xenograft models, and 3D organoid systems, we uncover the distinct effects of *SPOP* and *CHD1* on ferroptosis sensitivity and present their opposite roles in regulating ACSL4 expression and lipid peroxidation. Furthermore, we develop effective combinatorial strategies to enhance ferroptosis-targeted therapy in advanced PCa containing *SPOP/CHD1* co-defects.

## Results

### SPOP mutations sensitize PCa cells to ferroptosis inducers targeting GPX4

**Fig. 1:**
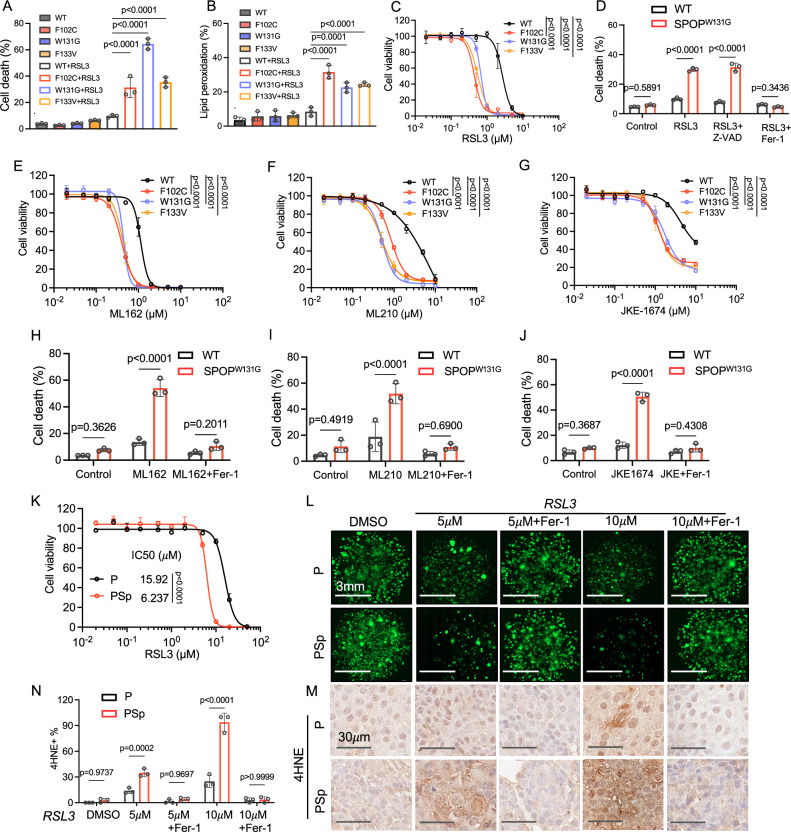
SPOP mutations sensitize PCa cells to ferroptosis inducers targeting GPX4. **A–C** Quantifications of cell death (**A**), lipid peroxidation (**B**), or dose-response (**C**) in wildtype (WT) and SPOP-mutated (F102C, W131G, F133V) LNCaP cells treated with or without RSL3 (1 μM, 24 h). **D** Cell death in WT and SPOP^W131G^ LNCaP cells treated with RSL3 (1 μM, 24 h) in the presence of Z-VAD-FMK (Z-VAD, 10 μM, 24 h) or ferrostatin-1 (Fer-1, 10 μM, 24 h). **E–G** Dose-response curves of ML162 (**E**), ML210 (**F**), or JKE-1674 (**G**) in WT versus SPOP-mutated LNCaP cells. **H–J** Cell death in WT and SPOP^W131G^ LNCaP cells treated with or without 1 μM ML162 (**H**), 1 μM ML210 (**I**), or 10 μM JKE-1674 (**J**) for 24 h, with or without 10 μM Fer-1. **K** Tumor organoids derived from *Pten*^*L/L*^ (P) and *Pten*^*L/L*^*; Spop*^*F133V*^ (PSp) GEMMs were treated with series doses of RSL3 for 6-day, followed by CellTiter-Glo luminescent cell viability assays. Dose-response curves and IC50s are shown. **L** Representative images of GFP + P and PSp organoids after 6-day treatment of RSL3, with or without 10 μM Fer-1. Each GFP+ dot indicates an organoid. Scale bar, 3 mm. **M**, **N** IHC staining (**M**) and quantification of 4HNE (**N**) in P and PSp organoids after treatment. Scale bar, 30 μm. Data are presented as mean ± standard deviation, *n* = 3 biologically independent repeats. *P* values determined by one-way (**A**, **B**) or two-way (**C**–**K**, **N**) ANOVA analysis. Source data are provided as a Source Data file.

Given that genetic alterations of *SPOP* in PCa are often heterozygous missense mutations and present dominant-negative loss of function toward the remaining wild-type allele^45^, we introduced V5-tagged *SPOP* mutants F102C, W131G, and F133V into LNCaP, a *SPOP* wild-type human PCa cell line, to mimic those genetic events (Fig. S1A). To determine the effects of *SPOP* mutations on ferroptosis sensitivity, we assessed the response of those LNCaP cells to the GPX4 inhibitor, RSL3. The results showed that expression of SPOP mutants markedly enhanced the sensitivity of PCa cells to ferroptosis, characterized by significantly increased cell death (Fig. 1A), elevated lipid peroxidation (Fig. 1B), and higher dose-response rates (Fig. 1C). To verify the type of cell death induced by RSL3 in *SPOP*-mutated LNCaP cells, we treated cells with the ferroptosis inhibitor ferrostatin-1 (Fer-1) or apoptosis inhibitor Z-VAD-FMK (Z-VAD). The results indicated that Fer-1, but not Z-VAD, can fully rescue cell viability after RSL3 treatment, confirming the ferroptotic nature of cell death (Figs. 1D and S1B). Similarly, *SPOP* mutations rendered LNCaP cells more susceptible to other ferroptosis inducers targeting GPX4, such as ML162, ML210, and JKE-1674 (Fig. 1E–G), which can be reversed by ferroptosis inhibitor treatment (Fig. 1H–J).

Given the importance of AR signaling in *SPOP*-mutated PCa, we further determined if androgen is required for the increased ferroptosis sensitivity driven by SPOP mutations. To this end, we cultured control and *SPOP*-mutated LNCaP cells with charcoal-stripped (CS) medium to mimic androgen deprivation conditions, and then determined their responses to GPX4 inhibitors RSL3, ML162, and JKE-1674. Phenocopying LNCaP cells cultured in androgen-containing medium, *SPOP* mutations effectively promoted LNCaP cells’ ferroptosis susceptibility under androgen deprivation conditions (Fig. S1C–E), suggesting these effects are independent of AR signaling.

Recently, we reported a series of GEMMs containing *CHD1* deletion and/or *SPOP* mutations to recapitulate different molecular subtypes of PCa, including *PB-Cre, Pten*^*L/L*^*, mTmG* (*referred to as* P), *PB-Cre, Pten*^*L/L*^*, Spop*^*F133V*^, *mTmG* (PSp), and *PB-Cre, Pten*^*L/L*^*, Spop*^*F133V*^, *Chd1*^*L/L*^*, mTmG* (PSpC)^36^. Additionally, we generated several 3D tumor organoid lines from these GEMMs, which facilitated the assessment of therapeutic response and mechanistic studies. To verify the effects of SPOP mutations on ferroptosis in a more physiologically relevant system, we determined the dose-response of P and PSp tumor organoids to RSL3. Compared to P organoids, PSp organoids exhibited greater sensitivity to RSL3, featured by a lower IC_50_ (15.92 vs. 6.24 μM) (Fig. 1K), regardless of the presence or absence of androgen in the culture medium (Fig. S1F). Ex vivo fluorescence imaging confirmed that PSp organoids were more susceptible to RSL3, while RSL3-induced cell death in PSp organoids was abolished entirely by ferroptosis inhibitor (Fig. 1L). Immunohistochemical (IHC) staining of 4-hydroxynonenal (4-HNE), a lipid peroxidation marker, revealed that RSL3 caused greater ferroptosis in PSp organoids, compared to P organoids (Fig. 1M, N). Collectively, these studies in human and mouse-derived models demonstrate that *SPOP* mutations sensitize PCa cells to ferroptosis inducers targeting GPX4, in an androgen-independent manner.

### ACSL4 mediates ferroptosis sensitivity driven by SPOP mutations

**Fig. 2:**
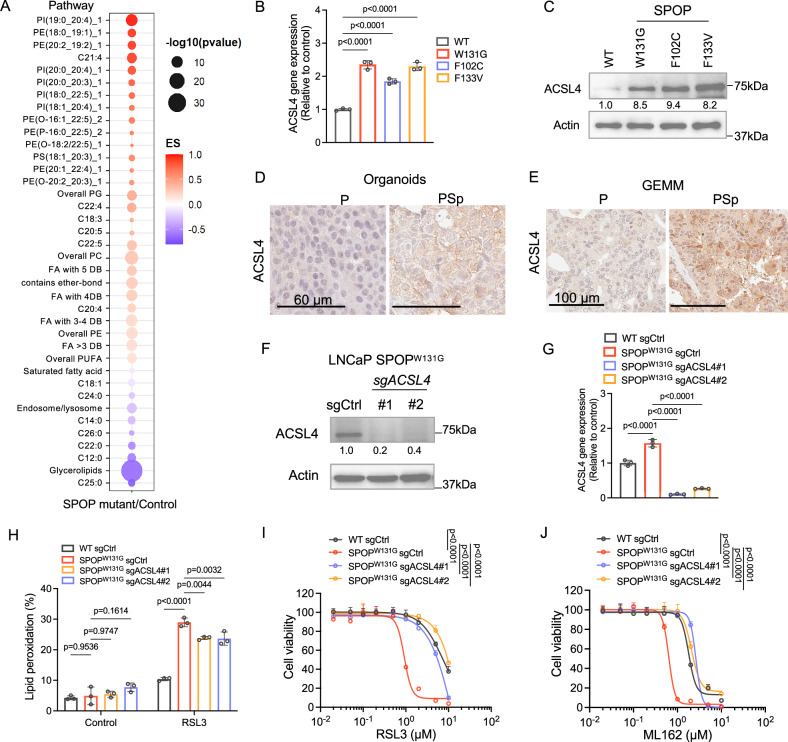
ACSL4 mediates ferroptosis sensitivity driven by SPOP mutations. **A** Lipidomic analysis reveals accumulation of PUFA-containing phospholipids in SPOP^W131G^ LNCaP cells, normalized to control cells; *n* = 4 biological replicates. Enrichment score (ES) and *p* values are shown. The significance of lipid ontology was assessed pairwise using Student’s *t*-test, followed by FDR correction. **B** ACSL4 mRNA levels in WT and SPOP-mutated (F102C, W131G, F133V) LNCaP cells. **C** Western blot analysis of ACSL4 protein in WT and SPOP-mutated LNCaP cells. The ACSL4/actin relative density ratio is shown. This was performed at least three times with similar results. **D**, **E** IHC staining of ACSL4 in organoids (**D**) and prostate tumors (**E**) derived from P and PSp GEMMs. Scale bars, 60 μm (**D**) and 100 μm (**E**). IHC staining was performed at least three times with similar results. **F**, **G** ACSL4 was genetically knocked out using two sgRNAs in SPOP^W131G^ LNCaP cells, followed by Western blot (**F**) and qPCR (**G**) analysis of ACSL4 expression. The ACSL4/actin relative density ratio is shown. **H** Lipid peroxidation in WT and SPOP^W131G^ LNCaP cells with or without ACSL4 depletion. **I**, **J** Dose-response curves of RSL3 (**I**) or ML162 (**J**) in WT and SPOP^W131G^ LNCaP cells with or without ACSL4 depletion. Data are presented as mean ± standard deviation, *n* = 3 biologically independent repeats unless otherwise stated. *P* values determined by one-way (**B**, **G**) or two-way (**H**–**J**) ANOVA analysis. Source data are provided as a Source Data file.

To elucidate the mechanism of ferroptosis sensitivity in *SPOP*-mutated cells, we performed lipidomic profiling in LNCaP cells expressing the W131G mutation (Fig. S2A, B). The results indicated that *SPOP* mutation induces widespread accumulation of polyunsaturated fatty acids (PUFAs) across multiple lipid classes and molecular species (Supplementary Data 1). The pathway enrichment analysis demonstrates significant elevation of various PUFA-containing phospholipids, with phosphatidylinositols (PI) and phosphatidylethanolamines (PE) showing the most pronounced enrichment, particularly PI (39:4) and PE (39:4) (Fig. 2A). This PUFA accumulation extends across different lipid categories, including phosphatidylserines (PS), and overall phosphoglycerides (PG), suggesting a systematic shift in cellular lipid composition (Fig. 2A). Notably, individual fatty acid species demonstrate robust enrichment, with C21:4, C18:3, and C22:5 showing significant upregulation in *SPOP*-mutated cells (Fig. 2A). The coordinated elevation of PUFAs with varying chain lengths (C12–C26) and desaturation levels (3–5 double bonds) indicates enhanced PUFA incorporation into membrane phospholipids rather than simple accumulation of free fatty acids (Fig. 2A). These findings suggest that *SPOP* mutations reprogram the metabolism of PUFA in PCa cells.

Given PUFA’s essential role in ferroptosis and the altered ferroptosis sensitivity in *SPOP*-mutated PCa cells, we next determined the effects of *SPOP* mutations on a panel of key ferroptosis regulators, including ACSL4, GPX4, SLC7A11 (Solute Carrier Family 7 Member 11), FSP1 (ferroptosis suppressor protein 1), DHODH (dihydroorotate dehydrogenase), NRF2 (nuclear factor erythroid 2-related factor 2), and GCH1 (GTP cyclohydrolase 1). The results indicated that *SPOP* mutations significantly increased ACSL4 expression at both the mRNA and protein levels (Fig. 2B, C), but had much less impact on other factors (Fig. S2C, D). Consistently, ACSL4 expression was also higher in organoids and prostate tumors from PSp GEMM than in P mice (Fig. 2D, E). These studies uncovered an underappreciated role of *SPOP* in regulating ACSL4, a master activator of ferroptosis. Of note, ACSL4 preferentially catalyzes the activation and incorporation of long-chain PUFAs, particularly arachidonic acid (C20:4) and adrenic acid (C22:4), into phospholipids. The convergence of *SPOP* mutation-driven ACSL4 upregulation and widespread PUFA-phospholipid enrichment (Fig. 2A and Supplementary Data 1) provides additional evidence for a mechanistic link in which *SPOP* defects enhance ACSL4-mediated PUFA esterification.

To verify if ACSL4 mediates the ferroptosis sensitivity driven by *SPOP* mutations, we knocked out ACSL4 using CRISPR-Cas9 in *SPOP*-mutated LNCaP cells (Fig. 2F, G). Upon RSL3 treatment, depletion of ACSL4 partially impaired the lipid peroxidation induced by *SPOP* mutations (Fig. 2H). Accordingly, dose-response assessments revealed that the loss of ACSL4 significantly reduced the sensitivity of *SPOP*-mutated cells to ferroptosis inducers, RSL3 and ML162 (Fig. 2I, J), suggesting that ACSL4 is required for ferroptosis susceptibility driven by *SPOP* defects.

### C-Myc stabilization links SPOP mutations to ACSL4-mediated ferroptosis

**Fig. 3:**
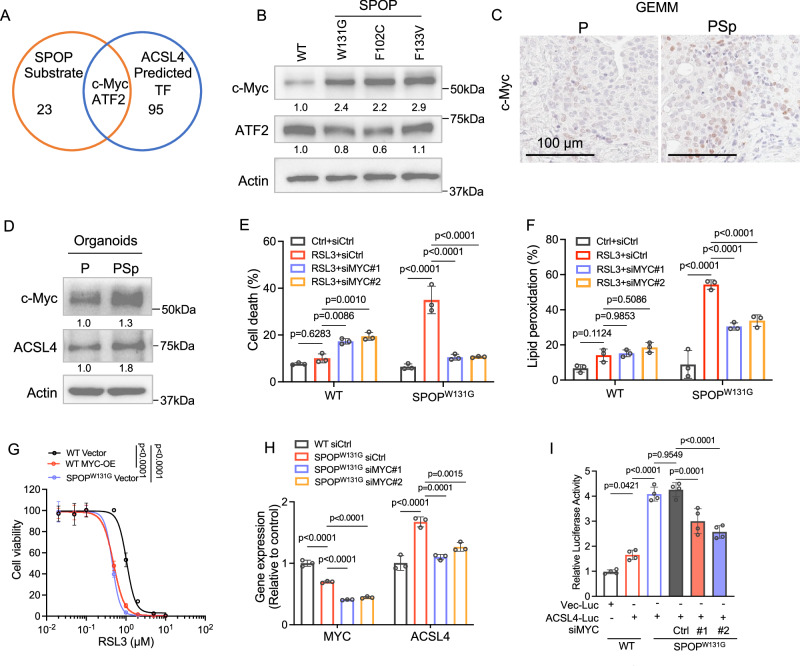
c-Myc stabilization links SPOP mutations to ACSL4-mediated ferroptosis. **A** Venn diagram presents the overlapped genes between known SPOP substrates (orange circle) and predicted transcriptional factors of ACSL4 (blue circle). **B** Western blot analysis of c-Myc and ATF2 protein levels in WT or SPOP-mutant LNCaP cells. Relative density ratios of indicated proteins/actin are shown. The samples derive from the same experiment, but different gels for c-Myc, ATF2, and another for Actin were processed in parallel. This was performed at least three times with similar results. **C** IHC staining of c-Myc in prostate tumors derived from P and PSp GEMMs. Scale bar, 100 μm. IHC staining was performed at least three times with similar results. **D** Western blot analysis of c-Myc and ACSL4 in P and PSp organoids. Relative density ratios of indicated proteins/actin are shown. The samples derive from the same experiment, but different gels for c-Myc, ACSL4, and another for Actin were processed in parallel. This was performed at least three times with similar results. **E**, **F** MYC was knocked down in WT and SPOP^W131G^ LNCaP cells using two individual siRNAs, followed by treatment with or without RSL3 (1 μM) for 24 h. Cell death (**E**) and lipid peroxidation (**F**) were determined. **G** MYC or SPOP^W131G^ was overexpressed in LNCaP cells, followed by determining the dose-response to RSL3 (24 h). **H** MYC was knocked down in WT and SPOP^W131G^ LNCaP cells using two individual siRNAs, followed by determining MYC and ACSL4 mRNA expression using qPCR. **I** A luciferase reporter of ACSL4 promoter was constructed and introduced into WT and SPOP^W131G^ LNCaP cells with or without MYC knockdown, followed by luciferase assays, *n* = 4 biological replicates. Data are presented as mean ± standard deviation, *n* = 3 biologically independent repeats unless otherwise stated. *P* values determined by one-way (**E**–**H**) or two-way (**I**) ANOVA analysis. Source data are provided as a Source Data file.

To understand how SPOP regulates ACSL4, we searched publicly available datasets and collected 97 predicted transcriptional factors that bind to the ACSL4 gene (Supplementary Data 2). By integrating them with 25 known SPOP substrate proteins, we identified two transcriptional factors, c-Myc and ATF2 (activating transcription factor 2), as potential mediators of SPOP-ACSL4 regulation (Fig. 3A). We first validated the protein expression of c-Myc and ATF2 in control versus *SPOP*-mutated LNCaP cells using Western blot analysis. The results showed that SPOP mutations caused the accumulation of c-Myc protein, but not ATF2 (Fig. 3B). Further, c-Myc protein levels were markedly elevated in prostate tumors and organoids derived from PSp GEMMs (Fig. 3C, D). MG132 treatment resulted in a time-dependent accumulation of c-Myc protein (Fig. S3A), confirming the degradation of c-Myc protein via the proteasome pathway. Protein stability assays also revealed that the half-life of c-Myc protein was prolonged in LNCaP cells expressing SPOP mutants (Fig. S3B, C). Together with prior studies^10^, these findings support SPOP as an E3 ligase of c-Myc protein and mediator of its proteasomal degradation.

To verify *MYC*’s role in ferroptosis of *SPOP*-mutated cells, we knocked down *MYC* using two individual siRNAs and then measured cell viability and lipid peroxidation following RSL3 treatment. As shown in Figs. 3E, F and S3D, MYC depletion slightly induced cell death in *SPOP*-wildtype cells but completely attenuated ferroptosis and lipid peroxidation in *SPOP*-mutated cells. In contrast, overexpression of c-Myc, which phenocopies *SPOP* mutations, rendered LNCaP cells more sensitive to the ferroptosis inducer RSL3 (Figs. 3G and S3E). These results indicated that the stabilized c-Myc protein contributes to ferroptosis sensitivity in *SPOP*-mutated cells.

Furthermore, we observed that knocking down *MYC* resulted in a marked reduction in ACSL4 expression in *SPOP*-mutated cells (Figs. 3H and S3D); whereas overexpression of c-Myc augmented ACSL4 mRNA expression (Fig. S3E). These findings are consistent with a recent report that c-Myc directly binds the ACSL4 promoter to activate its transcription^46^. Supporting this model, luciferase reporter assays revealed that *SPOP* mutations enhanced the transcription activity of the *ACSL4* promoter, which was significantly diminished upon *MYC* knockdown (Figs. 3I and S3F). Together, our studies revealed that *SPOP*-defect-driven stabilization of c-Myc activates ACSL4 transcription, thereby promoting ferroptosis sensitivity in PCa cells.

### Loss of CHD1 confers ferroptosis resistance in SPOP-mutated PCa

**Fig. 4:**
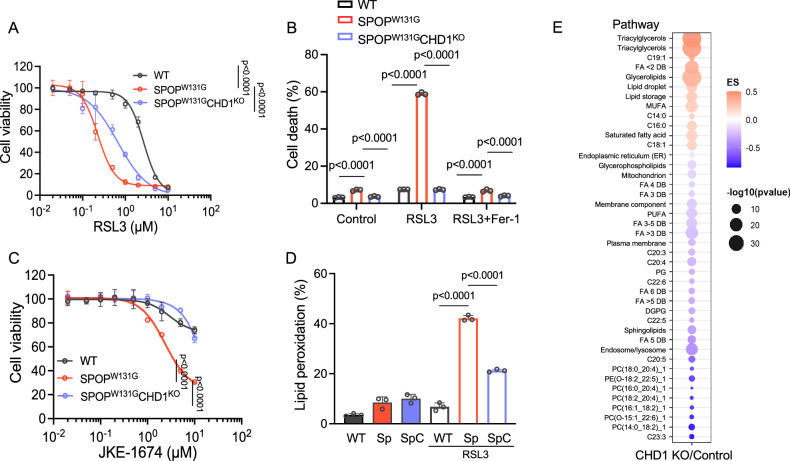
Loss of CHD1 confers ferroptosis resistance in SPOP-mutated PCa. **A** Dose-response curves of RSL3 in WT and SPOP^W131G^ LNCaP cells with or without CHD1 deletion. **B** PI staining indicates cell death in WT and SPOP^W131G^ LNCaP cells with or without CHD1 deletion after treatment with RSL3 (1 μM, 24 h), in the presence of ferrostatin-1 (Fer-1, 10 μM). **C** Dose-response curves of JKE-1674 in WT and SPOP^W131G^ LNCaP cells with or without CHD1 deletion. **D** Lipid peroxidation levels in WT and SPOP^W131G^ LNCaP cells with (SpC) or without (Sp) CHD1 deletion after treatment with RSL3 (1 μM, 24 h). **E** Lipidomic analysis showing decreased abundance of PUFA-containing phospholipids across multiple lipid classes in CHD1 knockout cells; *n* = 4 biological replicates. The significance of lipid ontology was assessed pairwise using Student’s *t*-test, followed by FDR correction. Data are presented as mean ± standard deviation, *n* = 3 biologically independent repeats unless otherwise stated. *P* values determined by one-way (**D**) or two-way (**A**–**C**) ANOVA analysis. Source data are provided as a Source Data file.

Given that nearly half of *SPOP*-mutated PCa harbor *CHD1* deletion, we next determined the impact of *CHD1* loss on ferroptosis sensitivity in this context. To this end, we genetically knocked out *CHD1* in *SPOP*-mutated LNCaP cells using CRISPR-Cas9 (Fig. S4A) and assessed their response to the GPX4 inhibitor RSL3. *CHD1* knockout reduced the sensitivity of *SPOP*-mutated LNCaP cells to RSL3 (Fig. 4A) and profoundly protected those cells from ferroptosis-mediated cell death (Figs. 4B and S4B). Similar effects were also observed in diverse *CHD1*-loss/*SPOP*-mutated PCa cells treated with other GPX4 inhibitors, JKE-1647 or ML162 (Figs. 4C and S4C–F). Notably, *CHD1* loss significantly impaired lipid peroxidation induced by *SPOP* mutations (Fig. 4D). In contrast to what was observed following *SPOP* mutation (Fig. 2A), lipidomic profiling analysis revealed that *CHD1* knockout led to a remarkable reduction of PUFA-containing phospholipids (Fig. 4E). Together, these results suggest that CHD1 plays a vital role in PUFA lipid metabolism, and *CHD1* loss confers ferroptosis resistance in *SPOP*-mutated PCa.

### CHD1 is required for the transcriptional activation of ACSL4 by interacting with c-Myc

**Fig. 5:**
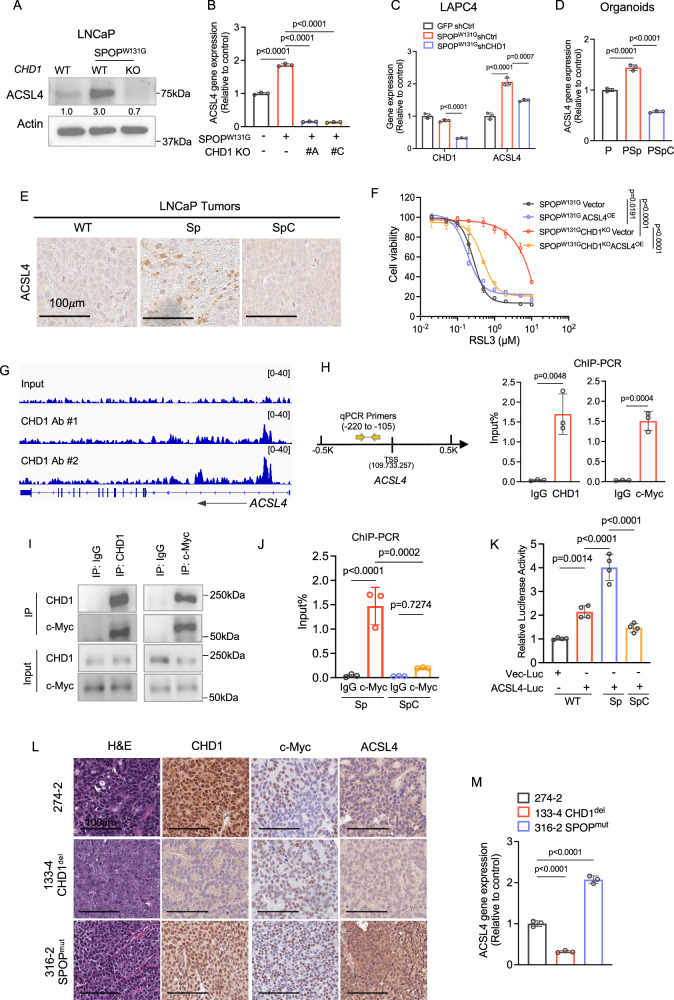
CHD1 is required for the transcriptional activation of ACSL4 by interacting with c-Myc. **A**, **B** CHD1 was knocked out in SPOP^W131G^ LNCaP cells using two sgRNAs (KO_A and KO_C), followed by the determination of ACSL4 protein (**A**) and mRNA (**B**) levels. Relative density ratios of indicated proteins/actin are shown. **C** mRNA expression of CHD1 and ACSL4 in WT and SPOP^W131G^ LAPC4 cells with or without CHD1 knockdown. **D** ACSL4 mRNA levels in tumor organoids derived from *Pten*^*L/L*^ (P), *Pten*^*L/L*^*;Spop*^*F133V*^ (PSp), and *Pten*^*L/L*^*;Spop*^*F133V*^*;CHD1*^*L/L*^ (PSpC) GEMMs. **E** IHC staining of ACSL4 in WT, SPOP^W131G^(Sp), SPOP^W131G^CHD1^KO^ (SpC) LNCaP-derived xenograft tumors. Scale bar, 100 μm. IHC staining was performed at least three times with similar results. **F** Dose-response curves of RSL3 in SPOP^W131G^ LNCaP cells with or without CHD1 KO and/or ACSL4 overexpression. **G** ChIP-Seq profile showing the binding of CHD1 to the promoter region of the *Acsl4* gene in murine PCa cells. Two antibodies against CHD1 were used for ChIP-seq. **H** ChIP-qPCR was performed in SPOP^W131G^ LNCaP cells using IgG control, CHD1, or c-Myc antibodies. The enrichment of CHD1 and c-Myc at the ACSL4 promoter are normalized to IgG control. The 1.5 k upstream or downstream of *ACSL4*’s transcription start site (TSS) and the location of primers are shown. **I** Co-IP assays were performed in SPOP-mutated LNCaP cells using CHD1 and c-Myc antibodies, followed by western blot analysis. The samples derive from the same experiment, but different gels for CHD1 and c-Myc were processed in parallel. This was performed at least three times with similar results. **J** ChIP-qPCR was performed in SPOP^W131G^ (Sp) and SPOP^W131G^CHD1^KO^ (SpC) LNCaP cells using IgG or c-Myc antibodies. The enrichment of c-Myc at the ACSL4 promoter are normalized to IgG control. **K** A luciferase reporter of the ACSL4 promoter was introduced into WT, Sp, SpC LNCaP cells, followed by luciferase assays. **L** Representative H&E and IHC staining of CHD1, c-Myc, and ACSL4 in three prostate cancer patient-derived xenograft (PDX) tumors: 274-2 (intact SPOP/CHD1), 133-4 (CHD1^del^), and 316-2 (SPOP^mut^). Scale bar, 100 μm. These experiments were performed at least three times with similar results. **M** qPCR analysis of ACSL4 mRNA expression in above PDX tumors. Data are presented as mean ± standard deviation, *n* = 3 biologically independent repeats. *P* values determined by unpaired two-tailed *t* test (**H**), one-way (**B**–**D**, **J**, **K**, **M**), or two-way (**F**) ANOVA analysis. Source data are provided as a Source Data file.

To uncover the role of CHD1 in ferroptosis, we first screened the expression of key ferroptosis regulators, including ACSL4, GPX4, SLC7A11, FSP1, NRF2, GCH1, and DHODH, in *SPOP*-mutated LNCaP cells with or without *CHD1* knockout. The results indicated that depletion of CHD1 remarkably reduced the expression of ACSL4 at both protein and mRNA levels and completely attenuated the ACSL4 induction by *SPOP* mutations (Figs. 5A, B and S5A, B). Consistently, knockout or knockdown of *CHD1* decreased ACSL4 levels in LAPC4 or 22Rv1 cells expressing SPOP mutants (Figs. 5C and S5C). This effect was further confirmed in P, PSp, and PSpC tumor organoids (Fig. 5D), as well as xenograft tumors derived from WT, SPOP^W131G^ (Sp), and SPOP^W131G^/CHD1^KO^ (SpC) LNCaP cells (Fig. 5E). In human PCa samples, we also observed that ACSL4 expression is positively correlated with CHD1, supporting a positive regulation (Fig. S5D).

Notably, dose-response analysis revealed that ACSL4 restoration re-sensitized CHD1-loss/SPOP-mutated cells to RSL3-induced ferroptosis (Figs. 5F and S5E, F), indicating ACSL4 is a downstream effector of CHD1 during ferroptosis. While searching our CHD1 ChIP-sequencing datasets in murine PCa cells^35^, we found that *ACSL4* is a direct target of CHD1 (Fig. 5G). ChIP-qPCR assays further confirmed that CHD1 directly binds to the promoter region of *ACSL4* in SPOP-mutated LNCaP cells (Fig. 5H). These findings suggest that CHD1 functions as a chromatin-associated activator of ACSL4 and plays a crucial role in ACSL4-mediated ferroptosis, particularly in *SPOP*-mutated PCa. Additionally, ChIP-sequencing analysis revealed that, compared to *SPOP*-WT cells, CHD1-binding sites across the genome were markedly increased in *SPOP*-mutated LNCaP cells, and nearly 90% of peaks were localized to the promoters of target genes (Fig. S5G, H). It suggests that *SPOP* mutations promote CHD1’s chromatin remodeling function in a more generic manner, implicating that *CHD1* contributes to the transcriptomic reprogramming in PCa harboring *SPOP* mutations.

Given CHD1’s chromatin remodeler role and its minimal effect on c-Myc expression (Fig. S5I), we hypothesize that CHD1 cooperates with the transcriptional factor c-Myc to induce ACSL4 expression in *SPOP*-mutated PCa. To test this hypothesis, we performed co-immunoprecipitation (co-IP) assays and found a physical interaction between CHD1 and c-Myc (Fig. 5I). Additional evidence was obtained from ChIP-PCR assays, which demonstrated co-localization of CHD1 and c-Myc at the *ACSL4* promoter (Fig. 5H). More importantly, loss of CHD1 abolished the binding of c-Myc protein to the *ACSL4* promoter (Fig. 5J). Additionally, luciferase assays revealed that CHD1 is required for *ACSL4* transcriptional activation in *SPOP*-mutated cells (Fig. 5K).

Furthermore, we assessed ACSL4 expression at the mRNA and protein levels in several MDA-PCa-PDX models harboring *SPOP* mutations or *CHD1* deletions, as previously reported^47^. Compared to MDA-PDX-274-2, which harbors intact *SPOP* and *CHD1*, *SPOP*-mutated MDA-PDX-316-2 exhibited a marked elevation of ACSL4 expression, whereas *CHD1*-deleted MDA-PDX-133-4 expressed a lower level of ACSL4 (Figs. 5L, M and S5J). Collectively, our mechanistic studies identify an underappreciated epigenetic/transcriptional complex composed of CHD1 and c-Myc and reveal its essential role in activating *ACSL4* transcription and triggering ferroptosis in SPOP-mutated cells. Conversely, loss of CHD1 abrogated c-Myc binding to the *ACSL4* gene, leading to *ACSL4* silencing and resistance to ferroptosis inducers.

### SPOP mutations promote, whereas CHD1 deletion reduces, the ferroptosis susceptibility in preclinical models of PCa

**Fig. 6:**
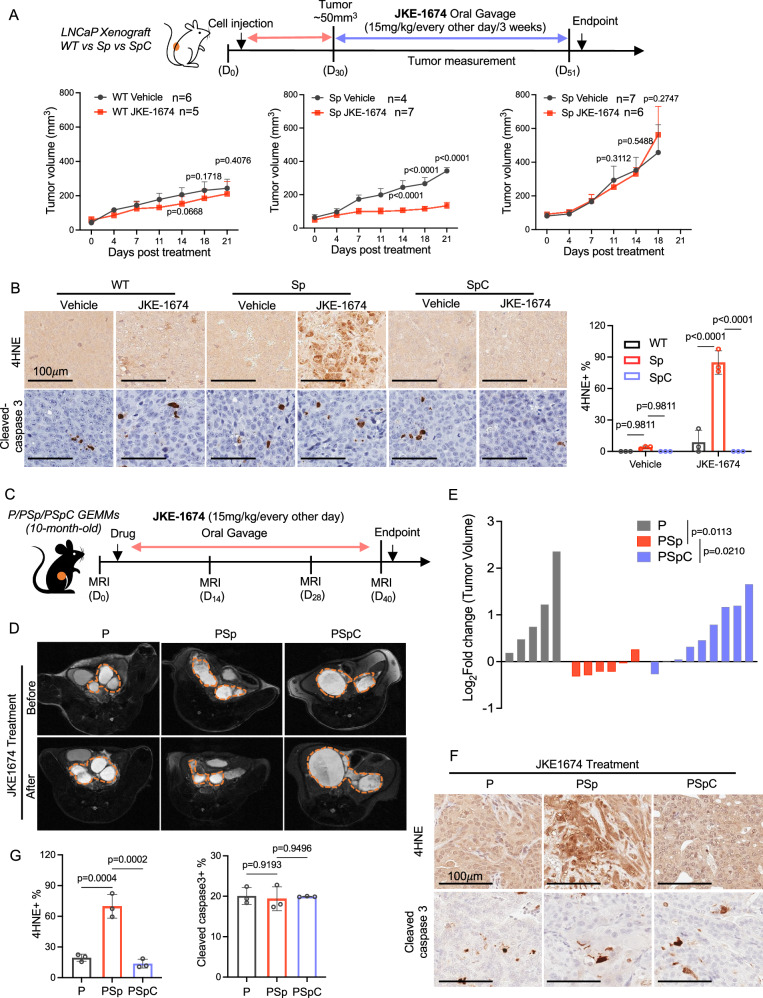
SPOP mutations promote, but CHD1 deletion reduces, the ferroptosis susceptibility in preclinical models of PCa. **A** Schematic of in vivo study design using wildtype (WT), SPOP^W131G^ (Sp), SPOP^W131G^/CHD1^KO^ (SpC) LNCaP xenograft models. 2 × 10^6^ WT, Sp, or SpC LNCaP cells were subcutaneously injected into both flanks of male SCID mice. After tumors reached ~50 mm^3^, tumor-bearing mice were randomized and treated with JKE-1674 (15 mg/kg; oral gavage) or vehicle control every other day for three weeks. Tumor volumes were measured twice a week using calipers. Tumor growth of each cohort is shown. Number of tumors in each group is presented. Created in BioRender. Chen, F. (2026) https://BioRender.com/hlh47iw. **B** IHC staining of 4HNE and cleaved caspase-3 in WT, Sp, and SpC xenograft tumors with or without JKE-1674 treatment. Scale bar, 100 μm. Quantification of 4HNE staining is shown. **C** Schematics of experimental design for JKE-1674 treatment in GEMMs. 10-month-old P (*n* = 5), PSp (*n* = 6), and PSpC (*n* = 9) GEMM mice were generated, followed by MRI imaging to confirm tumor formation. JKE-1674 (15 mg/kg) was administered orally every other day for 40 days. MRI imaging was used to monitor prostate tumor progression. Created in BioRender. Chen, F. (2026) https://BioRender.com/hlh47iw. **D** Representative MRI images of prostate tumors from P, PSp, and PSpC mice before and after JKE-1674 treatment. **E** The waterfall plot presents the Log2 fold change in prostate tumor volume at Day 40. **F**, **G** IHC staining and quantification of 4HNE and cleaved caspase-3 in P, PSp, and PSpC prostate tumors after JKE-1674 treatment. Scale bar, 100 μm. Data are presented as mean ± standard deviation, *n* = 3 biologically independent repeats unless otherwise stated. *P* values determined by unpaired two-tailed *t* test (**A**, **E**), one-way (**G**), or two-way (**B**) ANOVA analysis. Source data are provided as a Source Data file.

To assess the effects of *SPOP* mutation and *CHD1* loss on the responses to ferroptosis inducers in vivo, we established isogenic xenograft models using *SPOP*-mutated (SPOP^W131G^; Sp) and *SPOP*-mutated/*CHD1*-deleted (SPOP^W131G^/CHD1^KO^; SpC) LNCaP cells, alongside wild-type (WT) controls (Fig. 6A). JKE-1674 is a next-generation GPX4 inhibitor with improved metabolic stability and selectivity for ferroptosis induction^48^. After tumors reached the size of ~50 mm^3^, the tumor-bearing mice were treated with JKE-1674 (15 mg/kg, oral gavage, every other day) for three weeks, followed by tumor measurement twice a week using calipers.

Compared to the WT control, JKE-1674 treatment significantly suppressed tumor growth in the Sp group, but had minimal impact on SpC tumors (Figs. 6A and S6A). The mice tolerated the treatment well, as evidenced by stable body weight over time (Fig. S6B). IHC staining for 4-HNE and cleaved caspase-3 showed that JKE-1674 induced lipid peroxidation and ferroptosis exclusively in Sp tumors, while having little impact on apoptosis in any of the groups (Figs. 6B and S6C, D). To verify if the anti-tumor effect of JKE-1674 is mediated by ferroptosis, we co-administered JKE-1674 (25 mg/kg) with Liproxstatin-1 (Lip-1), a ferroptosis inhibitor, in another cohort of Sp xenograft tumors (Fig. S6E). The results indicated that co-treatment with Lip-1 effectively diminished the tumor-suppressive effect of JKE-1674 and protected *SPOP*-mutated tumors from ferroptosis (Fig. S6F–I), further validating ferroptosis as the primary mechanism of action.

Furthermore, we tested the efficacy of JKE-1674 in newly established P (*SPOP*^*WT*^*/CHD1*^*WT*^), PSp (*SPOP*^*F133V*^*/CHD1*^*WT*^), and PSpC (*SPOP*^*F133V*^*/CHD1*^*KO*^) GEMMs with single or combined defects of *SPOP* and *CHD1* (Figs. 6C and S6J). Ten-month-old mice in each cohort received JKE-1674 (15 mg/kg, oral gavage, every other day) for 40 days. MRI imaging was performed before and after treatment to monitor tumor progression. The results revealed that, compared to P mice, JKE-1674 treatment significantly suppressed tumor progression in PSp mice, whereas *CHD1* deletion completely attenuated the anti-tumor effects of JKE-1674 in PSpC mice (Fig. 6D, E). Histopathology analysis revealed a significant increase in lipid peroxidation and ferroptosis in PSp tumors following treatment, but not in P or PSpC tumors (Fig. 6F, G). In contrast, no significant change was observed in cleaved caspase-3 levels across all genotypes after treatment (Fig. 6F, G).

Together, these preclinical trials in xenograft models and GEMMs demonstrate that *SPOP* mutations potentiate the efficacy of GPX4 inhibitors in PCa; however, the co-occurrence of *CHD1* loss confers ferroptosis resistance in *SPOP*-mutated tumors, establishing the distinct effects of *SPOP* and *CHD1* on ferroptosis-targeted therapy.

### Cholesterol-lowering drugs restore ACSL4 expression in SPOP/CHD1 co-deficient PCa via activating SP1

Next, we sought to develop rational combinations to overcome ferroptosis resistance in *SPOP /CHD1* co-deficient PCa. We recently reported that *CHD1* loss-induced cholesterol biosynthesis facilitates intratumoral androgen production and retains AR activity, thereby conferring castration resistance in *SPOP*-mutated PCa^36^. Thus, enhanced cholesterol biosynthesis is a therapeutic vulnerability in PCa with *SPOP* and *CHD1* co-defects. Small-molecule inhibitors (statins) have been developed to target cholesterol biosynthesis, and some statins have been approved by the FDA for treating hypercholesterolemia, such as atorvastatin, rosuvastatin, and simvastatin^49^.

**Fig. 7:**
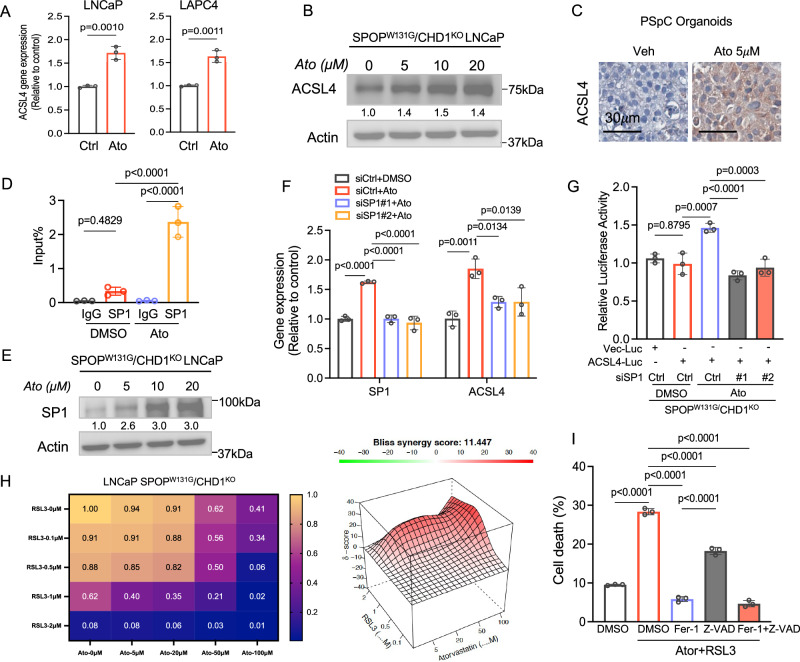
Cholesterol-lowering drugs restores ACSL4 in SPOP/CHD1 co-deficient PCa via activating SP1. **A** SPOP^W131G^/CHD1^KO^ LNCaP and SPOP^W131G^/CHD1^KD^ LAPC4 cells were treated with atorvastatin (10 μM, 48 h), followed by determining ACSL4 mRNA levels using qPCR. **B** Western blot analysis of ACSL4 in SPOP^W131G^/CHD1^KO^ LNCaP cells treated with different doses of atorvastatin for 48 h. Relative density ratios of indicated proteins/actin are shown. This was performed at least three times with similar results. **C** IHC analysis of ACSL4 expression in organoids derived from *Pten*^*L/L*^*; Spop*^*F133V*^*; CHD1*^*L/L*^ (PSpC) GEMM tumors treated with atorvastatin (5 μM). Scale bar, 30 μm. IHC staining was performed at least three times with similar results. **D** SPOP^W131G^/CHD1^KO^ LNCaP cells were treated with or without atorvastatin (10 μM) for 48 h, followed by ChIP-qPCR using IgG control or SP1 antibodies. The enrichment of SP1 at the ACSL4 promoter are normalized to IgG control. **E** Western blot analysis of SP1 in SPOP^W131G^/CHD1^KO^ LNCaP cells after atorvastatin treatment for 48 h. Relative density ratios of indicated proteins/actin are shown. **F**, **G** SP1 was knocked down in SPOP^W131G^/CHD1^KO^ LNCaP cells, followed by treatment with atorvastatin (10 μM) for 48 h. qPCR assay was performed to determine SP1 and ACSL4 mRNA levels (**F**). Luciferase assays were performed to determine the transcriptional activity of the ACSL4 promoter (**G**). **H** Dose-response matrix (left) and drug interaction landscapes (right) revealing synergistic effects of RSL3 and atorvastatin on SPOP^W131G^/CHD1^KO^ LNCaP cells. Drug interaction landscapes and synergy scores were generated using SynergyFinder. **I** PI staining of SPOP^W131G^/CHD1^KO^ LNCaP cells after combination treatment of RSL3 and atorvastatin, with or without ferrostatin-1 (Fer-1, 10 μM) or Z-VAD-FMK (Z-VAD, 10 μM). Data are presented as mean ± standard deviation, *n* = 3 biologically independent repeats. *P* values determined by unpaired two-tailed *t* test (**A**, **E**) or one-way (**F**, **G**, **I**) ANOVA analysis. Source data are provided as a Source Data file.

Interestingly, we found that inhibiting cholesterol biosynthesis with atorvastatin significantly induced ACSL4 expression in *SPOP/CHD1* co-deficient PCa cells and tumor organoids (Fig. 7A–C). Transcriptional factor SP1 is involved in various biological processes. Prior studies reported that cholesterol depletion activates SP1-dependent gene expression^50^. A recent study reported that ACSL4 is a target gene of SP1^51^. Our ChIP-qPCR assays verified that SP1 indeed binds to the promoter of *ACSL4*, and its binding is further enriched in the presence of cholesterol-lowering drug treatment (Fig. 7D). Furthermore, our data showed that atorvastatin induced SP1 expression in *CHD1*-deficient cells (Figs. 7E and S7A) but had little impact on SP1’s protein stability or phosphorylation (Fig. S7B, C). More importantly, knocking down SP1 in *SPOP*-mutated/*CHD1*-deleted LNCaP cells impaired atorvastatin-mediated ACSL4 overexpression (Figs. 7F, G and S7D). Clinically, ACSL4 expression is positively correlated with SP1 in human PCa samples (Fig. S7E). These findings establish a *CHD1*-independent ACSL4 regulatory mechanism, i.e., cholesterol-SP1-ACSL4, providing a molecular basis for understanding synergistic effects between cholesterol-lowering drugs and ferroptosis inducers in *SPOP/CHD1* co-deficient PCa cells. Interestingly, ChIP-seq analysis indicated that cholesterol-lowering drug treatment markedly increased SP1-binding sites at gene promoters across the genome in *SPOP/CHD1* co-deficient PCa LNCaP cells (Fig. S7F, G), suggesting a broad effect of cholesterol on the SP1 transcriptome.

### Cholesterol-lowering drugs re-sensitize SPOP/CHD1 co-deficient PCa to ferroptosis

Given that *CHD1* loss confers ferroptosis resistance by repressing *ACSL4* transcription (Fig. 5), we hypothesize that atorvastatin-induced ACSL4 expression can resensitize *SPOP/CHD1* co-deficient prostate tumors to ferroptosis-targeted therapy. To test this hypothesis, we determined the efficacy of RSL3 in combination with atorvastatin in LNCaP and LAPC4 cells. The results indicated that RSL3 and atorvastatin have synergistic effects on killing *SPOP/CHD1* co-deficient cancer cells, as indicated by Bliss synergy tests (Figs. 7H and S7H). To define the types of cell death responsible for this synergy, we employed pharmacologic inhibition of ferroptosis (Fer-1) and/or apoptosis (Z-VAD-FMK). Cell viability analysis showed that Z-VAD-FMK treatment partially impaired the cell death induced by the combination; in contrast, Fer-1 alone or combined with Z-VAD-FMK was able to thoroughly protect *SPOP/CHD1* co-deficient cells from death (Fig. 7I), indicating that ferroptosis plays a predominant role in mediating cell death induced by RSL3/atorvastatin combination. Of note, RSL3 also showed a synergistic effect with the AR inhibitor Enzalutamide in *SPOP*-mutated PCa cells (Fig. S7I), suggesting the therapeutic potential of co-targeting ferroptosis and AR signaling in this context.

**Fig. 8:**
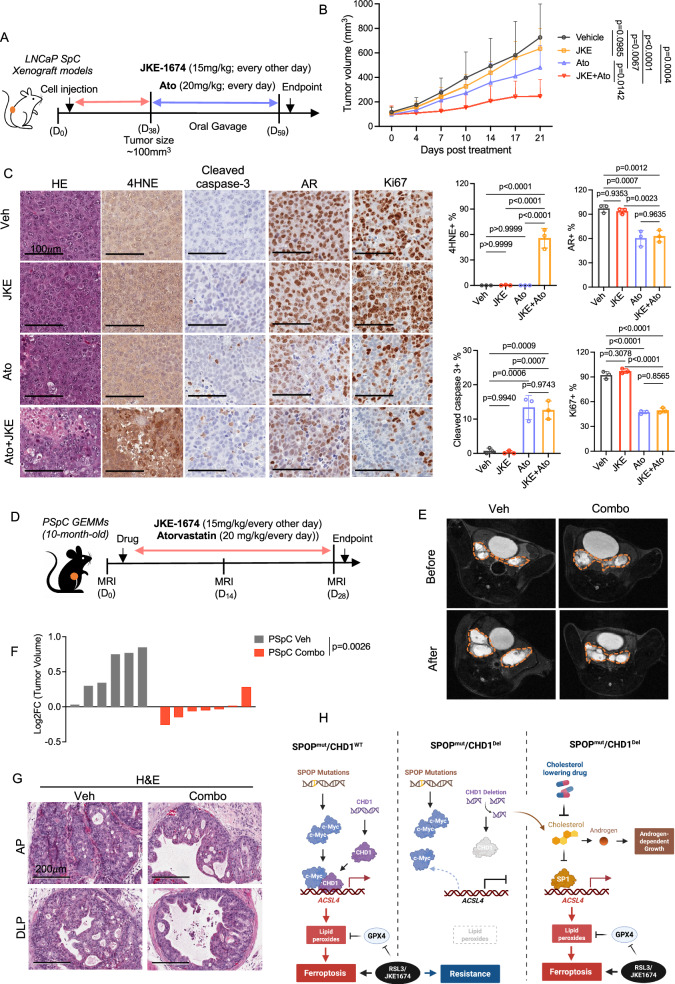
Cholesterol-lowering drugs re-sensitize SPOP/CHD1 co-deficient PCa to ferroptosis. **A** Schematic of combination treatment study design in xenograft models. 2 × 10^6^ SPOP^W131G^/CHD1^KO^ (SpC) LNCaP cells were subcutaneously injected into both flanks of male SCID mice. After tumors reached ~100 mm^3^, tumor-bearing mice were randomized and orally administered with JKE-1674 (15 mg/kg; every other day) and atorvastatin (20 mg/kg; every day), as single agents or in combination. Created in BioRender. Chen, F. (2026) https://BioRender.com/hlh47iw. **B** Growth of SpC xenograft tumors after treatment (Vehicle *n* = 5, JKE *n* = 7, Ato *n* = 8, JKE + Ato *n* = 7). **C** H&E, IHC staining, and quantification of indicated markers in SpC xenograft tumors after treatment. Scale bar, 100 μm. **D** Schematic of combination treatment study design in GEMMs. 10-month-old PSpC GEMM mice were imaged by 7T-MRI to confirm tumor formation, followed by oral administration of JKE-1674 (15 mg/kg, every other day) and atorvastatin (20 mg/kg/day) for 4 weeks. Tumor progression was monitored by biweekly MRI imaging. Created in BioRender. Chen, F. (2026) https://BioRender.com/hlh47iw. **E** Representative MRI images of PSpC tumors before and after vehicle (Veh) or combination treatment (Combo). Tumor areas are indicated by dashed outlines. **F** Waterfall plot of log2 fold change in tumor volume for individual PSpC tumors in the Veh (*n* = 6) and Combo (*n* = 7) groups. **G** Representative histological staining of PSpC tumors from the anterior prostate (AP) and dorsolateral prostate (DLP) lobes treated with Veh or Combo. Scale bar, 200 μm. Data are presented as mean ± standard deviation, *n* = 3 biologically independent repeats. *P* values determined by unpaired two-tailed *t* test (**F**) or one-way (**B**, **C**) ANOVA analysis; *P < 0.05; **P < 0.01; ***P < 0.001; ****P < 0.0001; ns not significant. **H** Schematic working model. Loss-of-function SPOP mutations lead to the stabilization of the c-Myc protein, which interacts with the chromatin remodeler CHD1 to activate transcription of ACSL4, thereby inducing lipid peroxidation and increasing ferroptosis sensitivity. In contrast, CHD1 deletion impairs the binding of c-Myc to the ACSL4 promoter, resulting in ACSL4 silencing and conferring ferroptosis resistance in SPOP-mutated PCa. CHD1 loss promotes cholesterol biosynthesis and intratumoral androgen production, thereby facilitating the androgen-dependent growth of SPOP-mutated PCa. Cholesterol-lowering drugs (e.g., statins) not only attenuate androgen production but also restore ACSL4 expression by upregulating its transcriptional factor SP1. Co-targeting cholesterol and ferroptosis has synergistic anti-tumor effects in SPOP/CHD1 co-deficient PCa. Created in BioRender. Zhao, D. (2026) https://BioRender.com/ldhwvx4. Source data are provided as a Source Data file.

Next, we evaluated the efficacy of this combination in the LNCaP xenograft model with *SPOP* mutations and *CHD1* deletion. When tumors reached ~100 mm³, tumor-bearing mice were treated with vehicle, JKE-1674 (15 mg/kg, every other day), atorvastatin (20 mg/kg, every day), or in combination for three weeks (Fig. 8A). All regimens were well tolerated (Fig. S8A). Notably, *SPOP/CHD1* co-deficient tumors exhibited the greatest response to the combination of JKE-1674 and atorvastatin compared with single-agent treatment (Fig. 8B). Histopathology analyses showed that atorvastatin alone or combined with JKE-1674 significantly reduced AR nuclear localization, inhibited cell proliferation, and caused apoptosis; whereas only combination treatment markedly induced ferroptosis in *SPOP/CHD1* co-deficient PCa, characterized by high 4-HNE expression (Fig. 8C). Consistent with our mechanistic studies, we also observed SP1 upregulation upon atorvastatin treatment in CHD1-deficient tumors (Fig. S8B, C).

Finally, we tested the combination of JKE-1674 and atorvastatin in our PSpC GEMM (Fig. 8D). The results revealed that these *SPOP/CHD1* co-deficient prostate tumors responded well to the combination treatment, characterized by impaired tumor growth (Fig. 8E, F), less invasive phenotypes (Figs. 8G and S8D), and reduced cancer cell proliferation (Fig. S8D). However, the combination has minimal effects on lineage differentiation in *SPOP/CHD1* co-deficient PCa cells (Fig. S8D). Together, these mechanistic studies establish a regulatory axis of cholesterol–SP1–ACSL4 and its role in controlling ferroptosis, particularly in *CHD1*-deficient tumors. More importantly, our preclinical trials demonstrate the therapeutic potential of combining ferroptosis inducers with cholesterol-lowering drugs in PCa containing *SPOP/CHD1* co-defects.

## Discussion

As a novel iron-dependent form of programmed cell death, ferroptosis has garnered considerable attention due to its therapeutic benefits across different cancer types, particularly those with specific molecular features^40–44^. In this study, we reported *SPOP* mutations and *CHD1* deletion as key genetic determinants of ferroptosis susceptibility. Using PCa as a model system, we demonstrate that *SPOP* mutations enhance, whereas *CHD1* deletion impairs, the efficacy of ferroptosis inducers targeting GPX4 (Fig. 8H). Our mechanistic studies revealed that loss-of-function *SPOP* mutations lead to protein stabilization of transcriptional factor c-Myc, which interacts with the chromatin remodeler CHD1 to activate *ACSL4* gene transcription. The *SPOP* mutation-driven and *CHD1*-dependent elevation of ACSL4 triggers lipid peroxidation, thereby promoting ferroptosis sensitivity to GPX4 inhibitors (Fig. 8H). In contrast, *CHD1* deletion attenuated c-Myc’s binding to the *ACSL4* promoter, resulting in *ACSL4* silencing and conferring ferroptosis resistance in *SPOP*-mutated PCa (Fig. 8H). Furthermore, we identified a c-Myc/CHD1-independent transcriptional regulatory axis, i.e., cholesterol–SP1–ACSL4, and uncovered that cholesterol-lowering drugs can restore ACSL4 expression by upregulating transcriptional factor SP1 in *CHD1*-deficient PCa (Fig. 8H). Most importantly, our preclinical studies provided proof-of-concept evidence supporting that co-targeting ferroptosis and cholesterol biosynthesis has synergistic therapeutic effects in *SPOP/CHD1* co-deficient PCa, through triggering ferroptotic cell death and attenuating androgen-dependent tumor growth.

Ferroptosis-inducing therapies necessitate a comprehensive understanding of ferroptosis biology, encompassing upstream genetic events, nutritional conditions, and the epigenetic and transcriptional regulation of downstream effectors. Our studies uncovered two pathways that modulate the biological process of ferroptosis in different contexts: one is the antagonistic regulatory axis of SPOP–c-MYC/CHD1–ACSL4, and the other is a nutrition-based mechanism involving cholesterol–SP1–ACSL4. Despite a recent study reporting c-Myc as a transcriptional factor for ACSL4^46^, the upstream regulators, epigenetic machinery, and the physiological and pathological conditions involved in this process remain to be identified. Our studies bridged these knowledge gaps by (1) identifying CHD1 as an epigenetic partner of c-Myc in priming the accessibility of the ACSL4 gene and facilitating the binding of c-Myc and the transcriptional activation of ACSL4; (2) elucidating SPOP’s negative regulation on ACSL4 by mediating c-Myc’s proteolysis; (3) establishing the contrary effects of genetic defects in *SPOP* and *CHD1* on ACSL4-mediated lipid peroxidation and ferroptosis. Cholesterol levels in culture medium were found to affect ferroptosis sensitivity^52^; however, the underlying mechanism remains elusive. Here, we discovered that the transcriptional factor SP1 acts as a sensor of low environmental cholesterol levels and triggers ACSL4 expression in a c-Myc/CHD1-independent manner. It is worth noting that these two pathways interact with each other, as CHD1 controls cholesterol biosynthesis^36^. In addition to c-Myc dissociation, *CHD1* loss-driven cholesterol production also suppresses ACSL4, creating a metabolic vulnerability for targeting cholesterol in *CHD1*-deficient tumors and facilitating synergistic anti-tumor effects with ferroptosis inducers. These mechanistic studies not only advance our understanding of ferroptosis biology but also lay an essential foundation for effectively targeting ferroptosis in different molecular subtypes of advanced PCa.

Increasing evidence showed the therapeutic potential of ferroptosis in PCa^43^. Although the loss of the tumor suppressor *RB1* has been reported to be associated with increased sensitivity to ferroptosis in PCa^41^, much remains unknown about which molecular subsets of patients will benefit most from ferroptosis-targeted therapies. Here, we demonstrate that *SPOP*-mutated cancer cells are more susceptible to ferroptosis inducers targeting GPX4, indicating SPOP has prognostic value in PCa patients receiving GPX4 inhibitors. Clinically, *SPOP* genetic status has been used as a biomarker to predict PCa patients’ response to castration or AR-targeted therap^19–23^, reinforcing its feasibility for ferroptosis-focused trials. Conversely, androgen signaling was also linked to anti-ferroptosis effects via upregulating phospholipid-modifying enzymes MBOAT1/2, a GPX4-independent mechanism^44^. The combination with AR antagonists potentiated the anti-tumor effects of ferroptosis inducers^44^. Interestingly, our studies revealed that *SPOP* mutations sensitize PCa to GPX4 inhibitors in an androgen-independent manner, suggesting GPX4 inhibitors remain effective under castration conditions. Also, our data suggested that the combination of ferroptosis inducers and AR antagonist (Enzalutamide) had a strong synergistic effect in *SPOP*-mutated PCa (Fig. S8E), providing a rationale for co-targeting these two pathways in PCa patients with *SPOP* defects. Of note, there are two major classes of ferroptosis inducers^37,53,54^: one targets GPX4 (such as RSL3, ML162, and JKE1674), which are used in our studies; the other targets the cystine/glutamate transporter SLC7A11 (such as erastin). A recent study reported that *SPOP* mutations induce SLC7A11 expression by stabilizing the epigenetic factor JMJD6, resulting in resistance to erastin^55^. The distinct responses of *SPOP*-mutated cancer cells to different classes of ferroptosis inducers align well with the finding that ACSL4 is more essential for ferroptosis triggered by GPX4 inhibitors, compared to cysteine depletion or erastin^56^. Therefore, caution should be taken when selecting ferroptosis inducers for treating PCa patients, particularly those with *SPOP* defects.

Beyond *SPOP*, we identified *CHD1* loss as another key genetic determinant of ferroptosis sensitivity that, in contrast, served as a predictive biomarker of ferroptosis resistance. This information is crucial because *CHD1* deletion occurs in nearly half of *SPOP*-mutated PCa, underscoring that precision oncology intervention requires more precise characterization of biomarkers and molecular subtypes. The divergent effects of *SPOP* mutations and *CHD1* deletion on therapeutic responses were also observed in our recent studies, in which we found that *CHD1* loss confers castration resistance in *SPOP*-mutated PCa by promoting cholesterol biosynthesis^36^. Those therapy-resistant phenotypes also highlight the urgent need for effective, combinatorial treatment for *SPOP*-mutated/*CHD1*-deleted PCa. By determining the role of cholesterol in repressing *ACSL4* transcription and the underlying mechanisms, we developed a therapeutic strategy for this therapy-resistant molecular subtype: combining ferroptosis inducers with FDA-approved, cholesterol-lowering drugs. In preclinical models, the combination showed synergistic and effective anti-tumor effects by inducing ferroptosis and suppressing AR-dependent tumor growth in *SPOP/CHD1* co-deficient PCa. The efficacy of this combination in castration-resistant PCa is yet to be determined. Given the safety and broad use of cholesterol-lowering drugs in the clinic, our studies present promising translational potential and will inform the design of future clinical trials targeting ferroptosis in advanced PCa.

Given the high frequency and clinical impact of *SPOP* mutations and *CHD1* deletion in PCa, our studies primarily focused on PCa models. Of note, genetic deletion or mutations of *CHD1* are frequently observed in estrogen-dependent tumors^27^. Given the vital role of cholesterol in estrogen biosynthesis, co-targeting ferroptosis and cholesterol biosynthesis might also be effective in estrogen-dependent cancers with *CHD1* defects. In addition to drug treatment, our studies suggest that diet and nutritional management may be an alternative means of controlling cholesterol uptake and metabolism in certain patient subsets receiving ferroptosis-inducing therapies.

In summary, our findings establish SPOP and CHD1 as key upstream regulators of the ferroptosis pathway and genetic determinants of ferroptosis susceptibility. Our preclinical studies also provide biomarker-driven combinatorial strategies to enhance ferroptosis-based therapy in advanced PCa.

## Methods

All mouse experiments were approved by the MD Anderson Institutional Animal Care and Use Committee (IACUC, protocol #00001955) and conducted in accordance with institutional guidelines. Mice were housed in a controlled environment (12-h light/dark cycle, 68–78 °F, 30–70% humidity). Mice were fed ad libitum with a standard Purina 5053 Chow and had unrestricted access to drinking water. The following criteria were used to determine humane endpoints: (a) subcutaneous tumors were limited to ≤2.0 cm in diameter; (b) prostate tumors caused urinary obstruction or incontinence. Animals will be carefully monitored and will be euthanized if they show overt signs of distress (weight loss, poor grooming, decreased activity) or neurological dysfunction (falling, circling, paralysis). Given the relevance to prostate cancer biology, only male mice were used.

### Cell culture

All cell lines, except for the human prostate cancer LAPC4 cell line, were originally obtained from ATCC. The LAPC4 cell line was a generous gift from Dr. Frigo’s lab. All cell lines were verified to be devoid of mycoplasma contamination. All cells were nurtured at 37 °C with 5% CO_2_. LNCaP and 22Rv-1 cells were cultured in RPMI 1640 medium (Corning, 10-040-CV) containing 10% (v/v) fetal bovine serum (FBS) (Gibco, 10082147) and 1% Pen-Strep. LAPC4 were cultured with IMDM (Invitrogen, 12440-053) containing 15% FBS, 1% Pen-Strep, 1% L-glutamine (Gibco, 25030081), and 1 nM DHT (Cerilliant, D-073). DU145 cells were cultured in EMEM (ATCC: The Global Bioresource Center, 30-2003) supplemented with 10% FBS and 1% Pen-Strep. 293T cells were cultured in DMEM (Corning, 10-017-CV) supplemented with 10% FBS and 1% Pen-Strep. To reduce the androgen level in the medium and mimic the castration conditions, Charcoal-Stripped FBS (Gibco, 12676029) was used to replace the regular FBS with the same supplement percentage.

### Dose-response assays

Cells were seeded onto 96-well plates (Corning, 3903) and treated with a serial dilution range of ferroptosis inducers, including RSL3 (Selleckchem, S8155), ML162 (Cayman Chemical, 20455), ML-210 (Cayman, 23282), and JKE-1674 (MedChemExpress, HY-138153), for 24 h. The CellTiter-Glo Luminescent Cell Viability Assay kit (Promega, G7573) was used following the manufacturer’s recommendations. The viability of cells in each well was standardized against the DMSO (Thermo Fisher Scientific, BP231-100) control group. The IC50 values were analyzed using GraphPad Prism version 10. To evaluate potential drug synergy, cells were treated with a matrix combination of JKE-1674 and Atorvastatin (Selleckchem, S2077) across a range of concentrations for 24 h. Cell viability was assessed using the CellTiter-Glo assay, and the synergistic effects between the two drugs were analyzed using SynergyFinder (v3.0). A synergy score greater than 10 was considered indicative of a synergistic interaction.

### Cell death assays

To assess cell death, 50,000 cells were seeded into 12-well plates (Genesee Scientific, 25–106) two days prior to treatment. Following treatment with the indicated compounds, including ferroptosis inhibitors and cell death inhibitors such as ferrostatin-1 (Sigma-Aldrich, SML0583) or Z-VAD-FMK (Selleckchem, S7023), both adherent and floating cells were harvested by trypsinization, washed once with PBS, and stained with 2 μg/mL propidium iodide (PI; Invitrogen, 2159639) in cold PBS. PI-positive (dead) cells were quantified using an Attune NxT flow cytometer (Invitrogen) and analyzed with FlowJo v10 software.

### Lipid peroxidation measurement

Cells were seeded in 12-well plates and treated with DMSO or the indicated compounds. After 24 h, cells were collected by trypsinization and resuspended in 500 μL PBS containing 5 μM C11-BODIPY 581/591 (Invitrogen, D3861). Following a 30-min incubation at 37 °C in a water bath, lipid peroxidation was quantified by flow cytometry using oxidized C11-BODIPY 581/591 fluorescence (excitation: 488 nm; emission: 530/30 nm). At least 10,000 single-cell events were recorded per condition.

### Organoid establishment, culture, and treatment

Prostate tumor organoids were established from GEMM-derived tumors as described previously^36,57^. Briefly, dissected tumors were minced and digested with collagenase II (Gibco, 17101-015) at 37 °C for 1–2 h, followed by TrypLE Express (Gibco, 12605-010) for single-cell dissociation. Cells were embedded in 75% Matrigel (BD Biosciences, 356231) and cultured in mouse prostate organoid medium. Organoids were passaged weekly using TrypLE. For drug treatment, organoids were released from Matrigel using Cell Recovery Solution (Corning, 354253) and dissociated into single cells using TrypLE supplemented with Y-27632 dihydrochloride. 1000 single cells were seeded in 40 μL of Matrigel per well in 24-well plates (Costar, 09-761-146) and cultured for 24 h before compound treatment. Media and compounds were refreshed on day 4. On day 6, GFP-positive organoids were imaged using a Cytation 5 Cell Imaging Multi-Mode Reader (BioTek). Organoids were then harvested from Matrigel using Cell Recovery Solution (1 h on ice), washed twice with cold PBS, and processed for downstream analyses. For IC50 determination, 1000 cells were seeded in 40 μL Matrigel per well in 24-well plates and cultured for 24 h, followed by treatment with DMSO or RSL3 at serial concentrations with three replicates for 6 days. On day 6, organoids were digested into single cells, and viability was measured using the CellTiter-Glo Luminescent Cell Viability Assay kit according to the manufacturer’s instructions and described above.

### Transient transfection and lentiviral transduction

Transient siRNA transfections were performed using Lipofectamine 2000 (Thermo Fisher Scientific, 11668019) according to the manufacturer’s instructions. Lentiviral particles were generated by co-transfecting 293T cells with lentiviral expression constructs, psPAX2, and pMD2.G using PolyJet transfection reagent (SignaGen Laboratories, SL100688). Target cells were transduced in the presence of 10 μg/mL polybrene (EMD Millipore, TR-1003-G) and subsequently selected with 10 μg/mL puromycin (Gibco, A1113803), or 20 μg/mL blasticidin (InvivoGen, ant-bl-05), or sorted via FACSAria II Cell Sorter (BD Biosciences). Sequences of all siRNAs, sgRNAs, and shRNAs are provided in Supplementary Data 3.

### Generation of CHD1 or ACSL4 knockout cell lines using CRISPR

CHD1 knockout LNCaP cells were generated in our prior studies using sgRNA-guided CRISPR-Cas9 system^34,36,58^. As described, X330-mCherry plasmids encoding sgRNAs targeting human *CHD1* (#1 and #3) were transiently transfected into LNCaP cells. mCherry-positive cells were sorted using FACSAria II Cell Sorter (BD), followed by single-cell cloning and expansion. CHD1 deletion was confirmed by Western blot analysis. Two *CHD1* knockout sublines, KO-A and KO-C, were established from sgRNAs #1 and #3, respectively. Genetic deletion of CHD1 in both sublines was verified using PCR-sequencing. Since both *CHD1* KO-A and KO-C exhibited similar effects on ACSL4, KO-C was primarily used for subsequent in vitro and in vivo functional studies. Parental LNCaP cells served as the control. ACSL4 knockout in LNCaP cells was achieved using the lentiviral vector lentiCRISPRv2 encoding ACSL4-targeting sgRNAs. The plasmids were co-transfected into 293T cells with psPAX2 and pMD2.G to produce lentiviral particles. LNCaP cells were infected with the viral supernatant in the presence of 10 μg/mL polybrene and selected with 20 μg/mL blasticidin (InvivoGen) for one week. Blasticidin-resistant cells were expanded, and knockout efficiency was confirmed by Western blot analysis. The sequences of sgRNAs are listed in Supplementary Data 3.

### Histology and immunohistochemistry

Prostate tumors were collected, fixed in formalin, and paraffin-embedded. H&E and immunohistochemical (IHC) staining were performed following standard protocols^36^. The following primary antibodies were used: anti-4-HNE (Abcam, ab46545, 1:400), anti-ACSL4 (Santa Cruz, sc-271800, 1:200), anti-CHD1 (Sigma-Aldrich, HPA022236, 1:500), anti-Flag (Cell signaling technology (CST), 14793, 1:500), anti-cleaved caspase-3 (CST, 9661, 1:500), anti-AR (Sigma-Aldrich, 06-680, 1:500), anti-Ki67 (Thermo Fisher, RM-9106-S1, 1:500), and anti-SP1 (CST, 9389, 1:100). Detailed information on antibodies is listed in Supplementary Data 3. Slides were scanned using an Aperio CS2 ScanScope (Leica) and analyzed with ImageScope software (v12.0.0). Quantification of IHC staining was performed using QuPath (v0.6.0-rc5)^59^ on images acquired at 20× magnification for tumor tissues and 40× magnification for organoids.

### Western blot analysis

The experiments were performed as in our previous studies^36^. In brief, cell pellets were lysed in 1× Laemmli sample buffer (Bio-Rad, 1610747) with 2-mercaptoethanol (Bio-Rad, 1610710), followed by incubation at 99 °C for 15 min. Proteins were separated with 4% to 15% Mini-PROTEAN TGX Precast Protein Gels (Bio-Rad, 4561086) and transferred to the nitrocellulose membrane using a Trans-Blot Turbo RTA Mini 0.2-μm nitrocellulose transfer kit (Bio-Rad, 1704270). After 1 h of blocking in 5% non-fat milk at ambient temperature, the membranes were exposed to specific primary antibodies at 4 °C overnight. Subsequently, the membranes underwent incubation with horseradish peroxidase (HRP)-conjugated secondary antibodies at ambient temperature for 1 h. Colorimetric and chemiluminescence signals were developed with Western ECL substrates (Bio-Rad, 1705060) and captured using the ChemiDoc Imaging System (Bio-Rad). The antibodies employed included anti-ACSL4 (Santa Cruz, sc-271800, 1:1000), anti-CHD1 (D8C2) (CST, 4351, 1:1000), anti-c-Myc (CST, 18583, 1:1000), anti-SP1 (CST, 9389, 1:1000), anti-β-actin (Sigma, A5441, 1:5000), anti-V5 Tag (Invitrogen, 46-1157, 1:1000), anti-GPX4 (R&D Systems, MAB5457, 1:1000), anti-SLC7A11 (CST, 12691S, 1:1000), anti-DHODH (Proteintech, 14877-1-AP, 1:1000), anti-NRF2 (CST, 12721, 1:1000), anti-GCH1 (Invitrogen, MA5-27277, 1:1000), anti-FSP1 (Proteintech, 20886-1-AP, 1:1000), anti-rabbit IgG (HRP) (CST, 7074 V, 1:5000), and anti-mouse IgG (HRP) (CST, 7076 V, 1:5000). Detailed information on antibodies is listed in Supplementary Data 3.

### Quantitative real-time PCR (qRT-PCR)

Total RNA was isolated using the RNeasy Mini Kit (Qiagen, 74106), and reverse transcription was performed with the High-Capacity cDNA Reverse Transcription Kit (Applied Biosystems, 4368813) to generate complementary DNA. Quantitative real-time PCR was conducted using PowerUp SYBR Green Master Mix (Applied Biosystems, A25779) on a QuantStudio 3 Real-Time PCR System (Thermo Fisher Scientific, A28567). Transcript levels were normalized to ACTB, and data were analyzed using GraphPad Prism v10. The sequences of qPCR primers are listed in Supplementary Data 3.

### Site-directed mutagenesis

V5-tagged SPOP mutant constructs (W131G, F102C, and F133V) were generated as previously described^36^ using the Q5 Site-Directed Mutagenesis Kit (New England BioLabs, E0554S), with the V5-tagged WT-SPOP plasmid serving as the template. Mutagenesis primers were designed using the NEBaseChanger tool (http://nebasechanger.neb.com/). Plasmids were prepared using the QIAprep Spin Miniprep Kit (Qiagen, 27106) and sequence-verified by the Sequencing and Microarray Facility at MD Anderson.

### Untargeted lipidomic analysis by LC–MS/MS

LNCaP cells expressing wild-type or mutated SPOP were used for lipidomic profiling with four biological replicates. Approximately 10 million cells per sample were collected and sent to the Metabolomics Core Facility at MD Anderson for processing and analysis. Upon harvest, cells were washed with ice-cold 0.85% ammonium bicarbonate in deionized water. Cells were then scraped down and spun down at 400 × *g* for 3 min. The supernatant was removed, and the cells were snap-frozen in liquid nitrogen and stored at −80 °C. To determine the relative abundance of lipids across groups, extracts were prepared and analyzed using high-resolution mass spectrometry-based lipidomics at the Metabolomics Core Facility. Briefly, for each sample, 200 µL of extraction solution containing 2% Avanti SPLASH® LIPIDOMIX® Mass Spec Standard, 1% 10 mM butylated hydroxytoluene in ethanol was added, and the tubes were vortexed for 10 min. The tubes were placed on ice for 10 min, then centrifuged at 16,000 × *g* for 10 min at 4 °C. The supernatant was transferred to a glass autosampler vial, and the injection volume was 10 µL. Mobile phase A (MPA) was 40:60 acetonitrile: water with 0.1% formic acid and 10 mM ammonium formate. Mobile phase B (MPB) was 90:9:1 isopropanol:acetonitrile: water with 0.1% formic acid and 10 mM ammonium formate. The chromatographic method included a Thermo Fisher Scientific Accucore C30 column (2.6 µm, 150 × 2.1 mm) maintained at 40 °C, autosampler tray chilling at 8 °C, a mobile phase flow rate of 0.200 mL/min, and a gradient elution program as follows: 0–3 min, 30% MPB; 3–13 min, 30–43% MPB; 13.1–33 min, 50–70% MPB; 48–55 min, 99% MPB; 55.1–60 min, 30% MPB. A Thermo Fisher Scientific Orbitrap Fusion Lumos Tribrid mass spectrometer with a heated electrospray ionization source was operated in data-dependent acquisition mode, in both positive and negative ionization modes, with scan ranges of 150–827 and 825–1500 m/z. An Orbitrap resolution of 120,000 (FWHM) was used for MS1 acquisition, and a spray voltage of 3600 and −2900 V were used for positive and negative ionization modes. The vaporizer and ion transfer tube temperatures were set to 275 and 300 °C, respectively. The sheath, auxiliary, and sweep gas pressures were 35, 10, and 0 (arbitrary units), respectively. For MS2 and MS3 fragmentation, a hybridized HCD/CID approach was used. Each sample was analyzed using four injections, spanning the two aforementioned scan ranges, in both ionization modes. Data were analyzed using Thermo Scientific LipidSearch software (version 5.0) and in-house R scripts.

### Immunoprecipitation (IP)

The cell lysates were prepared in IP lysis buffer (Thermos Scientific, 87787) with protease inhibitor cocktails (Roche, 11836153001), followed by incubation on ice for 1 h. Lysates were centrifuged at 12,000 × *g* for 15 min at 4 °C. The clarified supernatants were incubated with the specific primary antibody or control IgG overnight at 4 °C. Dynabeads Protein G (Thermo Fisher Scientific, 10009D) was added to the lysates, followed by incubation for 4 h at 4 °C. The beads were washed with washing buffer (20 mM Tris-HCl [pH 7.5], 0.4 M NaCl, 1 mM EDTA, 0.1% Nonidet P-40, and 1× protease and phosphatase inhibitor cocktail) and resuspended with 1× Laemmli sample buffer, followed by Western blot analysis. The antibodies employed included anti-c-Myc (CST, 18583, 1:100), anti-CHD1 (Bethyl, A301-218A, 1:100), and normal rabbit IgG (Sigma, 12–370, 1:500). Detailed information on antibodies is listed in Supplementary Data 3.

### ChIP-sequencing

ChIP assays were performed using the SimpleChIP® Plus Enzymatic Chromatin IP Kit (Cell Signaling Technology, #9005). Briefly, cells were fixed with formaldehyde (1% final volume concentration) for 10 min at room temperature. Fixation was stopped by adding glycine and incubating for 5 min at room temperature. Chromatin was digested, and 10 μg of chromatin was incubated overnight at 4 °C with antibodies against CHD1 (1:100, 4351S, Cell Signaling Technology) or SP1 (1:50, 9389S, Cell Signaling Technology). Antibody–protein complexes were captured using ChIP-Grade Protein G Magnetic Beads and washed according to the manufacturer’s instructions. For ChIP-seq, immunoprecipitated chromatin and corresponding input DNA were reverse cross-linked and purified.

ChIP-Seq libraries were prepared by MD Anderson Advanced Technology Genomics Core (ATGC) Core facility using the KAPA Hyper Prep Kit, following the manufacturer’s protocol. The ChIP-Seq libraries, along with the corresponding input libraries, were sequenced using a 2 × 100 nt bases paired-end on an Illumina NovaSeq X instrument. ***Mapping***: Adapter sequences were removed from the 3’ ends of reads using Trim Galore (version 0.6.7) and cutadapt (version 4.1). The reads were then mapped to the human genome (hg38) using Bowtie (version 1.1.2) with the following parameters: “–allow-contain –maxins 2000 -v 2 -m 1 –best –strata”. To avoid PCR bias, only one copy of multiple fragments mapped to the same genomic position was retained for further analysis. Fragments longer than 500 bp (less than 1% of the total) were excluded from subsequent analysis. ***Signal track***: each fragment was resized to a length of 151 bp, spanning from −75 to +75 bp around its midpoint. The count of fragments covering each genomic position was normalized to 10 M total number of fragments. These values were then averaged over a 10 bp resolution. The resulting averaged values were displayed using UCSC genome browser^60^. ***Peak calling***: For each factor, fragment counts were downsampled to the same depth across samples to enable fair comparisons. Peaks were identified using MACS (version 1.4.2) by comparing against the corresponding input sample. A window size of 300 bp was used, and a p-value cutoff of 1e-5 was applied. The peaks that overlapped with ENCODE blacklisted regions were removed. ***Genomic distribution of peaks***: Each peak was assigned to the gene (from GENCODE Release 48^61^ basic gene annotation) with the closest transcription start site (TSS). Peaks were then classified as promoter or non-promoter, with promoters defined as regions spanning −5 to +1 kb relative to the gene TSS. The samples have been downsized to the same sequencing depth before peak calling for fair comparison

### Chromatin immunoprecipitation (ChIP)-qPCR

ChIP assays were performed as described above. Antibodies included normal IgG (Sigma, 12–370, 1:500), anti-CHD1 (Bethyl Laboratories, A301–218 A, 1:100), and anti-c-Myc (CST, 18583, 1:100). ChIP-enriched DNA was quantified by qPCR, and values were normalized to input controls. Data from triplicate measurements were analyzed and visualized using GraphPad Prism version 10. Primer sequences used for ChIP-qPCR are listed in Supplementary Data 3.

### Dual-luciferase reporter assay

A ~ 2 kb region spanning the ACSL4 promoter (−0.5 to +1.5 kb relative to the transcription start site) was cloned into the pGL4 vector and sequence-verified (Genewiz). Cells were co-transfected with the pGL4-ACSL4-promoter construct or empty pGL4 vector, along with the Renilla luciferase control plasmid, using Lipofectamine 2000 (Thermo Fisher Scientific, 11668019). Cells were co-transfected with siRNAs targeting SP1, c-Myc, or a non-targeting control. After 48 h, cells were lysed, and luciferase activity was measured using the Dual-Luciferase Reporter Assay System (Promega, E1910), following the manufacturer’s protocol. Firefly and Renilla luciferase signals were measured using a Synergy 2 Multi-Mode Microplate Reader (BioTek). Luciferase activity was normalized to the signal from cells co-transfected with pGL4-vector. All experiments were performed in triplicate, and statistical analyses were conducted using GraphPad Prism version 10.

### Xenograft studies

For xenograft experiments, 2 × 10^6^ LNCaP wild-type or SPOP-mutated cells with or without CHD1 knockout were suspended in PBS:Matrigel (1:1) and subcutaneously injected into both flanks of 6-week-old male SCID mice (CB17/Icr-Prkdcscid/IcrIcoCrl, Charles River). Once tumors reached ~50 mm³, tumor-bearing mice were randomized and treated with JKE-1674 (MedChemExpress, HY-138153, 15 mg/kg; oral gavage) or vehicle control every other day for three weeks. Tumor volumes were measured twice weekly using digital calipers and calculated using the formula: (width² × length)/2. To determine whether the antitumor effect was mediated by ferroptosis, additional cohorts bearing SPOP-mutant LNCaP xenografts were treated with vehicle, JKE-1674 (25 mg/kg, every other day), or liproxstatin-1 (Cayman Chemical, 17730; 10 mg/kg/day, intraperitoneal injection) with the same experimental design described above. For combination therapy studies, 2 × 10^6^ SPOP-mutated/CHD1-deleted LNCaP cells were subcutaneously injected into both flanks of 6-week-old male SCID mice. After tumors reached ~100 mm^3^, tumor-bearing mice were randomized and orally administered JKE-1674 (15 mg/kg; every other day) and atorvastatin (Selleckchem, S2077; 20 mg/kg; every day), as single agents or in combination, for three weeks. Tumor growth was monitored biweekly, and volumes were calculated as described. At the study endpoint, tumors were excised for histopathological analysis.

### Experimental GEMMs and drug treatment

GEMMs of PB-Cre; Pten^L/L^ (P), PB-Cre; Pten^L/L^; CHD1^L/L^ (PC), PB-Cre; Pten^L/L^; SPOP^F133V^ (PSp) and PB-Cre; Pten^L/L^; SPOP^F133V^; CHD1^L/L^ (PSpC) were previously reported^34–36^. All transgenic mice used in this study were male and maintained on >90% C57BL/6J background. Ten-month-old P, PSp, and PSpC mice bearing spontaneous prostate tumors were orally administered JKE-1674 (15 mg/kg, every other day, oral gavage) for 40 days. For combination treatment, ten-month-old PSpC mice bearing spontaneous prostate tumors were orally administered JKE-1674 (15 mg/kg, every other day) and atorvastatin (20 mg/kg, everyday) for four weeks. Tumor progression was monitored biweekly using a 7 T MRI (Bruker Biospin MRI, Billerica, MA) before and after drug treatment. Tumor volume was qualified using ImageJ (Fiji) software (version 2.1.0/1.53c, National Institutes of Health, Bethesda, MD). Prostate tumors were collected at the endpoint for histopathology analysis. All animal procedures were conducted under MD Anderson Institutional Animal Care and Use Committee (IACUC) protocols.

### Statistics and reproducibility

Sample sizes were not predetermined using statistical methods but were similar to those reported in our previous studies^36^. Mice of matched age, tumor samples, and cell line cultures were randomly assigned to experimental groups. No mice or data were excluded from the analyses. All experiments were performed and analyzed blinded to the genotype, group, and treatment whenever possible. Data collection was conducted in a blinded manner, with group allocation determined afterward. In animal sample collection, comparable samples were obtained without immediate analysis, sample analyses in follow-up experiments were conducted in a blinded manner. All experiments were independently performed at least three times with similar results. Data distribution was assumed to be normal, but this was not formally tested. Unless otherwise stated, all statistical analyses were carried out using GraphPad Prism Version 10. An unpaired two-tailed Student’s *T*-test was performed to analyze data between the two groups. One-way or two-way ANOVA with Tukey’s or Dunnett’s multiple comparison tests was performed to analyze data from three or more groups, as indicated in the figure legends. Unless otherwise stated, numerical data are presented as the mean ± standard deviation of at least three biological replicates.

### Reporting summary

Further information on research design is available in the Nature Portfolio Reporting Summary linked to this article.

## Supplementary information

Supplementary Information

Description of Addtional Supplementary Files

Supplementary Data 1

Supplementary Data 2

Supplementary Data 3

Reporting Summary

Transparent Peer Review file

## Source data

Source Data

## Data Availability

ChIP-sequencing data supporting this study’s findings have been deposited in the Gene Expression Omnibus (GEO) under accession GSE317501. The lipidomic profiling data are available in Supplementary Data 1 and have been deposited in the Zenodo database under accession 19714093. Human pan-cancer and prostate cancer genomic and bulk RNA-seq data were derived from the TCGA Research Network: http://cancergenome.nih.gov/. Source data for Western Blot images and Numerical results in all Figures and Supplementary Figures have been provided as Source Data files. All the other data are available within the article and its Supplementary Information. Source data are provided with this paper.

## Code availability

The custom codes used for lipidomic profiling data analysis have been deposited in the public repository: https://github.com/MD-Anderson-Bioinformatics/xPEDITE.

## References

[1] Siegel, R. L., Giaquinto, A. N. & Jemal, A. Cancer statistics, 2024. *CA Cancer J. Clin.* **74**, 12–49 (2024). 38230766 10.3322/caac.21820

[2] Robinson, D. et al. Integrative clinical genomics of advanced prostate cancer. *Cell* **161**, 1215–1228 (2015). 26000489 10.1016/j.cell.2015.05.001 PMC4484602

[3] Hoadley, K. A. et al. Cell-of-origin patterns dominate the molecular classification of 10,000 tumors from 33 types of cancer. *Cell* **173**, 291–304 e296 (2018). 29625048 10.1016/j.cell.2018.03.022 PMC5957518

[4] Armenia, J. et al. The long tail of oncogenic drivers in prostate cancer. *Nat. Genet.* **50**, 645–651 (2018). 29610475 10.1038/s41588-018-0078-z PMC6107367

[5] The Cancer Genome Atlas Research Network The molecular taxonomy of primary prostate cancer. *Cell* **163**, 1011–1025 (2015). 26544944 10.1016/j.cell.2015.10.025 PMC4695400

[6] Berger, M. F. et al. The genomic complexity of primary human prostate cancer. *Nature* **470**, 214–220 (2011). 21307934 10.1038/nature09744 PMC3075885

[7] Barbieri, C. E. et al. Exome sequencing identifies recurrent SPOP, FOXA1 and MED12 mutations in prostate cancer. *Nat. Genet.* **44**, 685–689 (2012). 22610119 10.1038/ng.2279 PMC3673022

[8] Boysen, G. et al. SPOP mutation leads to genomic instability in prostate cancer. *Elife* **4**, e09207 (2015). 26374986 10.7554/eLife.09207 PMC4621745

[9] Robinson, D. et al. Integrative clinical genomics of advanced prostate cancer. *Cell* **162**, 454 (2015). 28843286 10.1016/j.cell.2015.06.053

[10] Geng, C. et al. SPOP regulates prostate epithelial cell proliferation and promotes ubiquitination and turnover of c-MYC oncoprotein. *Oncogene* **36**, 4767–4777 (2017). 28414305 10.1038/onc.2017.80 PMC5887163

[11] Geng, C. et al. Androgen receptor is the key transcriptional mediator of the tumor suppressor SPOP in prostate cancer. *Cancer Res.* **74**, 5631–5643 (2014). 25274033 10.1158/0008-5472.CAN-14-0476 PMC4209379

[12] An, J., Wang, C., Deng, Y., Yu, L. & Huang, H. Destruction of full-length androgen receptor by wild-type SPOP, but not prostate-cancer-associated mutants. *Cell Rep.* **6**, 657–669 (2014). 24508459 10.1016/j.celrep.2014.01.013 PMC4361392

[13] Gan, W. et al. SPOP promotes ubiquitination and degradation of the ERG oncoprotein to suppress prostate cancer progression. *Mol. Cell* **59**, 917–930 (2015). 26344095 10.1016/j.molcel.2015.07.026 PMC4575912

[14] An, J. et al. Truncated ERG oncoproteins from TMPRSS2-ERG fusions are resistant to SPOP-mediated proteasome degradation. *Mol. Cell* **59**, 904–916 (2015). 26344096 10.1016/j.molcel.2015.07.025

[15] Groner, A. C. et al. TRIM24 is an oncogenic transcriptional activator in prostate cancer. *Cancer Cell* **29**, 846–858 (2016). 27238081 10.1016/j.ccell.2016.04.012 PMC5124371

[16] Li, C. et al. Tumor-suppressor role for the SPOP ubiquitin ligase in signal-dependent proteolysis of the oncogenic co-activator SRC-3/AIB1. *Oncogene* **30**, 4350–4364 (2011). 21577200 10.1038/onc.2011.151 PMC3158261

[17] Geng, C. et al. Prostate cancer-associated mutations in speckle-type POZ protein (SPOP) regulate steroid receptor coactivator 3 protein turnover. *Proc. Natl. Acad. Sci. USA*. **110**, 6997–7002 (2013). 23559371 10.1073/pnas.1304502110 PMC3637757

[18] Zhuang, M. et al. Structures of SPOP-substrate complexes: insights into molecular architectures of BTB-Cul3 ubiquitin ligases. *Mol. Cell* **36**, 39–50 (2009). 19818708 10.1016/j.molcel.2009.09.022 PMC2847577

[19] Swami, U. et al. Association of SPOP mutations with outcomes in men with de novo metastatic castration-sensitive prostate cancer. *Eur. Urol.* **78**, 652–656 (2020). 32624276 10.1016/j.eururo.2020.06.033 PMC7572891

[20] Nakazawa, M. et al. Clinical and genomic features of SPOP-mutant prostate cancer. *Prostate* **82**, 260–268 (2022). 34783071 10.1002/pros.24269 PMC8688331

[21] Stopsack, K. H. et al. Oncogenic genomic alterations, clinical phenotypes, and outcomes in metastatic castration-sensitive prostate cancer. *Clin. Cancer Res.* **26**, 3230–3238 (2020). 32220891 10.1158/1078-0432.CCR-20-0168 PMC7334067

[22] Swami, U. et al. SPOP mutations as a predictive biomarker for androgen receptor axis-targeted therapy in de novo metastatic castration-sensitive prostate cancer. *Clin. Cancer Res.* **28**, 4917–4925 (2022). 36088616 10.1158/1078-0432.CCR-22-2228

[23] Boysen, G. et al. SPOP-mutated/CHD1-deleted lethal prostate cancer and abiraterone sensitivity. *Clin. Cancer Res.* **24**, 5585–5593 (2018). 30068710 10.1158/1078-0432.CCR-18-0937 PMC6830304

[24] Liu, D. et al. Tumor subtype defines distinct pathways of molecular and clinical progression in primary prostate cancer. *J. Clin. Invest.* **131**, e147878 (2021). 33998599 10.1172/JCI147878 PMC8121518

[25] Stockdale, C., Flaus, A., Ferreira, H. & Owen-Hughes, T. Analysis of nucleosome repositioning by yeast ISWI and Chd1 chromatin remodeling complexes. *J. Biol. Chem.* **281**, 16279–16288 (2006). 16606615 10.1074/jbc.M600682200 PMC1764501

[26] Lusser, A., Urwin, D. L. & Kadonaga, J. T. Distinct activities of CHD1 and ACF in ATP-dependent chromatin assembly. *Nat. Struct. Mol. Biol.* **12**, 160–166 (2005). 15643425 10.1038/nsmb884

[27] Li, H., Gigi, L. & Zhao, D. CHD1, a multifaceted epigenetic remodeler in prostate cancer. *Front. Oncol.* **13**, 1123362 (2023). 36776288 10.3389/fonc.2023.1123362 PMC9909554

[28] Burkhardt, L. et al. CHD1 is a 5q21 tumor suppressor required for ERG rearrangement in prostate cancer. *Cancer Res.* **73**, 2795–2805 (2013). 23492366 10.1158/0008-5472.CAN-12-1342

[29] Augello, M. A. et al. CHD1 loss alters AR binding at lineage-specific enhancers and modulates distinct transcriptional programs to drive prostate tumorigenesis. *Cancer Cell* **35**, 603–617 e608 (2019). 30930119 10.1016/j.ccell.2019.03.001 PMC6467783

[30] Zhang, Z. et al. Loss of CHD1 promotes heterogeneous mechanisms of resistance to AR-targeted therapy via chromatin dysregulation. *Cancer Cell* **37**, 584–598 e511 (2020). 32220301 10.1016/j.ccell.2020.03.001 PMC7292228

[31] Metzger, E. et al. Assembly of methylated KDM1A and CHD1 drives androgen receptor-dependent transcription and translocation. *Nat. Struct. Mol. Biol.* **23**, 132–139 (2016). 26751641 10.1038/nsmb.3153

[32] Rodrigues, L. U. et al. Coordinate loss of MAP3K7 and CHD1 promotes aggressive prostate cancer. *Cancer Res.* **75**, 1021–1034 (2015). 25770290 10.1158/0008-5472.CAN-14-1596 PMC4531265

[33] Jillson, L. K. et al. MAP3K7 loss drives enhanced androgen signaling and independently confers risk of recurrence in prostate cancer with joint loss of CHD1. *Mol. Cancer Res.* **19**, 1123–1136 (2021). 33846123 10.1158/1541-7786.MCR-20-0913 PMC8254790

[34] Zhao, D. et al. Synthetic essentiality of chromatin remodelling factor CHD1 in PTEN-deficient cancer. *Nature* **542**, 484–488 (2017). 28166537 10.1038/nature21357 PMC5448706

[35] Zhao, D. et al. Chromatin regulator CHD1 remodels the immunosuppressive tumor microenvironment in PTEN-deficient prostate cancer. *Cancer Discov.* **10**, 1374–1387 (2020). 32385075 10.1158/2159-8290.CD-19-1352 PMC7483306

[36] Chen, F. et al. CHD1 loss reprograms SREBP2-driven cholesterol synthesis to fuel androgen-responsive growth and castration resistance in SPOP-mutated prostate tumors. *Nat. Cancer* **6**, 854–873 (2025). 10.1038/s43018-025-00952-z PMC12276840 40360905

[37] Dixon, S. J. et al. Ferroptosis: an iron-dependent form of nonapoptotic cell death. *Cell* **149**, 1060–1072 (2012). 22632970 10.1016/j.cell.2012.03.042 PMC3367386

[38] Jiang, X., Stockwell, B. R. & Conrad, M. Ferroptosis: mechanisms, biology and role in disease. *Nat. Rev. Mol. Cell Biol.* **22**, 266–282 (2021). 33495651 10.1038/s41580-020-00324-8 PMC8142022

[39] Doll, S. et al. ACSL4 dictates ferroptosis sensitivity by shaping cellular lipid composition. *Nat. Chem. Biol.* **13**, 91–98 (2017). 27842070 10.1038/nchembio.2239 PMC5610546

[40] Lei, G., Zhuang, L. & Gan, B. Targeting ferroptosis as a vulnerability in cancer. *Nat. Rev. Cancer* **22**, 381–396 (2022). 35338310 10.1038/s41568-022-00459-0 PMC10243716

[41] Wang, M. E. et al. RB1-deficient prostate tumor growth and metastasis are vulnerable to ferroptosis induction via the E2F/ACSL4 axis. *J. Clin. Invest.* **133**, e166647 (2023). 36928314 10.1172/JCI166647 PMC10178842

[42] Lei, G. et al. BRCA1-mediated dual regulation of ferroptosis exposes a vulnerability to GPX4 and PARP co-inhibition in BRCA1-deficient cancers. *Cancer Discov.* **14**, 1476–1495 (2024). 38552003 10.1158/2159-8290.CD-23-1220 PMC11296921

[43] Cao, P. H. A. et al. Unlocking ferroptosis in prostate cancer - the road to novel therapies and imaging markers. *Nat. Rev. Urol.* **21**, 615–637 (2024). 38627553 10.1038/s41585-024-00869-9 PMC12067944

[44] Liang, D. et al. Ferroptosis surveillance independent of GPX4 and differentially regulated by sex hormones. *Cell* **186**, 2748–2764 e2722 (2023). 37267948 10.1016/j.cell.2023.05.003 PMC10330611

[45] Blattner, M. et al. SPOP mutation drives prostate tumorigenesis in vivo through coordinate regulation of PI3K/mTOR and AR signaling. *Cancer Cell* **31**, 436–451 (2017). 28292441 10.1016/j.ccell.2017.02.004 PMC5384998

[46] Sun, N. et al. Oncogenic RTKs sensitize cancer cells to ferroptosis via c-Myc mediated upregulation of ACSL4. *Cell Death Dis.* **15**, 861 (2024). 39604370 10.1038/s41419-024-07254-9 PMC11603294

[47] Anselmino, N. et al. Integrative molecular analyses of the MD Anderson prostate cancer patient-derived xenograft (MDA PCa PDX) series. *Clin. Cancer Res.* **30**, 2272–2285 (2024). 38488813 10.1158/1078-0432.CCR-23-2438 PMC11094415

[48] Eaton, J. K. et al. Selective covalent targeting of GPX4 using masked nitrile-oxide electrophiles. *Nat. Chem. Biol.* **16**, 497–506 (2020). 32231343 10.1038/s41589-020-0501-5 PMC7251976

[49] Luirink, I. K. et al. 20-year follow-up of statins in children with familial hypercholesterolemia. *N. Engl. J. Med.* **381**, 1547–1556 (2019). 31618540 10.1056/NEJMoa1816454

[50] Cao, S. et al. KLF11-mediated repression antagonizes Sp1/sterol-responsive element-binding protein-induced transcriptional activation of caveolin-1 in response to cholesterol signaling. *J. Biol. Chem.* **280**, 1901–1910 (2005). 15531587 10.1074/jbc.M407941200

[51] Li, Y. et al. Ischemia-induced ACSL4 activation contributes to ferroptosis-mediated tissue injury in intestinal ischemia/reperfusion. *Cell Death Differ.* **26**, 2284–2299 (2019). 30737476 10.1038/s41418-019-0299-4 PMC6889315

[52] Viswanathan, V. S. et al. Dependency of a therapy-resistant state of cancer cells on a lipid peroxidase pathway. *Nature* **547**, 453–457 (2017). 28678785 10.1038/nature23007 PMC5667900

[53] Friedmann Angeli, J. P. et al. Inactivation of the ferroptosis regulator Gpx4 triggers acute renal failure in mice. *Nat. Cell Biol.* **16**, 1180–1191 (2014). 25402683 10.1038/ncb3064 PMC4894846

[54] Yang, W. S. et al. Regulation of ferroptotic cancer cell death by GPX4. *Cell* **156**, 317–331 (2014). 24439385 10.1016/j.cell.2013.12.010 PMC4076414

[55] Zhang, C. et al. JMJD6 rewires ATF4-dependent glutathione metabolism to confer ferroptosis resistance in SPOP-mutated prostate cancer. *Cancer Res.* **85**, 1496–1513 (2025). 39903850 10.1158/0008-5472.CAN-23-2796

[56] Magtanong, L. et al. Context-dependent regulation of ferroptosis sensitivity. *Cell Chem. Biol.* **29**, 1409–1418 e1406 (2022). 35809566 10.1016/j.chembiol.2022.06.004 PMC9481678

[57] Drost, J. et al. Organoid culture systems for prostate epithelial and cancer tissue. *Nat. Protoc.* **11**, 347–358 (2016). 26797458 10.1038/nprot.2016.006 PMC4793718

[58] Li, H. et al. CHD1 promotes sensitivity to aurora kinase inhibitors by suppressing interaction of AURKA with its coactivator TPX2. *Cancer Res.* **82**, 3088–3101 (2022). 35771632 10.1158/0008-5472.CAN-22-0631 PMC9444962

[59] Bankhead, P. et al. QuPath: open source software for digital pathology image analysis. *Sci. Rep.* **7**, 16878 (2017). 29203879 10.1038/s41598-017-17204-5 PMC5715110

[60] Casper, J. et al. The UCSC Genome Browser database: 2026 update. *Nucleic Acids Res.* **54**, D1331–D1335 (2026). 41251146 10.1093/nar/gkaf1250 PMC12807699

[61] Mudge, J. M. et al. GENCODE 2025: reference gene annotation for human and mouse. *Nucleic Acids Res.* **53**, D966–D975 (2025). 39565199 10.1093/nar/gkae1078 PMC11701607

