# Supplementary material for: Divergent roles of SPOP and CHD1 in ACSL4 regulation reveal context-dependent vulnerabilities for targeting ferroptosis
Source: Nat Commun. 2026 Jun 30;17:8385. doi: 10.1038/s41467-026-75010-y (PMC13473601; doi:10.1038/s41467-026-75010-y)
Supplement: Supplementary file 1 [file 41467_2026_75010_MOESM1_ESM.pdf]

Supplementary Information

Divergent Roles of SPOP and CHD1 in ACSL4 Regulation Reveal Context-dependent Vulnerabilities for Targeting Ferroptosis

Feiyu Chen[1#], Qidong Li[1#], Qianlin Gu[1], Javier Leo[1,2], Xin Liang[1], Naayaa Mehta[1], Estefania Labanca[3], Peter Shepherd[3], Iqbal Mahmud[4], Yin Wang[1], Francisco R. Saenz[5], Maya M. Phillips[1], Wei Shi[1], Chenling Meng[1], Jie Zhang[1], Daniel E. Frigo[6,3], Yue Lu[7], Boyi Gan[1*], Di Zhao[1*]

The PDF file includes:

- Supplementary figures 1-8

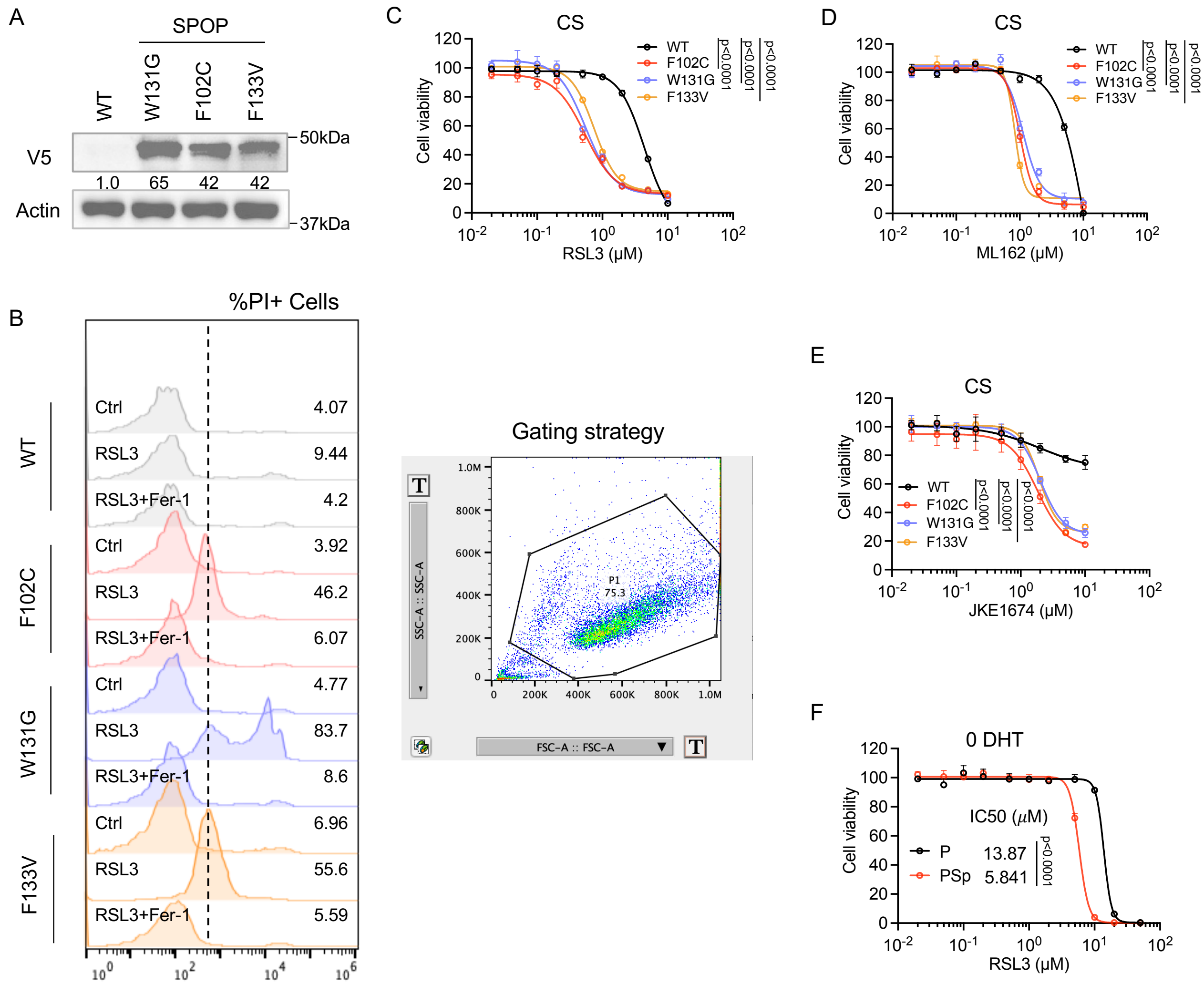

Figure S1. SPOP mutations sensitize PCa cells to ferroptosis inducers targeting GPX4 .

A, Western blot analysis of V5-tagged SPOP mutants (F102C, W131G, F133V) in LNCaP cells. Relative density ratios of indicated proteins/actin are shown. The samples derive from the same experiment but different gels for V5 and Actin were processed in parallel. **B,** PI staining of WT or SPOP-mutated (F102C, W131G, F133V) LNCaP cells treated with or without RSL3 for 24 hr, in the presence of ferrostatin-1 (Fer-1, 10 μM) (left); The gating strategy is shown on the right. **C-E,** Dose-response curves of WT or SPOP^{W131G} LNCaP cells cultured in charcoal-stripped (CS) medium and treated with different doses of RSL3 (**C**), ML162 (**D**), and JKE-1674 (**E**) for 24 hr. **F,** Dose-response and IC50s of RSL3 in P and PSp tumor organoids cultured in media without DHT. Data are presented as mean ± standard deviation, n = 3 biologically independent repeats. *P* values determined by two-way ANOVA (C-F) analysis. Source data are provided as a Source Data file.

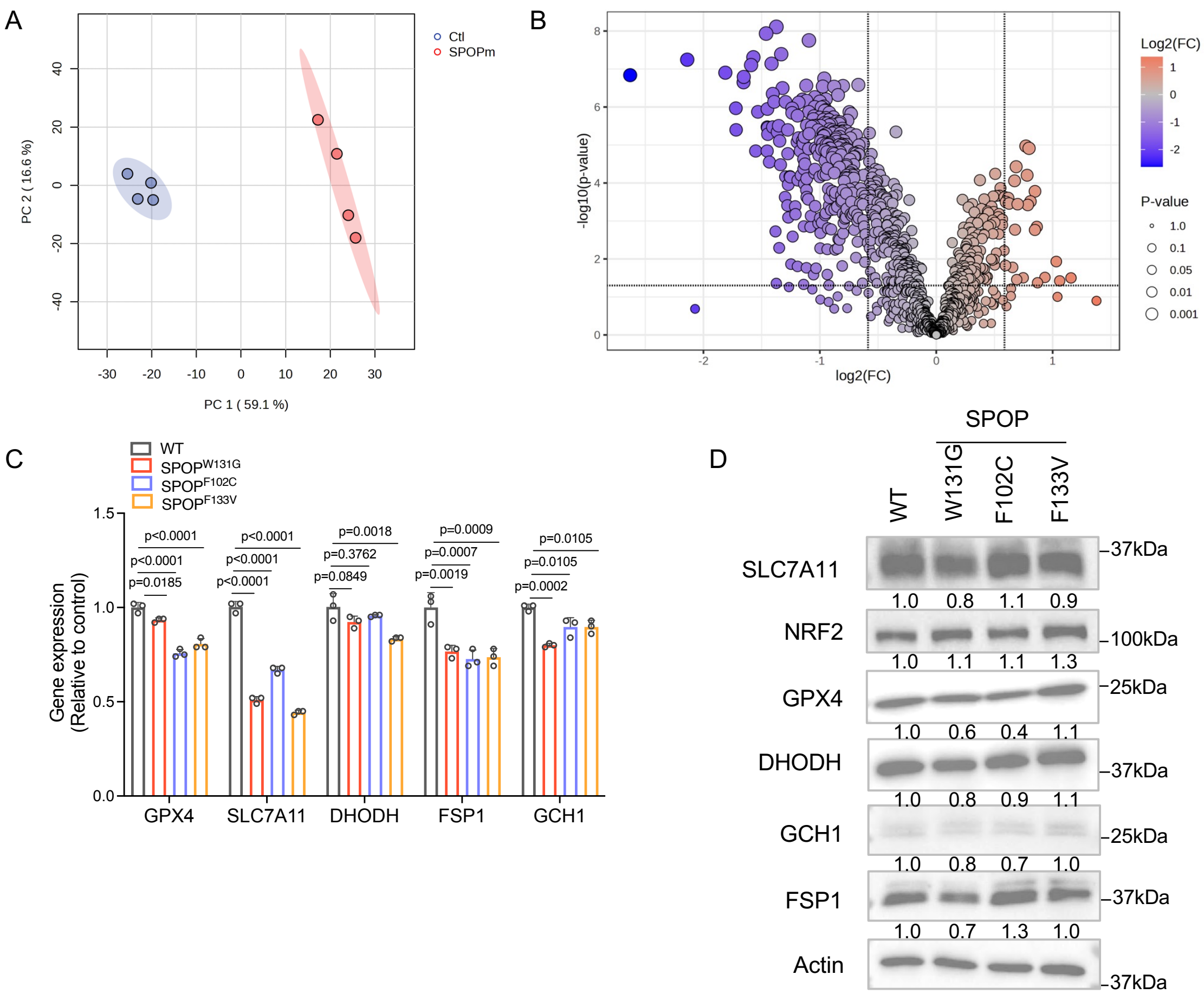

Figure S2. ACSL4 mediates ferroptosis sensitivity driven by SPOP mutations

A, Lipidomic profiling was performed in control (blue) and SPOP^{W131G} (red) LNCaP cells with 4 replicates using LC–MS/MS. Principal component analysis (PCA) of gene expression profiles showing clear separation between control (blue) and SPOP^{W131G} (red) LNCaP cells. The percentage of variance explained by each principal component is indicated on the axes. **B,** Volcano plot of all lipid species detected in control and SPOP^{W131G} samples. Vertical and horizontal dashed lines denote the cutoffs used for fold change and statistical significance, respectively. P values and Log2(fold-change) are shown. **C-D,** mRNA (**C**) and protein (**D**) levels of indicated ferroptosis regulators in WT or SPOP-mutated (F102C, W131G, F133V) LNCaP cells. Relative density ratios of indicated proteins/actin are shown. The samples derive from the same experiment but different gels for SLC7A11, NRF2, another for GPX4, another for DHODH, another for GCH1, another for FSP1, and another for Actin were processed in parallel. Data are presented as mean ± standard deviation, n = 3 biologically independent repeats. *P* values determined by one-way ANOVA (C) analysis. Source Data are provided as a Source Data file.

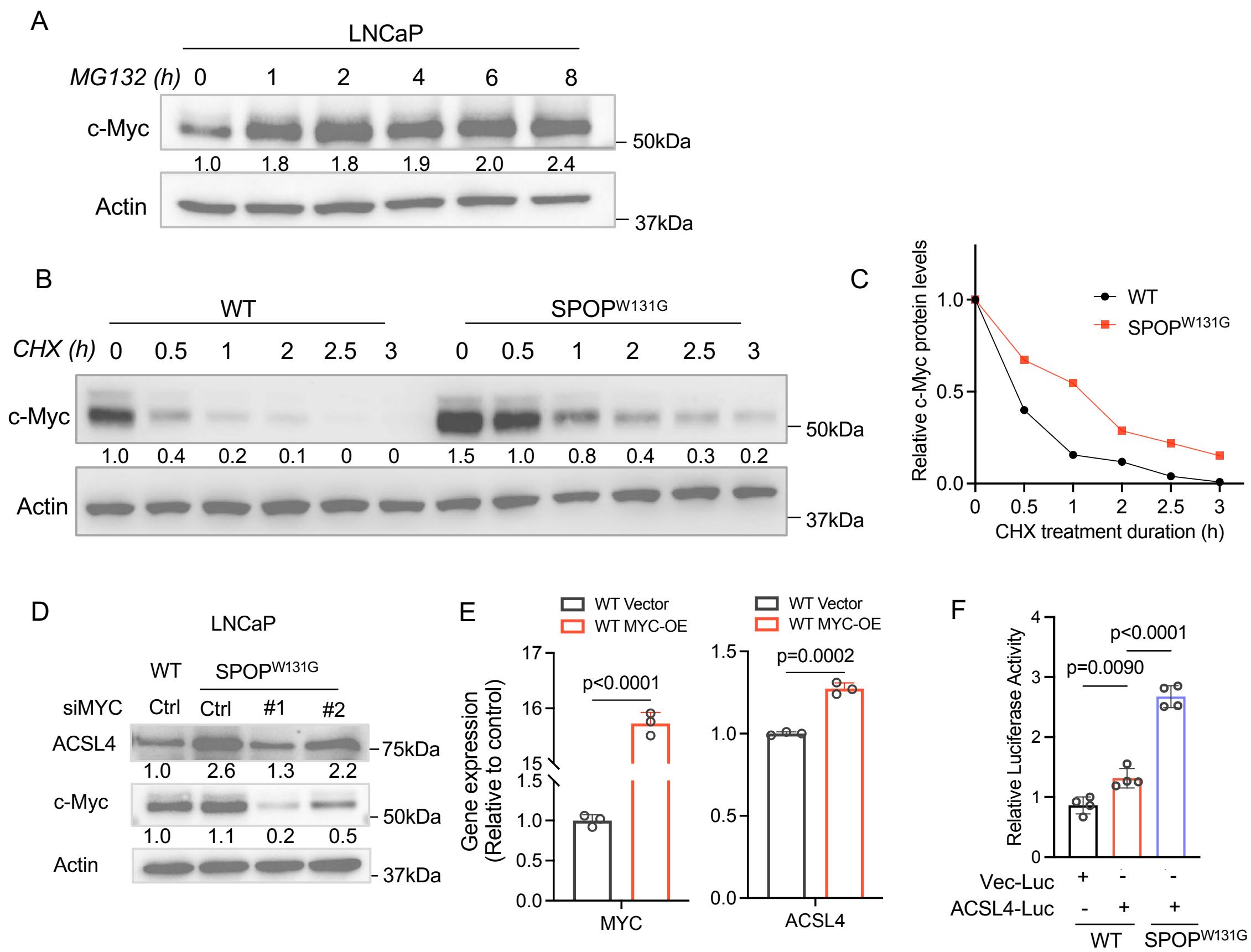

Figure S3. C-Myc stabilization links SPOP mutations to ACSL4-mediated ferroptosis

A, Western blot analysis of c-Myc in LNCaP cells after treatment with MG132 (20 μM) for the indicated times (0, 1, 2, 4, 6, and 8 hr). Relative density ratios of indicated proteins/actin are shown. **B-C,** Western blot analysis and quantification of c-Myc protein levels in WT or SPOP^{W131G} LNCaP cells after treatment with cycloheximide (CHX, 100 μg/ml) for the indicated times (0, 0.5, 1, 2, 2.5, and 3 hr). Relative density ratios of indicated proteins/actin are shown. The samples derive from the same experiment but different gels for c-Myc and Actin were processed in parallel. **D,** MYC was knocked down in WT and SPOP^{W131G} LNCaP cells using two individual siRNAs, followed by determining c-Myc and ACSL4 protein expression using Western blot. Relative density ratios of indicated proteins/actin are shown. The samples derive from the same experiment but different gels for c-Myc, another for ACSL4, and another for Actin were processed in parallel. **E,** MYC was overexpressed (OE) in LNCaP cells, followed by qPCR analysis of MYC and ACSL4 mRNA levels. **F,** Luciferase reporter of the ACSL4 promoter was introduced into WT and SPOP^{W131G} LNCaP cells, followed by luciferase assays. n = 4. Data are presented as mean ± standard deviation, n = 3 biologically independent repeats unless otherwise stated. *P* values determined by unpaired two-tailed t test (E) or one-way ANOVA (F) analysis. Source data are provided as a Source Data file.

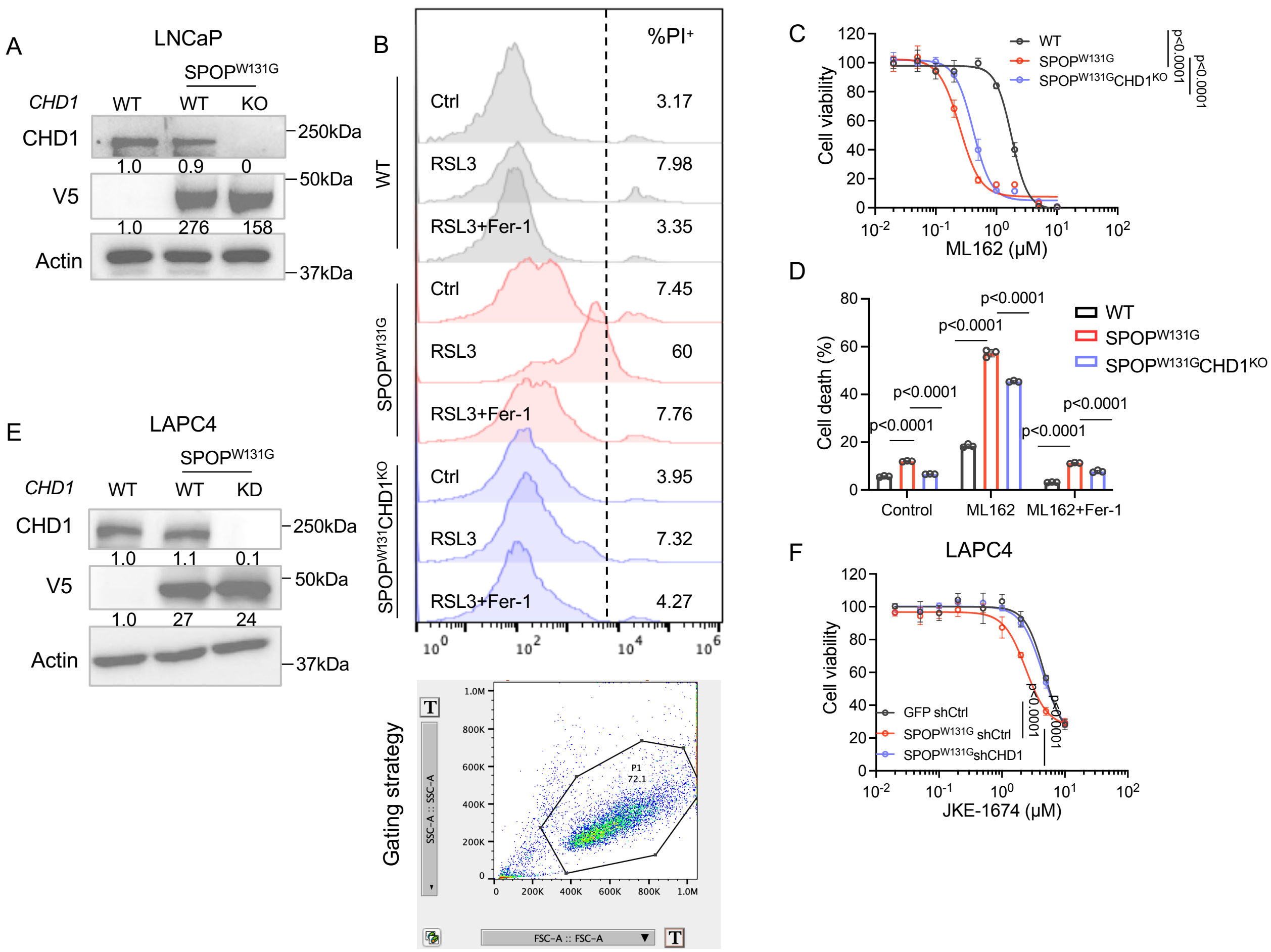

Figure S4. Loss of CHD1 confers ferroptosis resistance in SPOP-mutated PCa

A, Western blot assays of CHD1 and V5-tagged SPOP in WT and $SPOP^{W131G}$ LNCaP cells with or without CHD1 knockout (KO). Relative density ratios of indicated proteins/actin are shown. The samples derive from the same experiment but different gels for CHD1, Actin, another for V5 were processed in parallel. **B,** PI staining indicates cell death in WT and $SPOP^{W131G}$ LNCaP cells with or without CHD1 KO after treatment with RSL3 (1 μM, 24 hr), in the presence of ferrostatin-1 (Fer-1, 10 μM). The gating strategy is shown below. **C-D,** Dose-response curves (**C**) and cell death (**D**) of ML162 in WT and $SPOP^{W131G}$ LNCaP cells with or without CHD1 KO following 24 hr treatment. **E,** Western blot assays of WT and $SPOP^{W131G}$ LAPC4 cells with or without CHD1 knockdown (KD). Relative density ratios of indicated proteins/actin are shown. The samples derive from the same experiment but different gels for CHD1, Actin, another for V5 were processed in parallel. **F,** Dose-response curves of JKE-1674 in WT and $SPOP^{W131G}$ LAPC4 cells with or without CHD1 KD following 24 hr treatment. Data are presented as mean ± standard deviation, n = 3 biologically independent repeats. *P* values determined by two-way ANOVA (C,D,F) analysis. Source data are provided as a Source Data file.

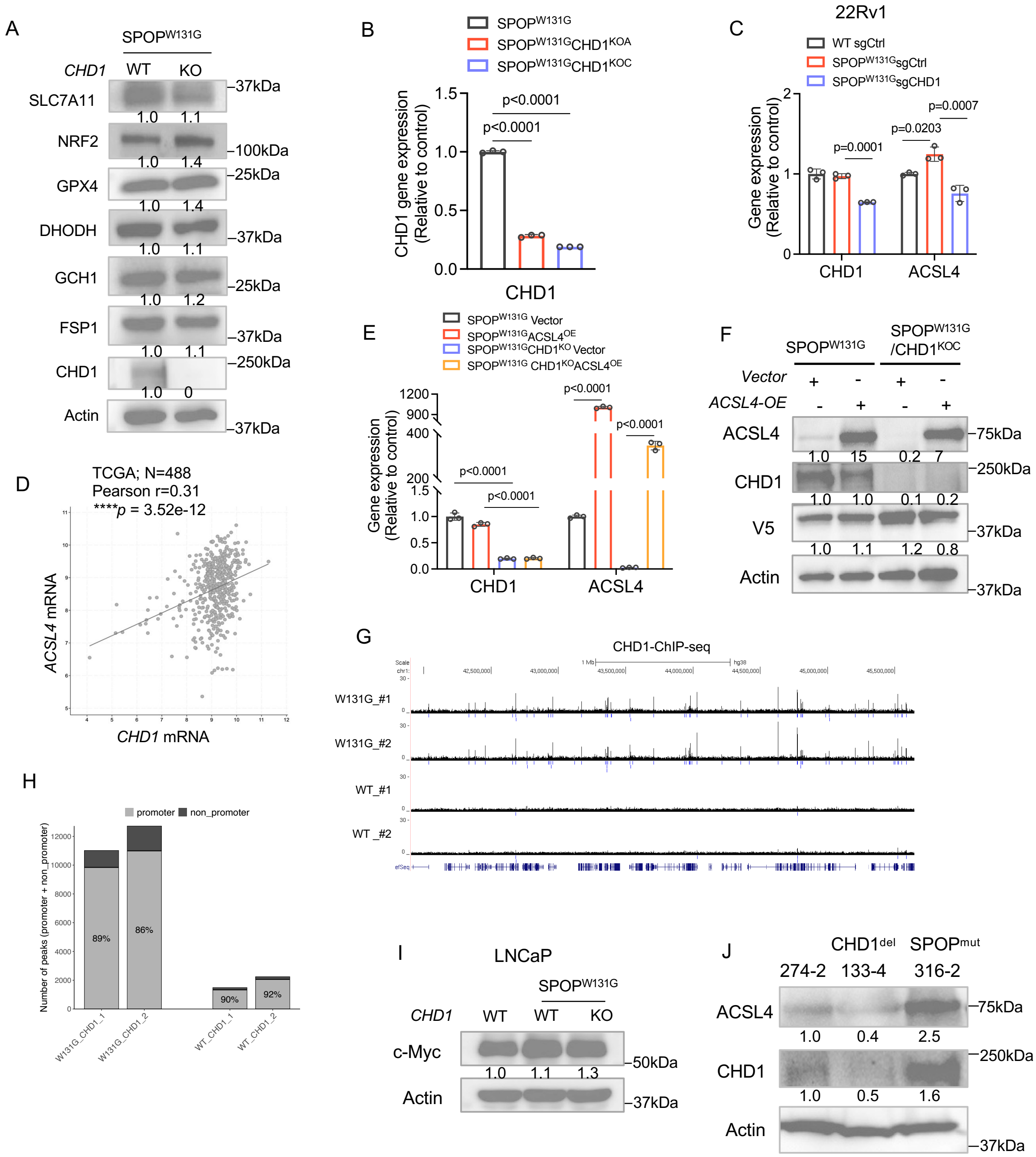

Figure S5. CHD1 is required for the transcriptional activation of ACSL4 by interacting with c-Myc

A, Western blot assays of ferroptosis regulators GPX4, SLC7A11, FSP1, DHODH, and GCH1 in SPOP^{W131G} LNCaP cells with or without CHD1 knockout. Relative density ratios of indicated proteins/actin are shown. The samples derive from the same experiment but different gels for SLC7A11, NRF2, another for GPX4, another for DHODH, another for GCH1, another for FSP1, and another for CHD1, Actin were processed in parallel. **B,** CHD1 was knocked out in SPOP^{W131G} LNCaP cells using two sgRNAs (KO_A and KO_C), followed by the determination of CHD1 mRNA levels by qPCR. **C,** mRNA levels of CHD1 and ACSL4 in WT and SPOP^{W131G} 22Rv1 cells with or without CHD1 KO. **D,** Scatter plots illustrating the correlations between CHD1 and ACSL4 mRNA levels in human PCa samples (TCGA; n=488). The Pearson correlation coefficient (r) and *P* value are reported. **E-F,** qPCR (**E**) and Western blot (**F**) assays show the mRNA and protein levels of CHD1 and ACSL4 in SPOP^{W131G} LNCaP cells upon CHD1 deletion and/or ACSL4 overexpression. **G-H,** ChIP-seq was performed in WT and SPOP^{W131G} LNCaP cells using IgG control or CHD1 antibodies. Representative landscape of CHD1 in four samples was created using USCS genome browser (**G**). The number of peaks on promoter or none-promoters of CHD1 in WT versus SPOP^{W131G} LNCaP cells are presented (**H**). **I,** c-Myc protein levels in WT and SPOP^{W131G} LNCaP cells with or without CHD1 KO. Relative density ratios of indicated proteins/actin are shown. The samples derive from the same experiment but different gels for CHD1 and Actin were processed in parallel. **J,** Western blot analyses of CHD1 and ACSL4 in PCa patient-derived xenograft (PDX) tumors: 274-2 (intact SPOP/CHD1), 133-4 (CHD1^{del}), and 316-2 (SPOP^{mut}). Relative density ratios of indicated proteins/actin are shown. The samples derive from the same experiment but different gels for ACSL4, Actin, another for CHD1 were processed in parallel. Data are presented as mean ± standard deviation, n = 3 biologically independent repeats. *P* values determined by one-way ANOVA (B,C,E) analysis. Source data are provided as a Source Data file.

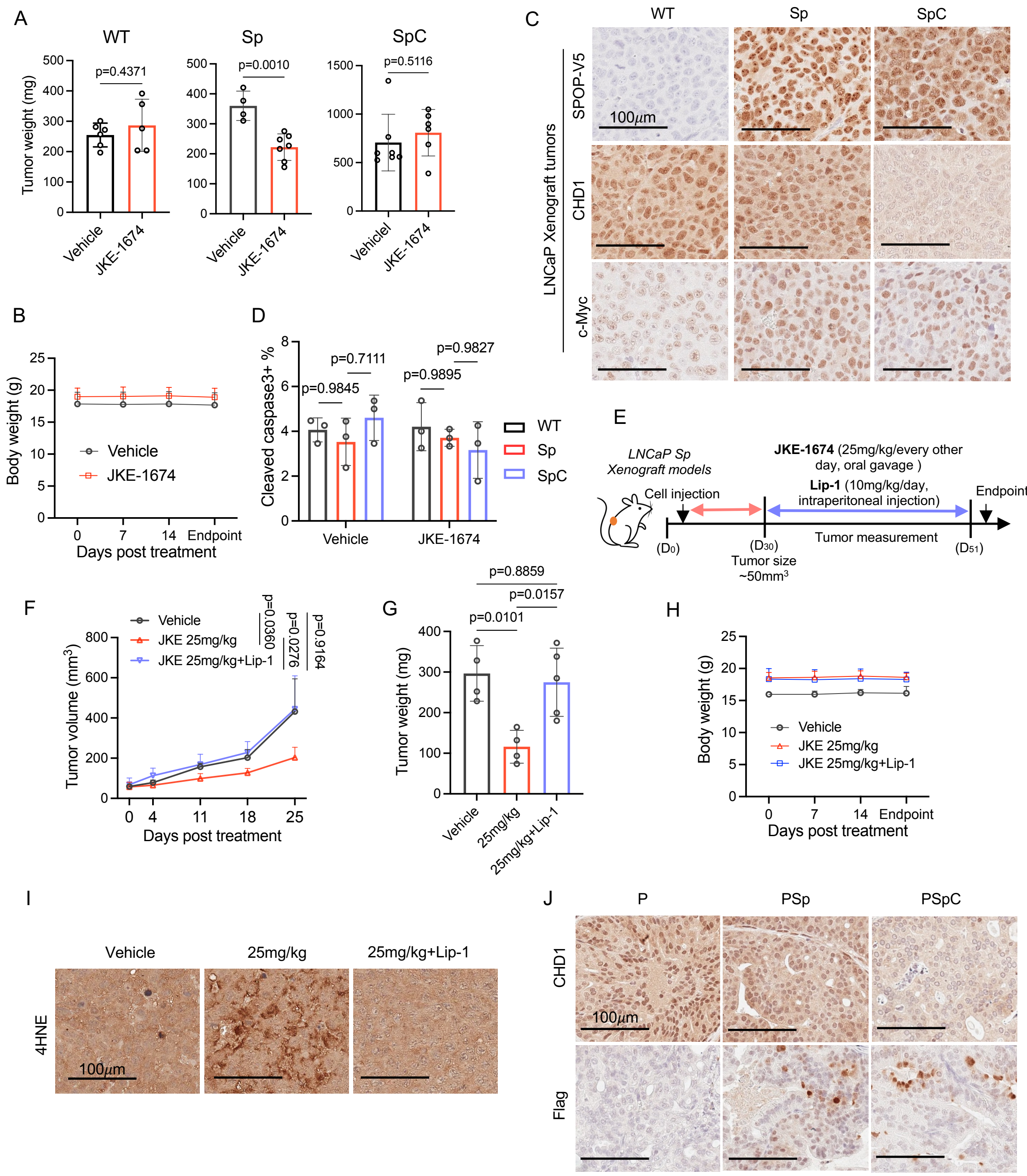

Figure S6. SPOP mutations promote, whereas CHD1 deletion reduces, the ferroptosis susceptibility in preclinical models of PCa

A, Tumor weight at the end point of wildtype (WT), SPOP^{W131G} (Sp), and SPOP^{W131G}/CHD1^{KO} (SpC) LNCaP xenograft tumors after JKE-1674 or vehicle treatment. **B,** Body weights of tumor-bearing mice over the treatment period. **C,** IHC staining of CHD1, V5-tagged SPOP^{W131G}, and c-Myc in WT, Sp, SpC xenograft tumors. Scale bar, 100 µm. **D,** Quantification of cleaved caspase-3 IHC staining in WT, Sp and SpC xenograft tumors after JKE-1674 treatment. **E-I,** 2x10^{6} SPOP^{W131G} (Sp) LNCaP were subcutaneously injected into both flanks of male SCID mice. After tumors reached ~50mm^{3}, tumor-bearing mice were randomized and treated with JKE-1674 (15mg/kg; oral gavage; every other day) with or without Liproxastatin-1 (10mg/kg; i.p. injection; every day). Tumor volumes were measured twice a week using calipers. (E) Created in BioRender. Chen, F. (2026) https://BioRender.com/hlh47iw. **F-G,** Growth and weight of Sp xenograft tumors after JKE-1674/Liproxastatin-1 treatment. Numbers of tumors in each group: Vehicle n=4; JKE-1674 n=4; JKE-1674+Liproxastatin-1 n=5. **H,** Body weight of tumor-bearing mice during treatment. **I,** IHC staining of 4HNE in xenograft tumors after treatment. **J,** IHC staining of CHD1 and Flag-tagged SPOP^{F133V} in prostate tumors from P, PSp, and PSpC GEMM mice with JKE-1674 treatment. Scale bar, 100 µm. Data are presented as mean ± standard deviation, n = 3 biologically independent repeats. *P* values determined by unpaired two-tailed t test (A,F), one-way (G), or two-way (D) ANOVA analysis. Source data are provided as a Source Data file.

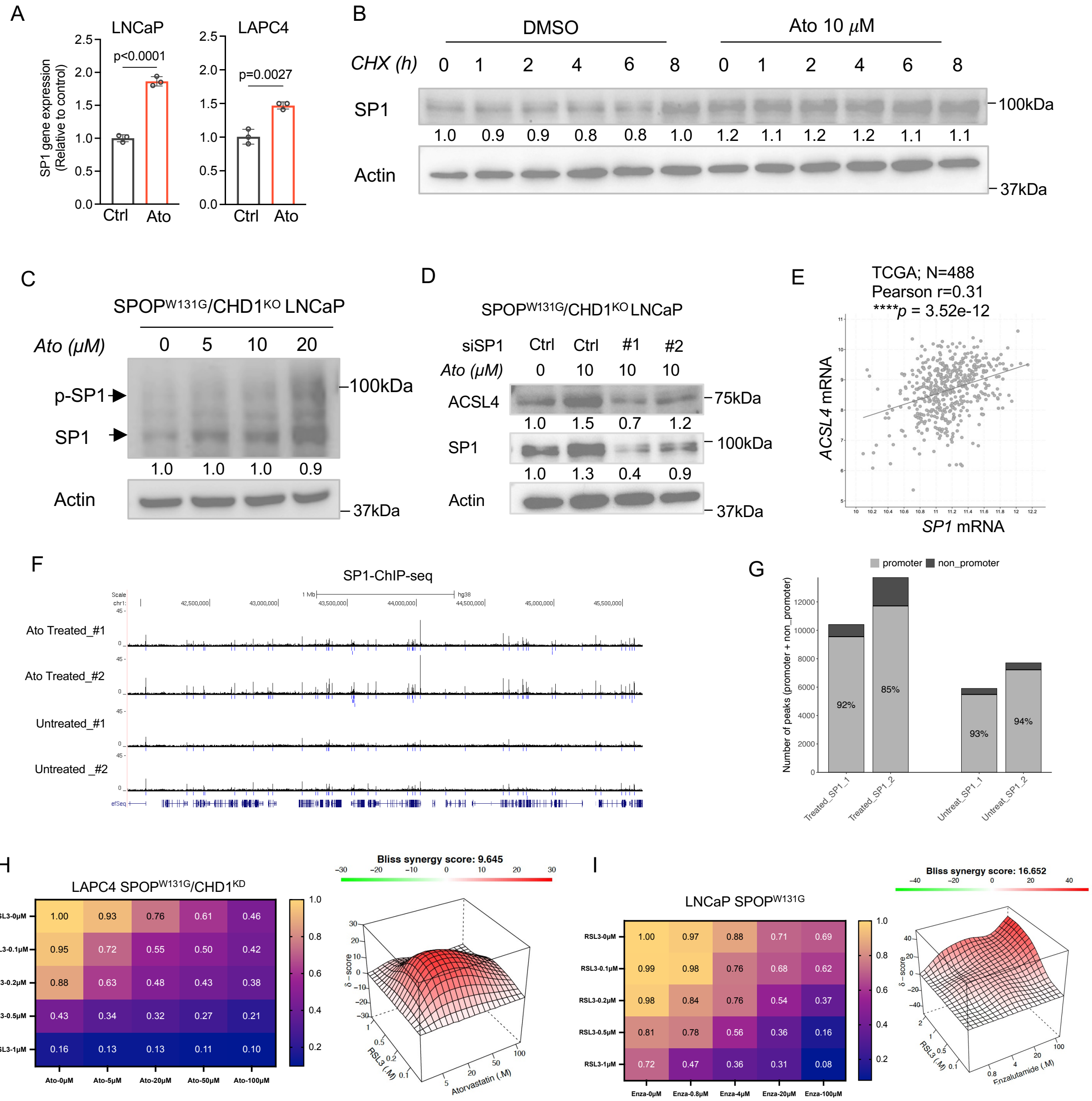

Figure S7. Cholesterol-lowering drugs restores ACSL4 in SPOP/CHD1 co-deficient PCa via activating SP1.

A, SP1 mRNA levels in SPOP^{W131G}/CHD1^{KO} LNCaP and SPOP^{W131G}/CHD1^{KD} LAPC4 cells after atorvastatin treatment (10 μM, 48 hr). **B,** SPOP^{W131G}/CHD1^{KO} LNCaP cells treated with 48h atorvastatin (10 μM), followed by the treatment of cycloheximide (CHX, 100 μg/ml) for the indicated times (0, 1, 2, 4, 6 and 8 hr). Protein stability of SP1 was determined by Western blot analysis. Relative density ratios of indicated proteins/actin are shown. **C,** Western blot analysis of phosphorylated SP1 (p-SP1) and unphosphorylated SP1 in SPOP^{W131G}/CHD1^{KO} LNCaP cells treated with different doses of Atorvastatin. Relative density ratios of p-SP1 versus total SP1 are shown. **D,** SP1 was knocked down in SPOP^{W131G}/CHD1^{KO} LNCaP cells, followed by treatment with atorvastatin (10 μM) for 48 hr. SP1 and ACSL4 protein levels were determined. Relative density ratios of indicated proteins/actin are shown. The samples derive from the same experiment but different gels for ACSL4, another for SP1, and another for Actin were processed in parallel. **E,** Scatter plots illustrating the correlations between ACSL4 and SP1 mRNA expression in human PCa samples (TCGA; n=488). The Pearson correlation coefficient (r) and *P* value are reported. **F-G,** SPOP^{W131G}/CHD1^{KO} LNCaP cells were treated with or without atorvastatin (10 μM) for 48h, followed by ChIP-seq using IgG control or SP1 antibodies. Representative landscape of SP1 in four samples was created using USCS genome browser (**F**). The number of peaks on promoter or none-promoters of SP1 in all samples are presented (**G**). **H,** Dose-response matrix (left) and drug interaction landscapes (right) revealing combination effects of RSL3 and atorvastatin on SPOP^{W131G}/CHD1^{KD} LAPC4 cells. **I,** Dose-response matrix (left) and drug interaction landscapes (right) revealing synergistic effects of RSL3 and enzalutamide on SPOP^{W131G} LNCaP cells. Drug interaction landscapes and synergy scores were generated using SynergyFinder. Data are presented as mean ± standard deviation, n = 3 biologically independent repeats. *P* values determined by unpaired two-tailed t test (A). Source data are provided as a Source Data file.

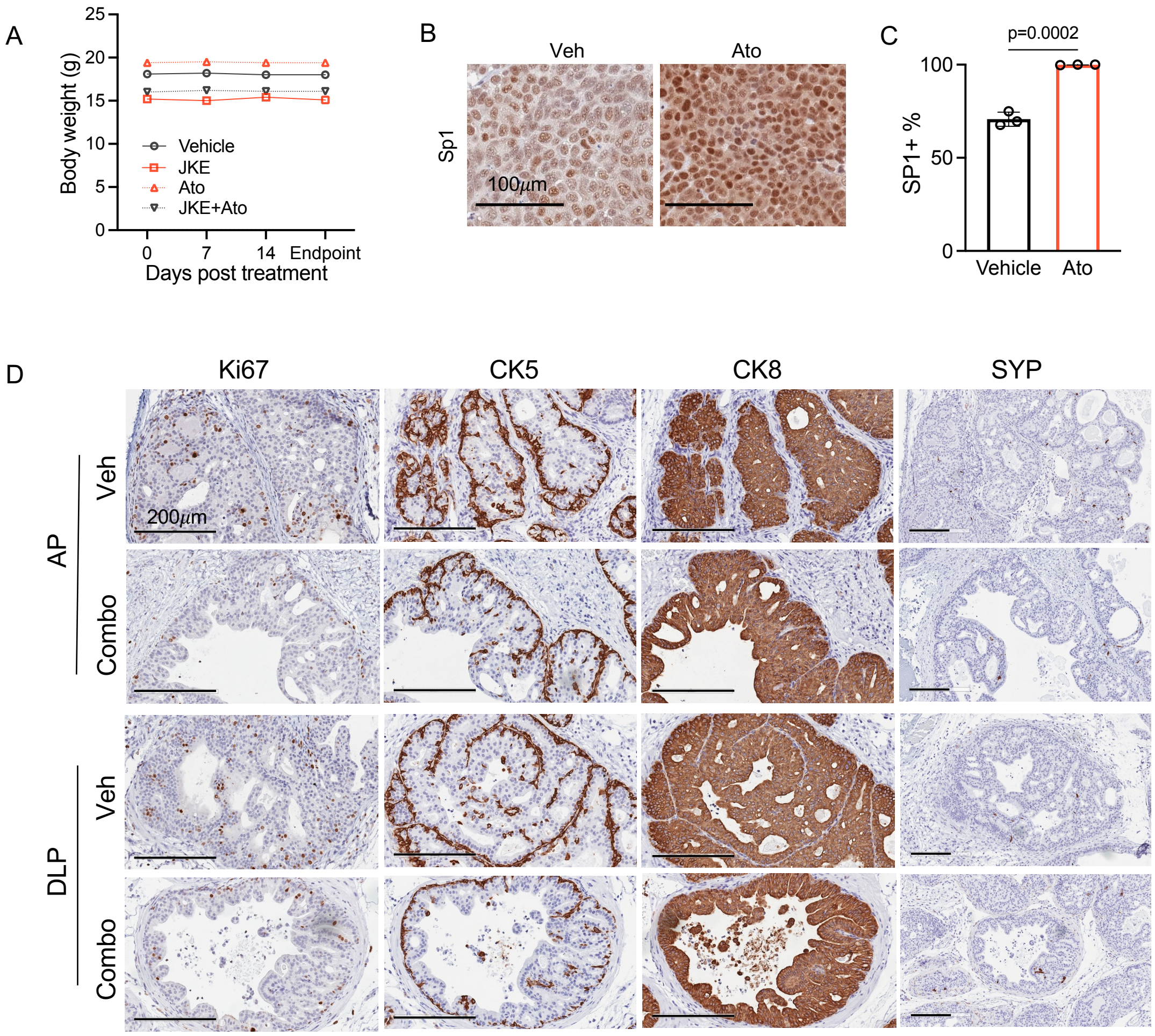

Figure S8. Cholesterol-lowering drugs re-sensitize SPOP/CHD1 co-deficient PCa to ferroptosis.

A, Body weight of tumor-bearing mice during single or combination treatments. **B-C,** IHC staining (**B**) and quantification (**C**) of SP1 in SpC xenograft tumors with or without atorvastatin treatment. Scale bar, 100 μm. **D,** Representative IHC staining of indicated markers in PSpC prostate tumors after vehicle or combination treatment. AP: anterior prostate; DLP: dorsolateral prostate. Scale bars, 200 μm. Data are presented as mean ± standard deviation, n = 3 biologically independent repeats. *P* values determined by unpaired two-tailed t test (C). Source data are provided as a Source Data file.
