# Supplementary material for: Divergent roles of SPOP and CHD1 in ACSL4 regulation reveal context-dependent vulnerabilities for targeting ferroptosis
Source: Nat Commun. 2026 Jun 30;17:8385. doi: 10.1038/s41467-026-75010-y (PMC13473601; doi:10.1038/s41467-026-75010-y)
Supplement: Supplementary file 2 [file 41467_2026_75010_MOESM2_ESM.pdf]

Description of Additional Supplementary Files

Supplementary Data 1. Lipidomic Profiling in SPOP-mutated LNCaP.xlsx

Lipidomic Profiling in LNCaP cells expressing WT or mutated SPOP (n=4 per group). The full names of lipid species are listed.

Supplementary Data 2. Summary of ACSL4 TF and SPOP substrates.xlsx

Lists of predicted transcriptional factors of ACSL4 (left) and known SPOP substrates (right).

Supplementary Data 3. Key reagents.xlsx

Information on key reagents, cell lines, animal strains, oligonucleotides, recombinant DNAs, software, and algorithms used in this study.
