# Supplementary material for: Divergent roles of SPOP and CHD1 in ACSL4 regulation reveal context-dependent vulnerabilities for targeting ferroptosis
Source: Nat Commun. 2026 Jun 30;17:8385. doi: 10.1038/s41467-026-75010-y (PMC13473601; doi:10.1038/s41467-026-75010-y)
Supplement: Supplementary file 3 [file 41467_2026_75010_MOESM6_ESM.pdf]

Corresponding author(s): Di Zhao
Last updated by author(s): Apr 15, 2026

Reporting Summary

Nature Portfolio wishes to improve the reproducibility of the work that we publish. This form provides structure for consistency and transparency in reporting. For further information on Nature Portfolio policies, see our Editorial Policies and the Editorial Policy Checklist.

Statistics

For all statistical analyses, confirm that the following items are present in the figure legend, table legend, main text, or Methods section.

n/a	Confirmed	
☐	☒	The exact sample size (*n*) for each experimental group/condition, given as a discrete number and unit of measurement
☐	☒	A statement on whether measurements were taken from distinct samples or whether the same sample was measured repeatedly
☐	☒	The statistical test(s) used AND whether they are one- or two-sided<br>*Only common tests should be described solely by name; describe more complex techniques in the Methods section.*
☒	☐	A description of all covariates tested
☐	☒	A description of any assumptions or corrections, such as tests of normality and adjustment for multiple comparisons
☐	☒	A full description of the statistical parameters including central tendency (e.g. means) or other basic estimates (e.g. regression coefficient) AND variation (e.g. standard deviation) or associated estimates of uncertainty (e.g. confidence intervals)
☐	☒	For null hypothesis testing, the test statistic (e.g. *F*, *t*, *r*) with confidence intervals, effect sizes, degrees of freedom and *P* value noted<br>*Give P values as exact values whenever suitable.*
☒	☐	For Bayesian analysis, information on the choice of priors and Markov chain Monte Carlo settings
☒	☐	For hierarchical and complex designs, identification of the appropriate level for tests and full reporting of outcomes
☐	☒	Estimates of effect sizes (e.g. Cohen's *d*, Pearson's *r*), indicating how they were calculated

Our web collection on statistics for biologists contains articles on many of the points above.

Software and code

Policy information about availability of computer code

Data collection	Aperio CS2 (Leica Biosystems) , Synergy 2 Multi-Mode Microplate reader (BioTek) were used to acquire images.<br>Synergy 2 Multi-Mode Microplate reader (BioTek) was utilized to measure luminescence and absorbance.<br>ChemiDoc Imaging System (Bio-Rad) was used for Western blot analysis.<br>QuantStudio 3 Real-Time PCR Systems (Thermo Fisher Scientific) was used for qPCR.<br>Bruker 7T MRI (Bruker Biospin MRI) was used for monitoring prostate tumor.<br>QuPath (v0.6.0-rc5) was used to for IHC stain quantification.
Data analysis	FlowJo (v10) was used to analyze the flow cytometry data.<br>ImageJ (v2.1.0) was used to observe and analyze MRI image, western blots data.<br>ImageScope software version 12.0.0 (Leica) was used to capture the slides images.<br>For clinical data, TCGA data from Bioportal was use.<br>IGV (v2.16.1) was used to visualize the peaks and alignments for ChIP-seq.<br>GraphPad Prism software (v10) was used to normalize and analyze data.<br>Synergyfinder (v3.0) was used for analyzing synergistic effects<br>Detailed information can be found in Method section.<br>The custom codes used for lipidomic profiling data analysis have been deposited in the public repository: https://github.com/MD-Anderson-Bioinformatics/xPEDITE.

For manuscripts utilizing custom algorithms or software that are central to the research but not yet described in published literature, software must be made available to editors and reviewers. We strongly encourage code deposition in a community repository (e.g. GitHub). See the Nature Portfolio guidelines for submitting code & software for further information.

Data

Policy information about availability of data

All manuscripts must include a data availability statement. This statement should provide the following information, where applicable:

- Accession codes, unique identifiers, or web links for publicly available datasets
- A description of any restrictions on data availability
- For clinical datasets or third party data, please ensure that the statement adheres to our policy

ChIP-sequencing data supporting this study's findings have been deposited in the Gene Expression Omnibus (GEO) under accession GSE317501. The lipidomic profiling data are available in Supplementary Data 1 and have been deposited in the Zenodo database under accession 19714093 [https://zenodo.org/records/19714093]. Human pan-cancer and prostate cancer genomic and bulk RNA-seq data were derived from the TCGA Research Network: http://cancergenome.nih.gov/. Source data for Western Blot images and Numerical results in all Figures and Supplementary Figures have been provided as Source Data files. All other data supporting the findings of this study are available from the corresponding author upon reasonable request.

Research involving human participants, their data, or biological material

Policy information about studies with human participants or human data. See also policy information about sex, gender (identity/presentation), and sexual orientation and race, ethnicity and racism.

Reporting on sex and gender	None
Reporting on race, ethnicity, or other socially relevant groupings	None
Population characteristics	None
Recruitment	None
Ethics oversight	None

Note that full information on the approval of the study protocol must also be provided in the manuscript.

Field-specific reporting

Please select the one below that is the best fit for your research. If you are not sure, read the appropriate sections before making your selection.

☒ Life sciences ☐ Behavioural & social sciences ☐ Ecological, evolutionary & environmental sciences

For a reference copy of the document with all sections, see nature.com/documents/nr-reporting-summary-flat.pdf

Life sciences study design

All studies must disclose on these points even when the disclosure is negative.

Sample size	Power analysis was not performed to predetermine the sample size. Sample size was determined based on previous published reports (PMID: 35771632, 40360905) and experimental experience.
Data exclusions	No data excluded.
Replication	All the experiments were performed at least three times with similar results.
Randomization	Mice of matched age, tumor samples, and cell line cultures were randomly assigned to experimental groups.
Blinding	The histopathological experiments were performed and reviewed in a blinded manner. All experiments were performed and analyzed blinded to the genotype, group, and treatment whenever possible.

Reporting for specific materials, systems and methods

We require information from authors about some types of materials, experimental systems and methods used in many studies. Here, indicate whether each material, system or method listed is relevant to your study. If you are not sure if a list item applies to your research, read the appropriate section before selecting a response.

Materials & experimental systems

n/a	Involved in the study
[ ]	[x] Antibodies
[ ]	[x] Eukaryotic cell lines
[x]	[ ] Palaeontology and archaeology
[ ]	[x] Animals and other organisms
[x]	[ ] Clinical data
[x]	[ ] Dual use research of concern
[x]	[ ] Plants

Methods

n/a	Involved in the study
[ ]	[x] ChIP-seq
[ ]	[x] Flow cytometry
[x]	[ ] MRI-based neuroimaging

Antibodies

Antibodies used

All antibodies used are commercially available and validated by the manufacturers.
Antibodies used for IHC are as below:
anti-CHD1 (Sigma-Aldrich, HPA022236, Clone NA), Dilution 1:500
anti-Flag (Cell Signaling Technology, 14793, Clone NA), Dilution 1:500
anti-AR (Sigma-Aldrich, 06-680, Clone NA), Dilution 1:500
anti-cleaved caspase-3 (Cell Signaling Technology, 9661, Clone NA), Dilution 1:500
anti-Ki67 (Thermo Fisher Scientific, RM-9106-S1, Clone: SP6), Dilution 1:500
anti-ACSL4 (Santa Cruz, sc-271800, Clone A-5), Dilution 1:200
anti-cMyc (Cell Signaling Technology, 18583), Dilution 1:100
anti-SP1 (Cell Signaling Technology, 9389, Clone NA), Dilution 1:100
anti-4HNE (Abcam, ab46545, Clone NA), Dilution 1:400

Antibodies used for Western blot are as below, dilution is 1:1000 unless otherwise stated:
anti-β-actin (Sigma, A5441, Clone NA), Dilution 1:5000;
anti-V5 Tag (Invitrogen, 46-1157, Clone NA);
anti-CHD1 (D8C2) (Cell Signaling Technology, 4351, Clone NA);
anti-ACSL4 (Santa Cruz, sc-271800, Clone A-5);
anti-SP1 (Cell Signaling Technology, 9389, Clone NA);
anti-ATF2 (Cell Signaling Technology, 35031, Clone NA);
anti-cMyc (Cell Signaling Technology, 18583);
GPX4 Monoclonal antibody (Proteintech Cat# 67763-1-Ig);
DHODH Polyclonal antibody (Proteintech Cat# 14877-1-AP);
xCT/SLC7A11 (D2M7A) Rabbit mAb (Cell Signaling Technology Cat# 12691);
AIFM2/FSP1 Polyclonal antibody (Proteintech Cat# 20886-1-AP);
NRF2 (D1Z9C) XP® Rabbit mAb (Cell Signaling Technology Cat# 12721S);
GCH1 Monoclonal Antibody (OTI1E3) (Invitrogen Cat# MA5-27277);
anti-rabbit IgG (HRP) (Cell Signaling Technology, 7074V, Clone NA), Dilution 1:5000;
anti-mouse IgG (HRP) (Cell Signaling Technology, 7076V, Clone NA), Dilution 1:5000.

Antibodies used for IP are as below, dilution is 1:100 unless otherwise stated:
anti-CHD1 (Bethyl, A301-218A, Clone NA);
anti-cMyc (Cell Signaling Technology, 18583);
normal rabbit IgG (Sigma, 12-370, Clone NA), Dilution 1:500.

Antibodies used for ChIP/ChIP-seq are as below:
c-Myc (18583S, Cell Signaling Technology), Dilution 1:100;
CHD1 (4351S, Cell Signaling Technology), Dilution 1:100;
SP1 (9389S, Cell Signaling Technology), Dilution 1:50.

Validation

anti-CHD1 (Sigma-Aldrich, RM-9107), anti-AR (Sigma-Aldrich, 06-680), anti-cleaved caspase-3 (Cell Signaling Technology, 9661), anti-Ki67 (Thermo Fisher Scientific, RM-9106-S1), anti-CHD1 (D8C2) (Cell Signaling Technology, 4351), anti-V5 Tag (Invitrogen, 46-1157) have been validated in our previous publications: PMID: 32385075, 40360905. For anti-rabbit IgG (HRP) (Cell Signaling Technology, 7074V); anti-mouse IgG (HRP) (Cell Signaling Technology, 7076V), anti-β-actin (Sigma-Aldrich, A5441-.2ML), normal rabbit IgG (Sigma, 12-370) have been validated in our previous publications: PMID: 35771632 and PMID: 40360905.

The validation of other antibodies was provided by the manufacturer's website:
anti-ACSL4 (Santa Cruz, sc-271800, Clone A-5); https://www.scbt.com/p/acsl4-antibody-a-5?srsltid=AfmBOooer1bYHoF0rwyK-z-U7PNPVueAgUEsJIPrYkTbnAw8f8tsS-vC.
anti-SP1 (Cell Signaling Technology, 9389, Clone NA); https://www.cellsignal.com/products/primary-antibodies/sp1-d4c3-rabbit-mab/9389?srsltid=AfmBOoqKxzWdup2WXphAdvZi4ALal7ngG66suTKpPCngVOr0mUBlIBL8.
anti-ATF2 (Cell Signaling Technology, 35031, Clone NA); https://www.cellsignal.com/products/primary-antibodies/atf-2-d4l2x-xp-rabbit-mab/35031?srsltid=AfmBOopJraEacx48XtaOSsK1n_9vdvyxeDXRIqcTRB9WxAoLzBLXNco6.
anti-cMyc (Cell Signaling Technology, 18583); https://www.cellsignal.com/products/primary-antibodies/c-myc-e5q6w-rabbit-

mab/18583?srsltid=AfmBOoqjTc7O4x2lWFKan4xXhwXuEv_DkNN8X7Z43bLZX80MkpnXnIbS.
anti-4HNE (Abcam, ab46545, Clone NA); https://www.abcam.com/en-us/products/primary-antibodies/4-hydroxynonenal-antibody-ab46545.
GPX4 Monoclonal antibody (Proteintech Cat# 67763-1-Ig); https://www.ptglab.com/products/GPX4-Antibody-67763-1-Ig.htm?srsltid=AfmBOop00Xj92V-n9ziMQQEdvKvyPiqVt1wyem1f9rAI4qoWUtMbqmo3.
DHODH Polyclonal antibody (Proteintech Cat# 14877-1-AP); https://www.ptglab.com/products/DHODH-Antibody-14877-1-AP.htm?srsltid=AfmBOopY4lOyploB4Tj6_UMxKjBTj4rRjhezv1CyWw3zR_Ld02pqOySD.
xCT/SLC7A11 (D2M7A) Rabbit mAb (Cell Signaling Technology Cat# 12691); https://www.cellsignal.com/products/primary-antibodies/xct-slc7a11-d2m7a-rabbit-mab/12691?srsltid=AfmBOooFV5nELsgmPIv5ND9f3rmwBjoyvxPCo3vU0m79vTMzd5ibjM_q.
AIFM2/FSP1 Polyclonal antibody (Proteintech Cat# 20886-1-AP); https://www.ptglab.com/products/AIFM2-Antibody-20886-1-AP.htm?srsltid=AfmBOor1Z6Lad03PdobepQpVGLGfFC6R60CfqzTrDHR8qPsI6gCO1LVh.
NRF2 (D1Z9C) XP® Rabbit mAb (Cell Signaling Technology Cat# 12721S); https://www.cellsignal.com/products/primary-antibodies/nrf2-d1z9c-xp-rabbit-mab/12721?srsltid=AfmBOor8PaDHUoKA7x4X8tSi7INFt1CxPvNGZw8r461vd5V-1QfH0qsa.
GCH1 Monoclonal Antibody (OTI1E3) (Invitrogen Cat# MA5-27277); https://www.thermofisher.com/antibody/product/GCH1-Antibody-clone-OTI1E3-Monoclonal/MA5-27277.

Eukaryotic cell lines

Policy information about cell lines and Sex and Gender in Research

Cell line source(s)	293T cells and prostate cancer cell lines (LNCap, 22Rv-1) were obtained from American Type Culture Collection (ATCC). LAPC4 cell line was a gift of Dr. Frigo's lab. Organoids cell lines were generated from our GEMM model and validated by western blot.
Authentication	All of the cell lines were confirmed by using STR profiling at The University of Texas MD Anderson Cancer Center's Cytogenetic and Cell Authentication Core
Mycoplasma contamination	All of the cell lines were tested to be negative for mycoplasma using MycoAlert PLUS detection kit (Lonza, LT07-710)
Commonly misidentified lines (See ICLAC register)	No commonly misidentified lines were used

Animals and other research organisms

Policy information about studies involving animals; ARRIVE guidelines recommended for reporting animal research, and Sex and Gender in Research

Laboratory animals	The Pb-Cre; PtenL/L (PbP) mouse strain was a gift from Dr. Ronald DePinho's research group at MD Anderson (Houston, TX).<br>The Pb-Cre; Pten L/L CHD1L/L (PbPC) mouse was established in our previous study PMID: 32385075.<br>SPOP-F133V transgene allele was purchased from the Jackson Laboratory (cat #030102) and was crossed onto Pb-Cre; PtenL/L (PbP) mice or Pb-Cre; Pten L/L CHD1L/L (PbPC) mice to generate a prostate-specific SPOP-F133V transgenic model (PbPSp) or Pb-Cre; Pten L/L CHD1L/L SPOPF133V (PbPCSp). These models were established in our previous study PMID: 40360905.<br>All transgenic mice used in this study were male with >90% C57BL/6J background. Mice were interbred, regularly genotyped, and maintained at the MD Anderson animal facility. All methods were reviewed and approved by MD Anderson's Institutional Animal Care and Use Committee (protocol #00001955).<br><br>For the xenograft PCa model, 4-week-old male SCID mice (CB17/Icr-Prkdcscid/IcrIcoCrl) were purchased from Charles River and transplanted with cancer cells.<br><br>The mice were housed under a 12-hour light/dark cycle, at 68-78°F with 30–70% humidity, following institutional guidelines.
Wild animals	The study did not involve wild animals.
Reporting on sex	For prostate cancer study, only male mice were included in this research.
Field-collected samples	No field collected samples were used in the study.
Ethics oversight	All manipulations were performed under the review and approval of MD Anderson Cancer Center's Institutional Animal Care and Use Committee (Protocol #00001955).

Note that full information on the approval of the study protocol must also be provided in the manuscript.

Plants

Seed stocks: Not applicable

Novel plant genotypes: Not applicable

Authentication: Not applicable

ChIP-seq

Data deposition

[x] Confirm that both raw and final processed data have been deposited in a public database such as GEO.

[x] Confirm that you have deposited or provided access to graph files (e.g. BED files) for the called peaks.

Data access links
May remain private before publication.

GSE317501 (https://www.ncbi.nlm.nih.gov/geo/query/acc.cgi?acc=GSE317501); Reviewer token: wvubimcmzhqzner

Files in database submission: FASTQ files, bigWig files.

Genome browser session
(e.g. UCSC)

Not applicable

Methodology

Replicates: Two biological replicates

Sequencing depth

Numbers of fragments (read pairs):
Sample uniquely_mapped uniquely_mapped_after_deduplication
Treated_Input 48927735 37479936 32461567
Treated_SP1_CHIP_1 48822849 37475048 31759692
Treated_SP1_CHIP_2 50179999 38137972 32729687
Untreat_Input 56831745 43260158 36577233
Untreat_SP1_CHIP_1 59843667 45527477 38393643
Untreat_SP1_CHIP_2 57102376 43661770 37242263
W131G_CHD1_CHIP_1 48774824 36941078 31757244
W131G_CHD1_CHIP_2 56342644 42719499 36132002
W131G_Input 51403836 38802368 33770701
WT_CHD1_CHIP_1 50547378 38538208 32343532
WT_CHD1_CHIP_2 61261433 46060044 38662951
WT_Input 57661539 43172442 36079486

Read length: 101 bp; Paired-end.

Antibodies: CHD1 (Dilution 1:100, 4351S, Cell Signaling Technology), SP1 (Dilution 1:50, 9389S, Cell Signaling Technology).

Peak calling parameters

Adapter sequences were removed from the 3' ends of reads using Trim Galore (version 0.6.7) and cutadapt (version 4.1) . The reads were then mapped to the human genome (hg38) using Bowtie (version 1.1.2) with the following parameters: ""--allow-contain --maxins 2000 -v 2 -m 1 --best --strata". To avoid PCR bias, only one copy of multiple fragments mapped to the same genomic position was retained for further analysis. Fragments longer than 500 bp (less than 1% of the total) were excluded from subsequent analysis. peaks were identified using MACS (version 1.4.2) by comparing against the corresponding input sample. A window size of 300 bp was used, and a p-value cutoff of 1e-5 was applied. The peaks that overlapped with ENCODE blacklisted regions were removed.

Data quality

Overall, 76–78% of fragments (read pairs) mapped uniquely to the human genome. Library complexity was high, with non-redundant fraction (NRF) values of 0.84–0.87. After filtering, 32–39 million fragments per sample were retained for peak calling.

The number of peaks identified and the associated empirical false discovery rate (FDR) are shown below.

Sample #Peaks Empirical_FDR
Treated_SP1_CHIP_1 10,404 .60%
Treated_SP1_CHIP_2 13,721 0.50%
Untreat_SP1_CHIP_1 5,909 0.79%

	Untreat_SP1_CHIP_2 7,709 0.60%<br>W131G_CHD1_CHIP_1 11,019 0.96%<br>W131G_CHD1_CHIP_2 12,721 0.74%<br>WT_CHD1_CHIP_1 1,489 1.71%<br>WT_CHD1_CHIP_2 2,243 1.45%
Software	Trim Galore (version 0.6.7)<br>cutadapt (version 4.1)<br>Bowtie (version 1.1.2)<br>MACS (version 1.4.2)

Flow Cytometry

Plots

Confirm that:

☒ The axis labels state the marker and fluorochrome used (e.g. CD4-FITC).

☒ The axis scales are clearly visible. Include numbers along axes only for bottom left plot of group (a 'group' is an analysis of identical markers).

☒ All plots are contour plots with outliers or pseudocolor plots.

☒ A numerical value for number of cells or percentage (with statistics) is provided.

Methodology

Sample preparation	Cells were washed in PBS once and stained in PI or BODIPY 581/591 for 15 mins
Instrument	Attune NxT Flow Cytometer
Software	FlowJo 10
Cell population abundance	At least 10,000 single cells were collected per sample
Gating strategy	FSC/SSC for LNCap cells is 85/300

☒ Tick this box to confirm that a figure exemplifying the gating strategy is provided in the Supplementary Information.
