# Supplementary material for: Divergent roles of SPOP and CHD1 in ACSL4 regulation reveal context-dependent vulnerabilities for targeting ferroptosis
Source: Nat Commun. 2026 Jun 30;17:8385. doi: 10.1038/s41467-026-75010-y (PMC13473601; doi:10.1038/s41467-026-75010-y)
Supplement: Supplementary file 4 [file 41467_2026_75010_MOESM7_ESM.pdf]

nature portfolio

Peer Review File

Divergent Roles of SPOP and CHD1 in ACSL4 Regulation Reveal Context-dependent Vulnerabilities for Targeting Ferroptosis

Corresponding Author: Dr Di Zhao

This file contains all reviewer reports in order by version, followed by all author rebuttals in order by version.

Version 0:

Reviewer comments:

Reviewer #1

(Remarks to the Author)
In their manuscript entitled “Divergent Roles of SPOP and CHD1 in ACSL4 Regulation Reveal Context-dependent Vulnerabilities for Targeting Ferroptosis in Prostate Cancer,” Chen et al. present a compelling and well-executed study that integrates cellular models, organoids, xenografts, and genetically engineered mouse models (GEMMs) harboring SPOP mutations and CHD1 deletions. Their findings provide a strong rationale for targeting SPOP-mutant tumors with ferroptosis-inducing therapies. Moreover, they propose an exciting pharmacological strategy to re-sensitize SPOP-mutant/CHD1-deleted tumors to ferroptosis by combining these therapies with cholesterol-lowering agents.
Given that approximately 15% of prostate cancer patients harbor SPOP mutations, this study has the potential to impact a significant number of individuals, considering the high prevalence of the disease. Importantly, the authors also elucidate the mechanistic basis of this vulnerability, demonstrating that MYC-mediated regulation of ACSL4 expression underlies the phenotype in SPOP-mutant tumors, with additional modulation through CHD1/SP1 interplay.
Altogether, this is a timely and impactful study that merits consideration for publication, pending the authors address the following comments to further strengthen the manuscript:

Abstract: Mention that “SPOP mutations enhance, whereas CHD1 deletion impairs, the efficacy of ferroptosis inducers targeting GPX4”. Therefore, how can “cholesterol-lowering agents restores ACSL4 expression and re-sensitize SPOP-mutated/CHD1-deleted tumors” since these were sensitive in the first place? Upon reading the manuscript, it becomes evident that the authors refer to the combined SPOP-mutant/CHD1-deleted context. This distinction should be made explicit in the abstract to avoid confusion.

Fig. 3 and Fig 7: The efficacy of siMYC knockdown should be validated by western blot to confirm MYC suppression. Similarly, the effectiveness of siSP1 should be demonstrated via western blot.

MYC/CHD1 regulation of ACSL4: The manuscript states that “CHD1 and c-Myc proteins bind the ACSL4 promoter region (Fig. 5H),” yet the CHD1 ChIP-seq data focuses on the ACSL4 gene body rather than the promoter. While the luciferase assay (Fig. 3I) suggests MYC regulation of ACSL4, this should be complemented with a MYC ChIP-seq experiment to demonstrate direct binding near the ACSL4 promoter. Sun et al. (PMID: 39604370) previously showed MYC binding at the ACSL4 promoter, but it remains unclear whether this is the regulatory element relevant in the context of SPOP mutations. To enhance the novelty and mechanistic depth, the authors should provide a comprehensive ChIP-seq analysis of MYC and CHD1 binding near ACSL4 in both SPOP wild-type and mutant backgrounds. Additionally, SP1 ChIP-seq data should be included in Fig. 7 to support the proposed regulatory model.

Western blot: Densitometric analysis should be performed for all western blots and reported as numerical values (normalized to control) beneath each image. This would provide a semi-quantitative assessment of the magnitude of changes, such as the increase in ACSL4 following Ato treatment in Fig. 7B.

Reviewer #2

(Remarks to the Author)

The manuscript by Feiyu Chen et al. presents an interesting and well-structured study addressing the genetic determinants of ferroptosis susceptibility in prostate cancer. The authors demonstrate that SPOP mutations and CHD1 deletions define distinct molecular subtypes with increased sensitivity to ferroptosis inducers, highlighting the MYC–ACSL4 axis as a key regulatory pathway. Importantly, the translational potential of the work is underscored by the finding that cholesterol-lowering agents can restore ferroptosis sensitivity in resistant tumors, suggesting novel biomarker-driven therapeutic strategies for advanced prostate cancer. I believe this manuscript has strong potential for publication in Nature Communications, pending clarification of the following points.

Major point:
1. The authors discuss that SPOP mutations increase ACSL4 levels, thereby sensitizing cells to ferroptosis. While this is an interesting finding, a global lipidomics analysis would be essential to provide a broader understanding of which lipid species are altered upon SPOP mutation. Inclusion of such data, or at least a discussion of this point, would strengthen the manuscript.
2. The authors report that c-Myc levels increase upon SPOP mutation. It should be clarified whether overexpression of MYC in SPOP wild-type cells can also increase ferroptosis sensitivity, similar to previous findings showing that MYCN-amplified neuroblastoma cells are sensitized to ferroptosis and rely on selenocysteine uptake and metabolism.
3. It would be important to assess whether SPOP mutations and the observed c-Myc changes have an impact on other ferroptosis regulators, such as FSP1 (GSH-independent pathway) and SLC7A11, which play key roles in selenium metabolism and glutathione synthesis, as well as on GCLM, ALDH, and other MYC targets. More global molecular experiments, such as proteomics or metabolomics, could help address this, followed by targeted validation focusing on key ferroptosis modulators, including FSP1, SLC7A11, GPX4, LRP8, and PRDX6.
4. The authors should clarify whether the effect of SPOP mutations on ferroptosis is restricted to prostate cancer, or if it extends to other cancer types. Testing additional cancer cell lines would add valuable context.

Minor points:
1. In Figure 1, the authors show that SPOP mutations sensitize cells to RSL3. It is important to explain the rationale for using relatively high concentrations of RSL3 in the µM range, since most cancer cell lines are highly sensitive to RSL3 at much lower (nM) doses. The authors should clarify why such high doses were chosen for their experimental setup. Testing other classes of ferroptosis-inducing compounds, such as erastin, could help strengthen this observation.
2. In Figure 6a, it is not clear how many mice were used in each experimental group. The authors should provide the number of animals per group to ensure clarity and reproducibility.

Reviewer #3

(Remarks to the Author)
This manuscript by Chen et al. investigates the important roles of SPOP mutations and CHD1 deletions in regulating ferroptosis susceptibility in prostate cancer. The authors propose a mechanistic model whereby SPOP mutations stabilize c-MYC, which in cooperation with CHD1 activates ACSL4 transcription, thereby sensitizing tumors to ferroptosis. Conversely, CHD1 loss abrogates this process, conferring ferroptosis resistance. Importantly, the authors demonstrate that cholesterol-lowering agents (statins) restore ACSL4 expression via a CHD1-independent cholesterol–SP1–ACSL4 axis, thereby re-sensitizing engineered cell lines, GEMMs, organoids, and xenografts. Overall, this is a timely and impactful study that addresses an unmet need of identifying genetic biomarkers that dictate ferroptosis susceptibility. The mechanistic depth, complementary use of models, and translational perspective make this work significant.

Major Concerns
1. While the ChIP experiments convincingly show that CHD1 facilitates c-MYC binding to the ACSL4 promoter, the broader chromatin remodeling function of CHD1 in this context is less directly addressed. Techniques such as ATAC-seq or CUT&RUN profiling would provide deeper resolution of how CHD1 loss alters global chromatin accessibility, thereby strengthening the mechanistic framework.

2. The identification of a CHD1-independent cholesterol–SP1–ACSL4 pathway is intriguing but remains less deeply explored compared to the SPOP–CHD1–MYC axis. The authors should provide direct evidence that SP1 binds to the ACSL4 promoter under cholesterol-depleted conditions. In addition, the mechanism linking cholesterol depletion to SP1 upregulation remains unclear. Does cholesterol loss affect SP1 protein stability, nuclear localization, or post-translational modification? Clarifying this regulatory step would significantly strengthen the proposed compensatory pathway.

3. While ACSL4 expression correlations with SPOP/CHD1 alterations are shown using TCGA, additional clinical analyses would enhance the translational impact. Specifically (1) The authors should explicitly state the number of TCGA cases analyzed in the figure legends to ensure transparency. (2) Independent validation cohorts (SU2C or MSK-IMPACT) would strengthen the robustness of the correlations. (3) It would be informative to test whether ACSL4 expression correlates with clinical outcomes (survival or therapy response) in SPOP-mutant versus CHD1-deficient tumors.

4. The authors conclude that SPOP mutations sensitize to ferroptosis in an androgen-independent manner. Given prior evidence that AR signaling regulates ferroptosis-related enzymes, a systematic analysis of AR activity under ferroptosis-inducing conditions would clarify whether AR activity modulates the observed phenotypes. Testing the combination of a GPX4 inhibitor with a potent AR antagonist in SPOP-mutated, CHD1-intact models would also provide a complete therapeutic roadmap.

5. Although the mechanistic link between CHD1 loss and ACSL4 silencing is well supported in models, protein-level validation in independent human PCa tumor samples would provide important clinical confirmation that CHD1 deletion consistently associates with reduced ACSL4 expression.

6. The proposed statin and GPX4 inhibitor strategy is compelling. A powerful complementary analysis would investigate whether PCa patients with SPOP-mut/CHD1-deficient tumors who were incidentally prescribed statins in the clinic had improved outcomes compared to those who were not. Furthermore, since SPOP/CHD1-deficient tumors are aggressive and prone to lineage plasticity, the authors should examine whether long-term treatment of xenografts or GEMMs induces markers of lineage switching (NEPC or basal differentiation), which would indicate potential escape routes from ferroptosis-inducing pressure.

Minor Points

1. Figures are generally clear, but some would benefit from more detailed legends (e.g., organoid dose–response curves, ChIP assays).
2. Statistical methods are appropriate, but additional detail on sample size justification and blinding would improve rigor.
3. In figures illustrating patient data correlations (e.g., TCGA scatter plots), the number of samples analyzed should be clearly indicated in the legends.

Version 1:

Reviewer comments:

Reviewer #1

(Remarks to the Author)
The revised manuscript thoughtfully addresses the reviewers' comments and further strengthens an already timely, impactful, and compelling study. The added experiments, particularly the untargeted lipidomics, the ChIP-seq analyses, and the JKE-1674/atorvastatin combination studies in PSpC GEMMs, represent meaningful enhancements. Provided the authors address the following minor points, I believe the manuscript is suitable for publication.

Minor point:
1. IC50: Please report IC50 values in Figure 1C, E, F, G, Figure 2I, J, Figure 3G, Figure 4A, C and Figure 5F as well as in the relevant supplementary figures.
2. Figure 4E: Based on the figure label, it appears the comparison is CHD1 KO versus WT cells; however, the text suggests the intended comparison is CHD1 KO in a SPOP-mutant background versus SPOP-mutant cells. Please clarify the experimental context and labeling.
3. Line 253 / Line 336: "CHD1-binding peaks" / "SP1-binding peaks" should be revised to "CHD1-binding sites" / "SP1-binding sites", respectively.
4. Line 273: The manuscript states that "loss of CHD1 abrogated c-Myc binding to target genes…," but the data presented demonstrate reduced MYC binding specifically at the ACSL4 promoter. Please revise the sentence to reflect this, unless additional evidence is provided to support a broader effect on multiple MYC target genes.
5. Figure S8E: This new panel should be introduced in the Results section (prior to its mention in the Discussion on line 430).
6. Endometrial carcinomas (EC; line 460): The basis for the statement that EC-associated SPOP mutations (M117V, D140G, E47K, and E50K) do not affect ACSL4 induction or ferroptosis sensitivity is unclear. If these data are included (e.g., in Rebuttal Figure 1), they should be incorporated as a supplementary figure and appropriately referenced in the manuscript; otherwise, the statement should be removed from the Discussion
7. ChIP-seq tracks: Please include the MACS-called peak regions (peak calls) beneath the corresponding genome browser tracks to facilitate interpretation.

Reviewer #2

(Remarks to the Author)
The authors have adequately addressed all raised questions, the results are noteworthy and significant to the field of ferroptosis and related areas, and I therefore support publication in Nature Communications.

Reviewer #3

(Remarks to the Author)
The authors have addressed most of my concerns and comments, and the revised manuscript has been significantly improved. I appreciate the effort the authors have put into revising the manuscript and responding to the reviewers' suggestions. Overall, the study is now much clearer and stronger.

Open Access This Peer Review File is licensed under a Creative Commons Attribution 4.0 International License, which permits use, sharing, adaptation, distribution and reproduction in any medium or format, as long as you give appropriate credit to the original author(s) and the source, provide a link to the Creative Commons license, and indicate if changes were made.
In cases where reviewers are anonymous, credit should be given to 'Anonymous Referee' and the source.
The images or other third party material in this Peer Review File are included in the article's Creative Commons license, unless indicated otherwise in a credit line to the material. If material is not included in the article's Creative Commons license and your intended use is not permitted by statutory regulation or exceeds the permitted use, you will need to obtain permission directly from the copyright holder.
To view a copy of this license, visit https://creativecommons.org/licenses/by/4.0/

REVIEWER COMMENTS

Reviewer #1 (Remarks to the Author):

In their manuscript entitled "Divergent Roles of SPOP and CHD1 in ACSL4 Regulation Reveal Context-dependent Vulnerabilities for Targeting Ferroptosis in Prostate Cancer," Chen et al. present a compelling and well-executed study that integrates cellular models, organoids, xenografts, and genetically engineered mouse models (GEMMs) harboring SPOP mutations and CHD1 deletions. Their findings provide a strong rationale for targeting SPOP-mutant tumors with ferroptosis-inducing therapies. Moreover, they propose an exciting pharmacological strategy to re-sensitize SPOP-mutant/CHD1-deleted tumors to ferroptosis by combining these therapies with cholesterol-lowering agents.
Given that approximately 15% of prostate cancer patients harbor SPOP mutations, this study has the potential to impact a significant number of individuals, considering the high prevalence of the disease. Importantly, the authors also elucidate the mechanistic basis of this vulnerability, demonstrating that MYC-mediated regulation of ACSL4 expression underlies the phenotype in SPOP-mutant tumors, with additional modulation through CHD1/SP1 interplay.
Altogether, this is a timely and impactful study that merits consideration for publication, pending the authors address the following comments to further strengthen the manuscript:

Response: We thank this reviewer's positive feedback on the rationale, data quality, and significance of our study! We also appreciate his/her constructive comments. To address these comments and strengthen the manuscript's major conclusions, we included new results and evidence, quantified all the Western blots, and revised the manuscript. The manuscript has been significantly improved after revision. Please find our point-by-point responses below.

1. Abstract: Mention that "SPOP mutations enhance, whereas CHD1 deletion impairs, the efficacy of ferroptosis inducers targeting GPX4". Therefore, how can "cholesterol-lowering agents restores ACSL4 expression and re-sensitize SPOP-mutated/CHD1-deleted tumors" since these were sensitive in the first place? Upon reading the manuscript, it becomes evident that the authors refer to the combined SPOP-mutant/CHD1-deleted context. This distinction should be made explicit in the abstract to avoid confusion.

Response: The abstract has been revised into "*Furthermore, we demonstrate that targeting cholesterol metabolism with cholesterol-lowering agents restores ACSL4 expression and re-sensitizes SPOP/CHD1 co-deficient tumors to ferroptosis-inducing therapy (Line 45)*". The term "SPOP-mutated/CHD1-deleted tumors" has been replaced with "SPOP/CHD1 co-deficient tumors" throughout the manuscript to avoid confusion and increase the readability.

2. Fig. 3 and Fig 7: The efficacy of siMYC knockdown should be validated by western blot to confirm MYC suppression. Similarly, the effectiveness of siSP1 should be demonstrated via Western blot.

Response: As suggested, we verified the knockdown efficiencies of MYC and SP1 using Western blot in Figures 3 and 7. Please find the new results below and in Figure S3D and S7D. Quantification of Western blots is also shown.

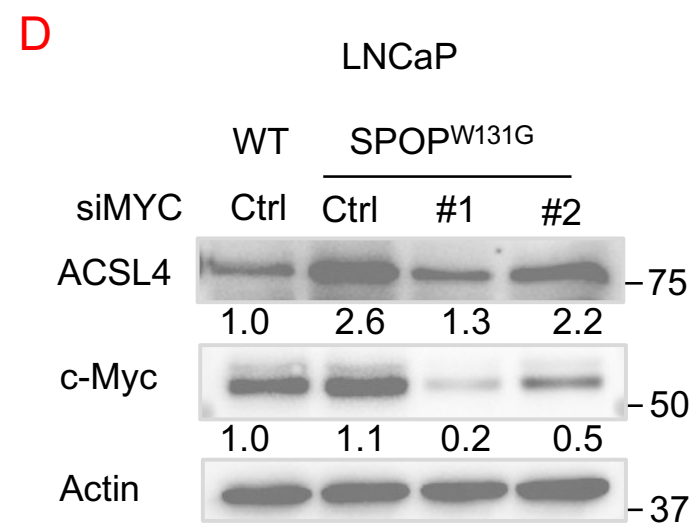

Figure S3D. MYC was knocked down in WT and SPOP^{W131G} LNCaP cells using two individual siRNAs, followed by determining c-Myc and ACSL4 protein expression using Western blot. Relative density ratios of indicated proteins/actin are shown.

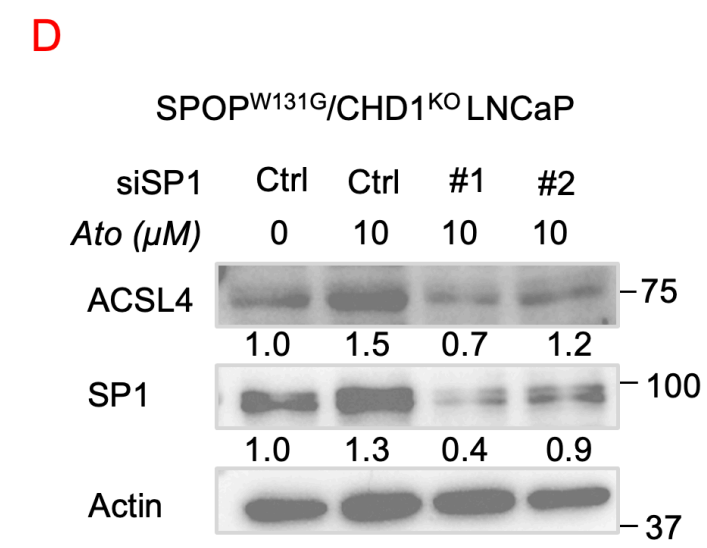

Figure S7D. SP1 was knocked down in SPOP^{W131G}/CHD1^{KO} LNCaP cells, followed by treatment with atorvastatin (10 μM) for 48 hr. SP1 and ACSL4 protein levels were determined. Relative density ratios of indicated proteins/actin are shown.

3. MYC/CHD1 regulation of ACSL4: The manuscript states that "CHD1 and c-Myc proteins bind the ACSL4 promoter region (Fig. 5H)," yet the CHD1 ChIP-seq data focuses on the ACSL4 gene body rather than the promoter. While the luciferase assay (Fig. 3I) suggests MYC regulation of ACSL4, this should be complemented with a MYC ChIP-seq experiment to demonstrate direct binding near the ACSL4 promoter. Sun et al. (PMID: 39604370) previously showed MYC binding at the ACSL4 promoter, but it remains unclear whether this is the regulatory element relevant in the context of SPOP mutations. To enhance the novelty and mechanistic depth, the authors should provide a comprehensive ChIP-seq analysis of MYC and CHD1 binding near ACSL4 in both SPOP wild-type and mutant backgrounds. Additionally, SP1 ChIP-seq data should be included in Fig. 7 to support the proposed regulatory model.

Response: Our previous ChIP-seq data indicated that CHD1 binds to ACSL4's promoter region, not the gene body. To avoid confusion, we have revised **Figures 5G** and **5H** to better depict the CHD1 binding site near ACSL4's TSS.

To capture the global binding features of CHD1 in SPOP-mutated PCa cells, we performed CHD1 ChIP-seq in SPOP-WT and SPOP-W131G LNCaP cells. The results showed that, compared to SPOP-WT cells, CHD1-binding peaks across the genome were markedly increased in SPOP-mutated LNCaP cells, and nearly 90% of peaks were localized to the promoters of target genes **(Fig. S5G,H; Line 252)**. It suggests that SPOP mutations promote CHD1's chromatin remodeling function in a more generic manner, implicating that CHD1 contributes to the transcriptomic reprogramming in PCa harboring SPOP mutations. Although we also performed c-Myc ChIP-seq in those cells, the experiments failed, likely due to the bad quality of the c-Myc antibody. Future studies are warranted to evaluate the co-binding features of CHD1 and c-Myc on the global genome in SPOP-mutated PCa.

As suggested, we performed SP1 ChIP-seq in SPOP^{W131G}/CHD1^{KO} LNCaP cells treated with or without atorvastatin. As shown in **Figure S7F-G (Line 335)** and below, the results indicated that cholesterol-lowering drug treatment markedly increased SP1-binding peaks at gene promoters across the genome in SPOP/CHD1 co-deficient PCa LNCaP cells, suggesting a generic effect of cholesterol on the SP1 transcriptome.

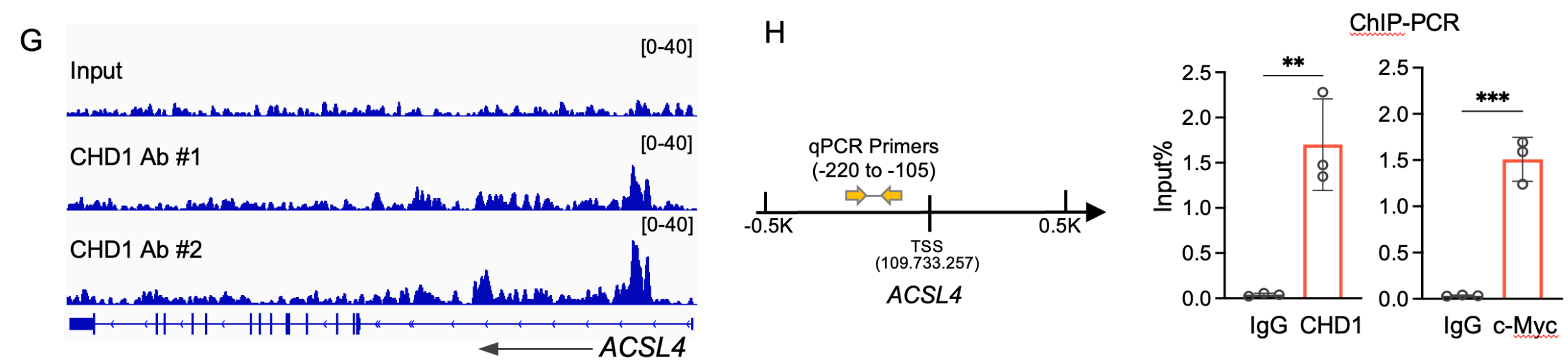

Figure 5G-H, G, ChIP-Seq profile showing the binding of CHD1 to the promoter region of the *Acsl4* gene in murine PCa cells. Two antibodies against CHD1 were used for ChIP-seq. **H,** ChIP-qPCR was performed in SPOP^{W131G} LNCaP cells using IgG control, CHD1, or c-Myc antibodies. The enrichment of CHD1 and c-Myc at the ACSL4 promoter are normalized to IgG control. The 1.5k upstream or downstream of *ACSL4*'s transcription start site (TSS) and the location of primers are shown.

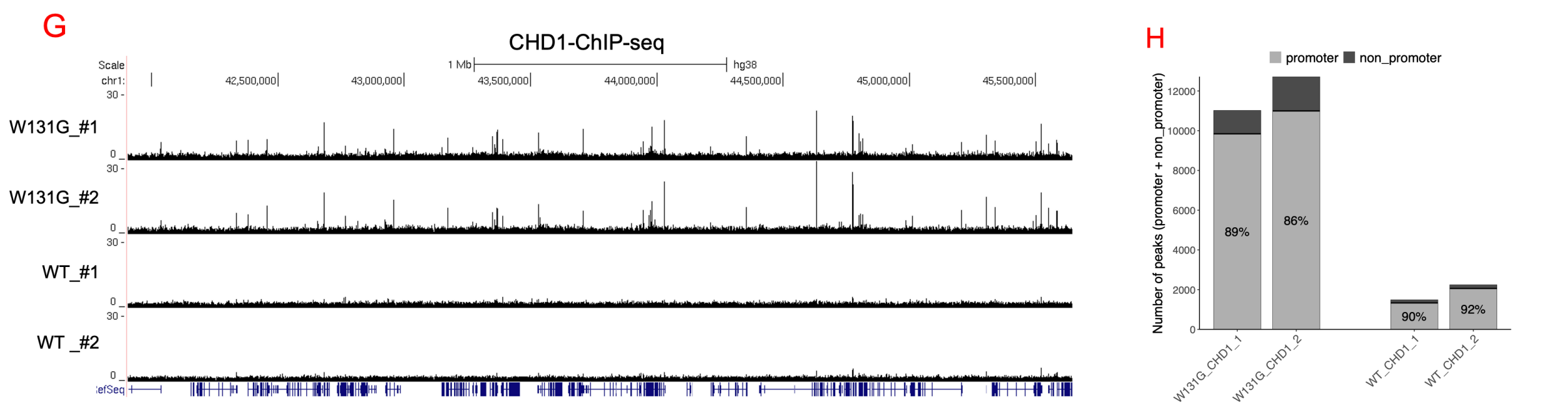

Figure S5G-H, ChIP-seq was performed in WT and SPOP^{W131G} LNCaP cells using IgG control or CHD1 antibodies. Representative landscape of CHD1 in four samples was created using USCS genome browser (**G**). The number of peaks on promoter or none-promoters of CHD1 in WT versus SPOP^{W131G} LNCaP cells are presented (**H**).

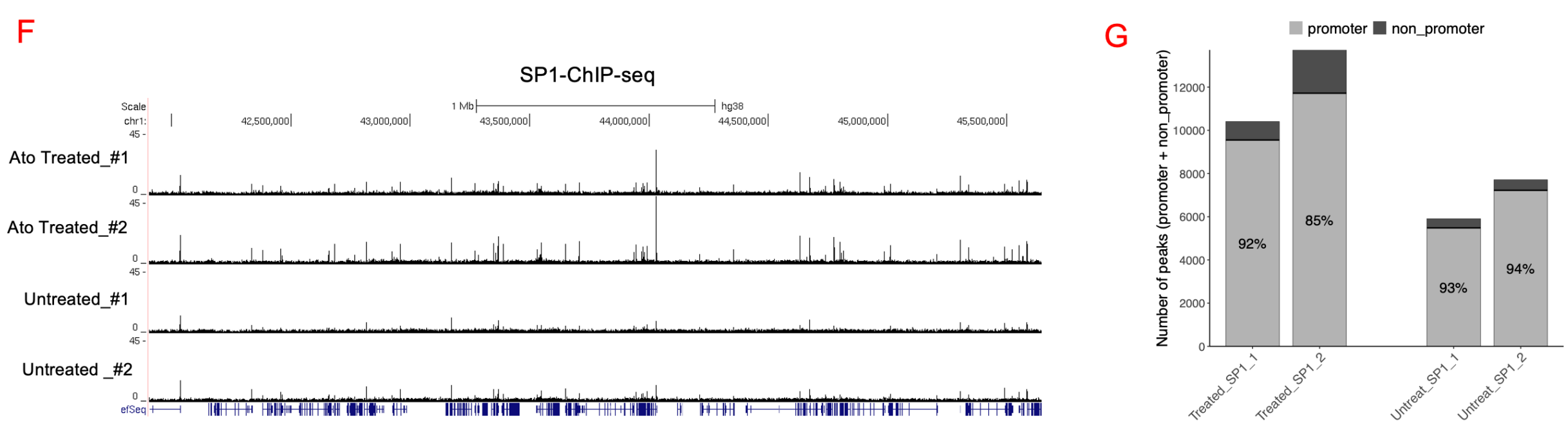

Figure S7F-G, SPOP^{W131G}/CHD1^{KO} LNCaP cells were treated with or without atorvastatin (10 μM) for 48h, followed by ChIP-seq using IgG control or SP1 antibodies. Representative landscape of SP1 in four samples was created using USCS genome browser (**F**). The number of peaks on promoter or none-promoters of SP1 in all samples are presented (**G**).

4. Western blot: Densitometric analysis should be performed for all western blots and reported as numerical values (normalized to control) beneath each image. This would provide a semi-quantitative assessment of the magnitude of changes, such as the increase in ACSL4 following Ato treatment in Fig. 7B.

Response: Densitometric analysis has been performed for all Western blot results. The numerical values of the indicated protein were normalized to the actin control and shown beneath each image. The revised **Fig. 7B** is presented below as an example.

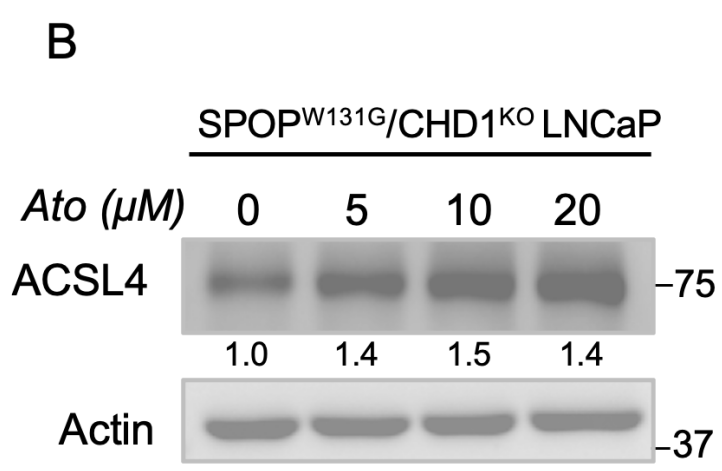

Figure 7B. Western blot analysis of ACSL4 in SPOP^{W131G}/CHD1^{KO} LNCaP cells treated with different doses of atorvastatin for 48 hr. Relative density ratios of indicated proteins/actin are shown.

Reviewer #2 (Remarks to the Author):

The manuscript by Feiyu Chen et al. presents an interesting and well-structured study addressing the genetic determinants of ferroptosis susceptibility in prostate cancer. The authors demonstrate that SPOP mutations and CHD1 deletions define distinct molecular subtypes with increased sensitivity to ferroptosis inducers, highlighting the MYC–ACSL4 axis as a key regulatory pathway. Importantly, the translational potential of the work is underscored by the finding that cholesterol-lowering agents can restore ferroptosis sensitivity in resistant tumors, suggesting novel biomarker-driven therapeutic strategies for advanced prostate cancer. I believe this manuscript has strong potential for publication in Nature Communications, pending clarification of the following points.

Response: We appreciate this reviewer's positive feedback and constructive comments. During the revision period, we generated additional results to address these comments. The manuscript has been significantly improved with these new results. Please find our point-by-point responses below.

Major point:

1. The authors discuss that SPOP mutations increase ACSL4 levels, thereby sensitizing cells to ferroptosis. While this is an interesting finding, a global lipidomics analysis would be essential to provide a broader understanding of which lipid species are altered upon SPOP mutation. Inclusion of such data, or at least a discussion of this point, would strengthen the manuscript.

Response: As suggested, we performed lipidomic profiling in control versus SPOP-mutated LNCaP cells. The results indicated that SPOP mutations promoted PUFA-containing phospholipids in PCa (below; **Figures 2A**, **S2A-B, Supplementary Table 1)**. Our recent Nature Cancer paper reported a lipidomic profiling dataset of control and CHD1-depleted LNCaP cells (PMID: 40360905). Reanalysis of this dataset revealed that CHD1 loss led to a remarkable reduction of PUFA-containing phospholipids **(**below; **Fig. 4E)**, which is opposite to the effects of SPOP mutations. These unbiased, global lipidomic analyses provide essential evidence supporting the vital roles of SPOP and CHD1 in PUFA metabolism and ferroptosis, significantly strengthening the manuscript.

Line 149: *"To elucidate the mechanism of ferroptosis sensitivity in SPOP-mutated cells, we performed lipidomic profiling in LNCaP cells expressing the W131G mutation (**Fig. S2A-B**). The results indicated that SPOP mutation induces widespread accumulation of polyunsaturated fatty acids (PUFAs) across multiple lipid classes and molecular species (**Supplementary Table 1**). The pathway enrichment analysis demonstrates significant elevation of various PUFA-containing phospholipids, with phosphatidylinositols (PI) and phosphatidylethanolamines (PE) showing the most pronounced enrichment, particularly PI (39:4) and PE (39:4) (**Fig. 2A**). This PUFA accumulation extends across different lipid categories, including phosphatidylserines (PS), and overall phosphoglycerides (PG), suggesting a systematic shift in cellular lipid composition (**Fig. 2A**). Notably, individual fatty acid species demonstrate robust enrichment, with C21:4, C18:3, and C22:5 showing significant upregulation in SPOP-mutated cells (**Fig. 2A**). The coordinated elevation of PUFAs with varying chain lengths (C12 to C26) and desaturation levels (3-5 double bonds) indicates enhanced PUFA incorporation into membrane phospholipids rather than simple accumulation of free fatty acids (**Fig. 2A**). These findings suggest that SPOP mutations reprogram the metabolism of PUFA in PCa cells."*

***Line 227**: "In contrast to what was observed following SPOP mutation (**Fig. 2A**), lipidomic profiling analysis revealed that CHD1 knockout led to a remarkable reduction of PUFA-containing phospholipids **(Fig. 4E)**. The results suggest that CHD1 plays a vital role in PUFA lipid metabolism and ferroptosis and contributes to the increased ferroptosis sensitivity in SPOP-mutated PCa."*

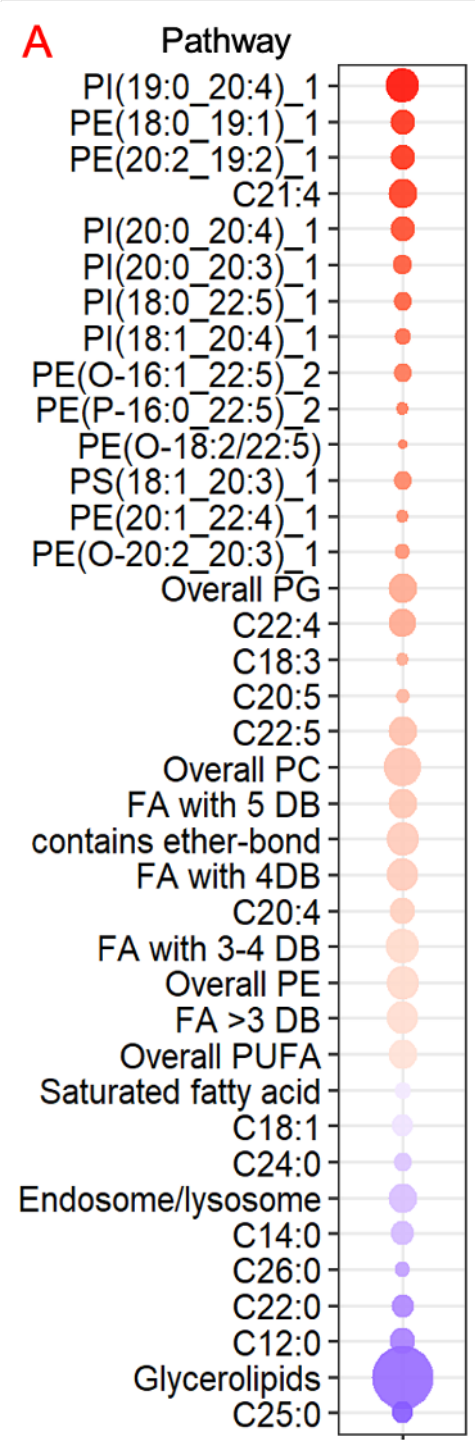

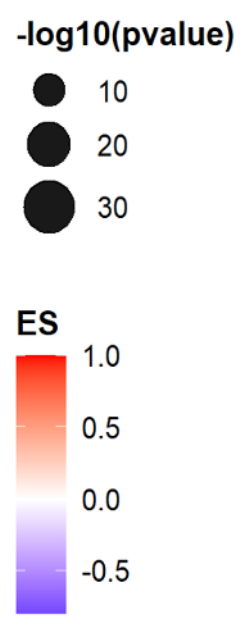

Figure 2A. Lipidomic analysis reveals accumulation of PUFA-containing phospholipids in SPOP^{W131G} LNCaP cells, normalized to control cells; n = 4 biological replicates. Enrichment score (ES) and p values are shown.

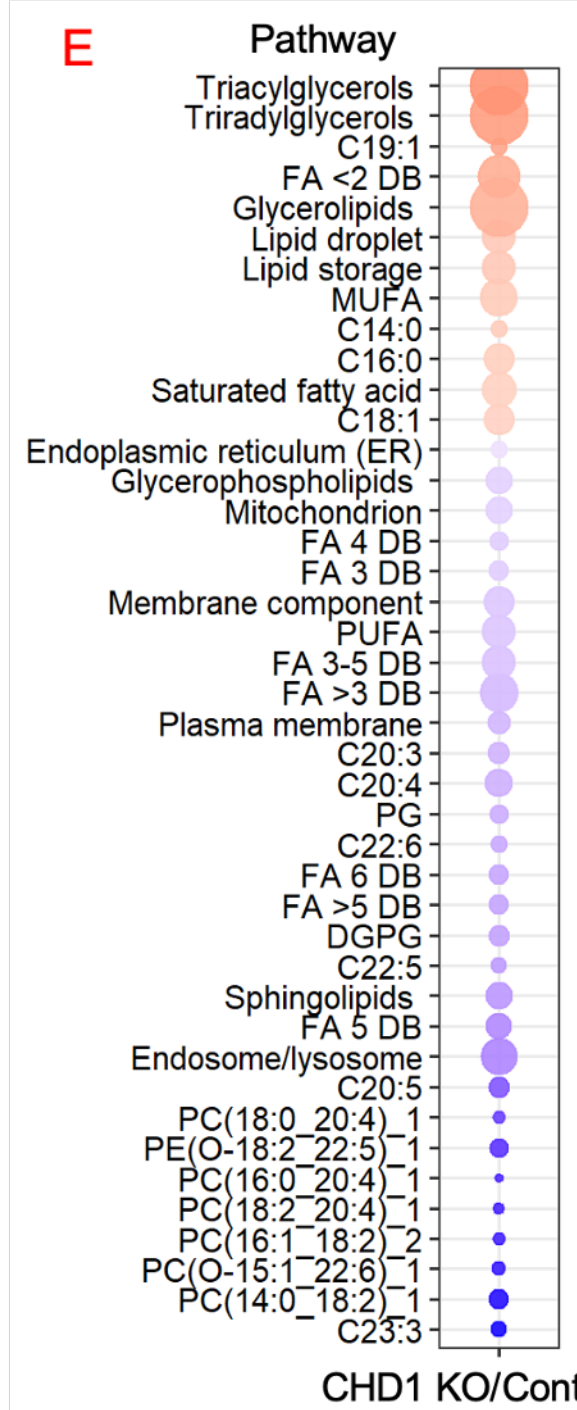

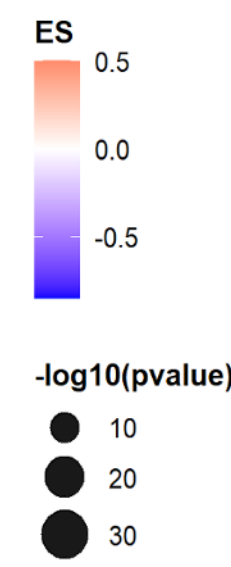

Figure 4E. Lipidomic analysis showing decreased abundance of PUFA-containing phospholipids across multiple lipid classes in CHD1 knockout cells; n = 4 biological replicates.

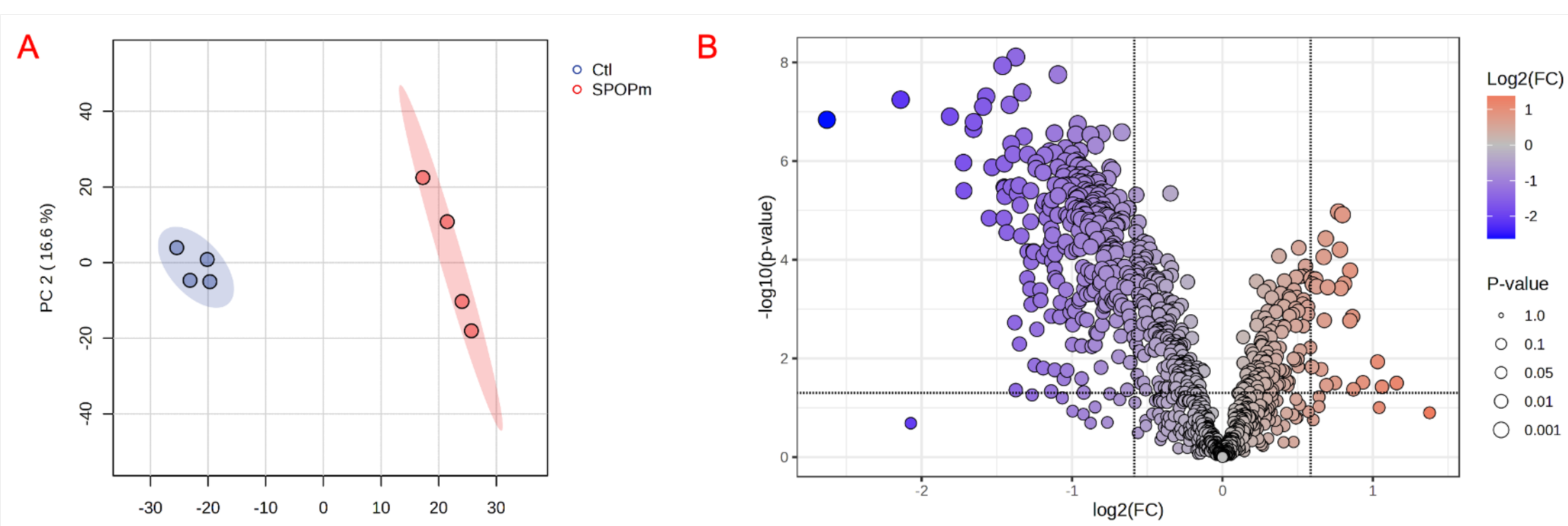

Figure S2. **A,** Lipidomic profiling was performed in control (blue) and SPOP^{W131G} (red) LNCaP cells with 4 replicates using LC–MS/MS. Principal component analysis (PCA) of gene expression profiles showing clear separation between control (blue) and SPOP^{W131G} (red) LNCaP cells. The percentage of variance explained by each principal component is indicated on the axes. **B,** Volcano plot of all lipid species detected in control and SPOP^{W131G} samples. Vertical and horizontal dashed lines denote the cutoffs used for fold change and statistical significance, respectively. P values and Log2(fold-change) are shown.

2. The authors report that c-Myc levels increase upon SPOP mutation. It should be clarified whether overexpression of MYC in SPOP wild-type cells can also increase ferroptosis sensitivity, similar to previous findings showing that MYCN-amplified neuroblastoma cells are sensitized to ferroptosis and rely on selenocysteine uptake and metabolism.

Response: To address this comment, we compared the effects of overexpressing c-Myc and SPOP-W131G mutant in LNCaP cells. The results showed that c-Myc overexpression phenocopies SPOP mutations, rendering LNCaP cells more sensitive to the ferroptosis inducer RSL3 **(Fig. 3G)** and promoting ACSL4 expression (**Fig. S3E) (Line 205)**.

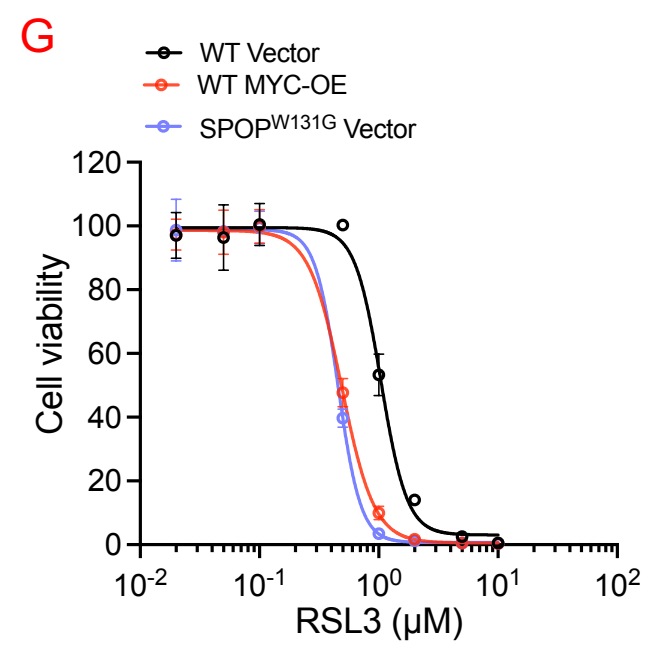

Figure 3G, MYC or SPOP^{W131G} was overexpressed in LNCaP cells, followed by determining the dose-response to RSL3 (24 hr).

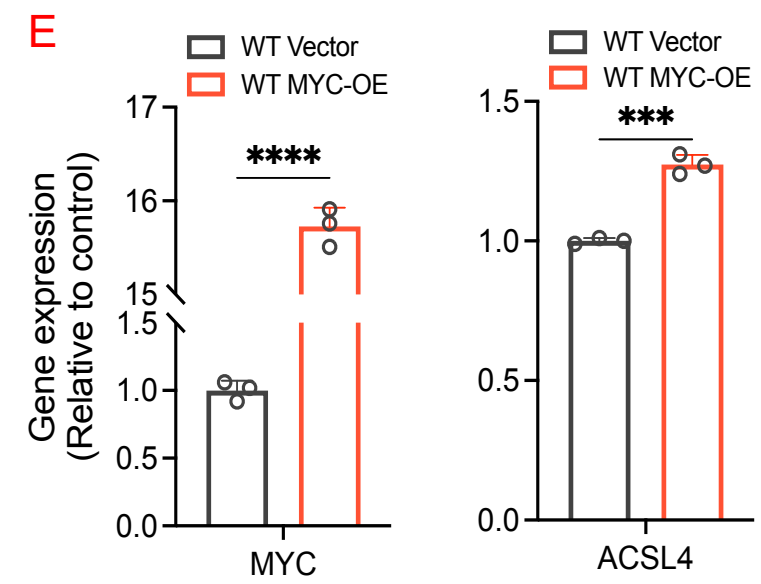

Figure S3E, MYC was overexpressed (OE) in LNCaP cells, followed by qPCR analysis of MYC and ACSL4 mRNA levels.

3. It would be important to assess whether SPOP mutations and the observed c-Myc changes have an impact on other ferroptosis regulators, such as FSP1 (GSH-independent pathway) and SLC7A11, which play key roles in selenium metabolism and glutathione synthesis, as well as on GCLM, ALDH, and other MYC targets. More global molecular experiments, such as proteomics or metabolomics, could help address this, followed by targeted validation focusing on key ferroptosis modulators, including FSP1, SLC7A11, GPX4, LRP8, and PRDX6.

Response: We performed qPCR and Western blot assays to assess the effects of SPOP mutations on other ferroptosis regulators, including FSP1, SLC7A11, GPX4, DHODH, GCH1, and NRF2, but found that SPOP mutations have little impact on these regulators (**Fig. S2C-D; Line 164**). Together with the above lipidomic profiling data (Response to Comment #1), these new results reinforce the role of SPOP in ferroptosis, which is mainly mediated by ACSL4.

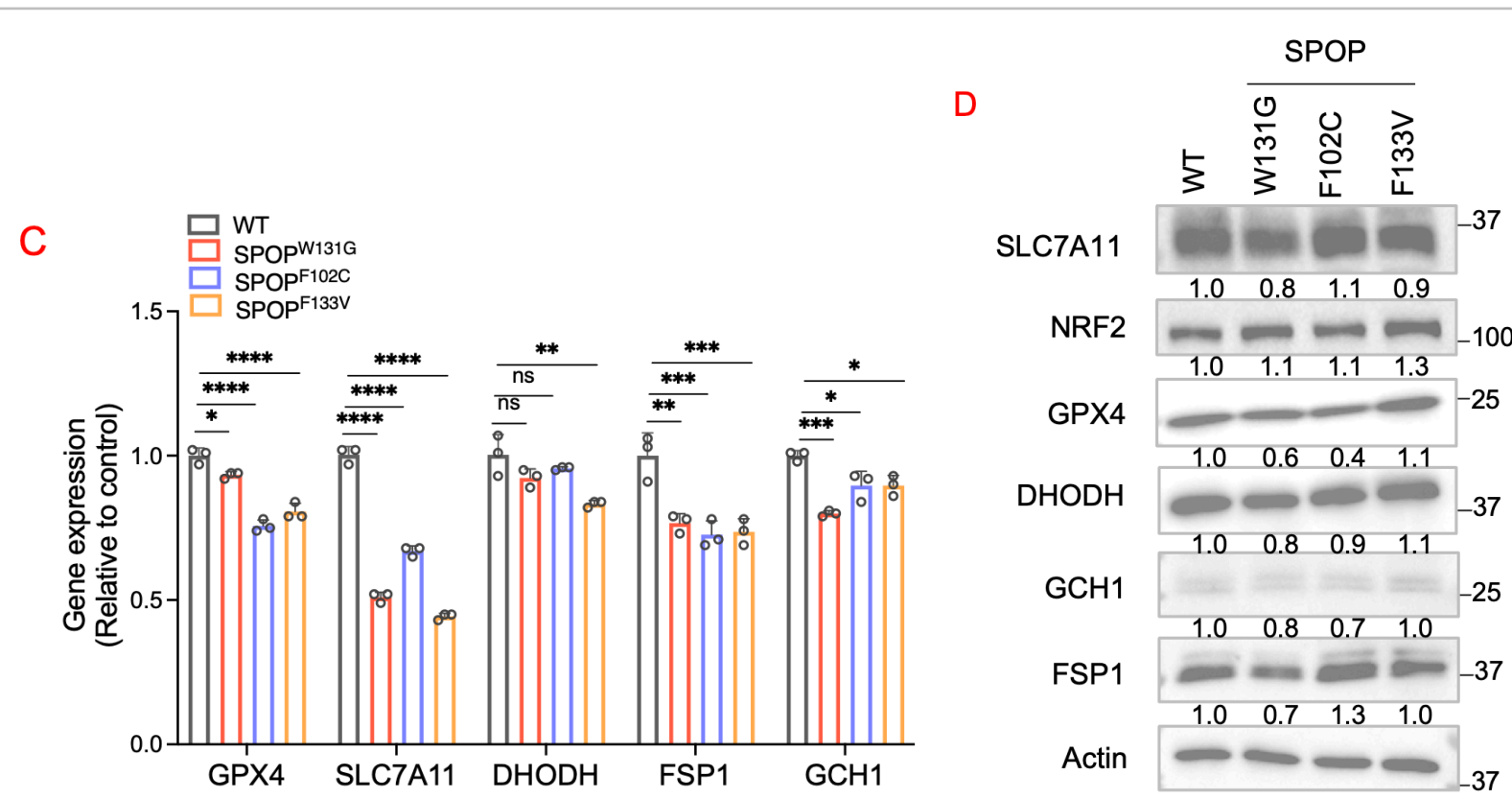

Figure S2C-D, mRNA (**C**) and protein (**D**) levels of indicated ferroptosis regulators in WT or SPOP-mutated (F102C, W131G, F133V) LNCaP cells. Relative density ratios of indicated proteins/actin are shown.

4. The authors should clarify whether the effect of SPOP mutations on ferroptosis is restricted to prostate cancer, or if it extends to other cancer types. Testing additional cancer cell lines would add valuable context.

Response: In addition to prostate cancers, nearly 5-10% of endometrial carcinoma (EC) contain SPOP mutations (PMID: 36512624), and the most frequent mutations are M117V, D140G, E47K, and E50K. To address this comment, we constructed and introduced these mutations into EC cell lines, RL952 and KLE. The results indicated that those EC-associated SPOP mutations had little impact on ferroptosis sensitivity or ACSL4 expression, as shown below. It suggests that the effects of SPOP mutations on ferroptosis may be restricted to prostate cancer.

Prior studies identified BRD2/3/4 proteins (BETs) as SPOP-CUL3 substrates in both EC and prostate cancer (PCa). EC-associated SPOP mutants (E50K and R121Q) preferentially degrade BETs (PMID: 28805821) and sensitize EC cells to BET inhibitors (PMID: 28805821); In contrast, PCa-specific SPOP mutations resulted in the accumulation of BETs and promoted PCa cells resistant to BET inhibitors (PMID: 28805821). These observations, along with our data, imply that distinct mechanisms of action may be associated with SPOP mutations that occurred in PCa and EC. Even though mutants at different residues are located within the same domain, they may induce distinct conformational changes in SPOP proteins, leading to opposite functional consequences. This point has been discussed in the revised manuscript.

Line 457: "Given the high frequency and clinical impact of SPOP mutations and CHD1 deletion in PCa, our studies primarily focused on PCa models. Although 5-10% of endometrial carcinomas (EC) harbor SPOP mutations[57], we didn't observe EC-associated SPOP mutations (M117V, D140G, E47K, and E50K) affecting ACSL4 induction or ferroptosis sensitivity in EC cells, suggesting that their effects and mechanisms of action may be context-dependent."

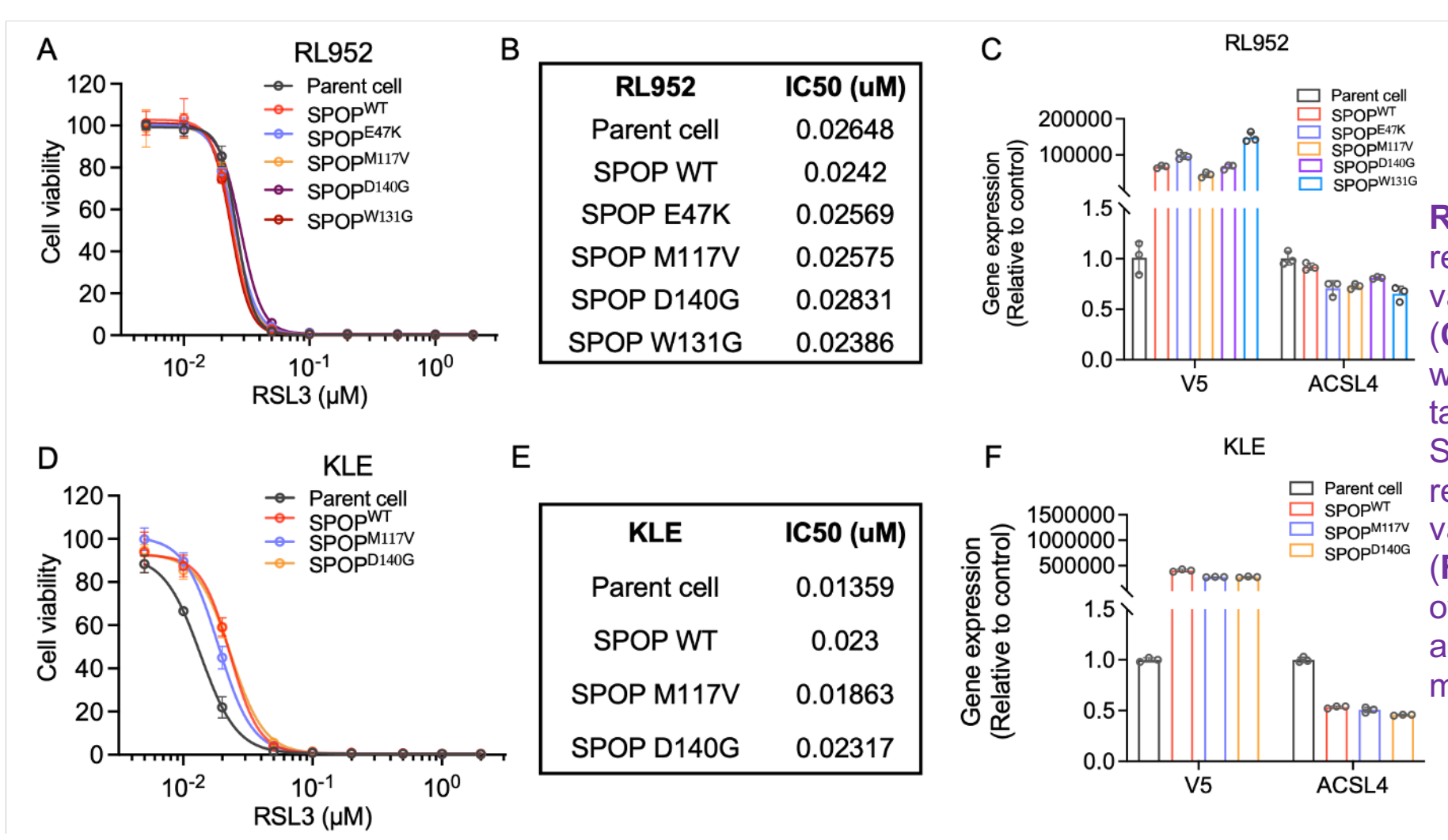

RL952	IC50 (uM)
Parent cell	0.02648
SPOP WT	0.0242
SPOP E47K	0.02569
SPOP M117V	0.02575
SPOP D140G	0.02831
SPOP W131G	0.02386

KLE	IC50 (uM)
Parent cell	0.01359
SPOP WT	0.023
SPOP M117V	0.01863
SPOP D140G	0.02317

Rebuttal Figure 1. **A-C,** Dose-response curves of RSL3 (**A**), IC50 values (**B**), and ACSL4 expression (**C**) in EC cell line RL952 with or without overexpression of V5-tagged WT and EC-associated SPOP mutations. **D-F,** Dose-response curves of RSL3 (**D**), IC50 values (**E**), and ACSL4 expression (**F**) in EC cell line KLE with or without overexpression of V5-tagged WT and EC-associated SPOP mutations.

- Minor points:

1. In Figure 1, the authors show that SPOP mutations sensitize cells to RSL3. It is important to explain the rationale for using relatively high concentrations of RSL3 in the μM range, since most cancer cell lines are highly sensitive to RSL3 at much lower (nM) doses. The authors should clarify why such high doses were chosen for their experimental setup. Testing other classes of ferroptosis-inducing compounds, such as erastin, could help strengthen this observation.

Response: Prior studies reported the sensitivities of diverse PCa cell lines to RSL3 (PMID: 33483372). Compared to AR-negative cell lines DU145, PC3, and H660, AR-positive cell lines LNCaP, 22Rv1, and ARCaP exhibited higher IC50s of RSL3 for unclear reasons (**Rebuttal Figure 2**). Given the important roles of SPOP mutations in AR-positive PCa, we primarily used LNCaP throughout the study and thus chose high doses for the experimental setup.

As suggested, we tested the sensitivity of SPOP-mutated PCa cells to another class of ferroptosis-inducing compounds, erastin and IKE. The results showed that SPOP mutations had little impact on their sensitivities (**Rebuttal Figure 3**), consistent with unchanged SLC7A11 expression levels in this context (Refer to Response to Comment #3).

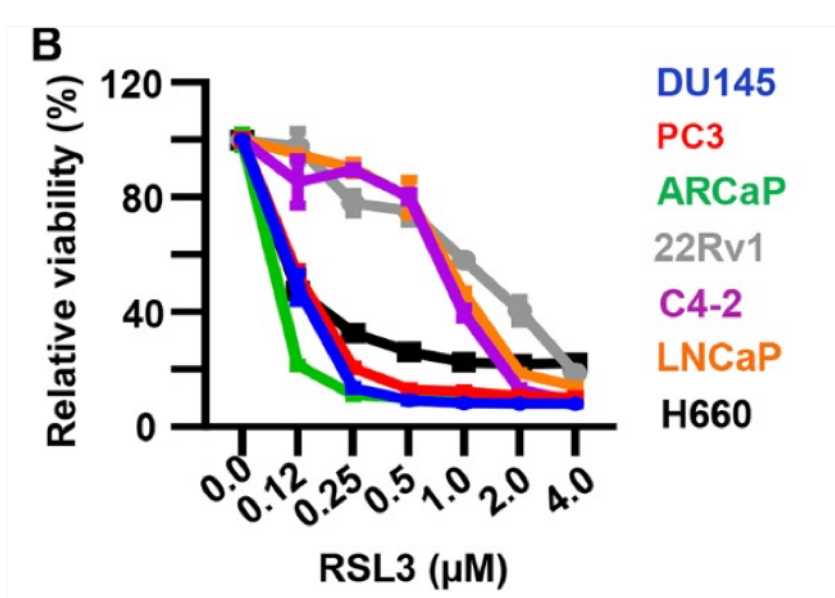

Rebuttal Figure 2. Dose-response curves of RSL3 in diverse PCa cell lines (data cited from PMID: 33483372).

Treatment	Prostate cancer cell	IC50 (uM)
Erastin	LNCaP WT	18.32
	LNCaP SPOP^{W131G}	13.73
IKE	LNCaP WT	14.56
	LNCaP SPOP^{W131G}	31.55

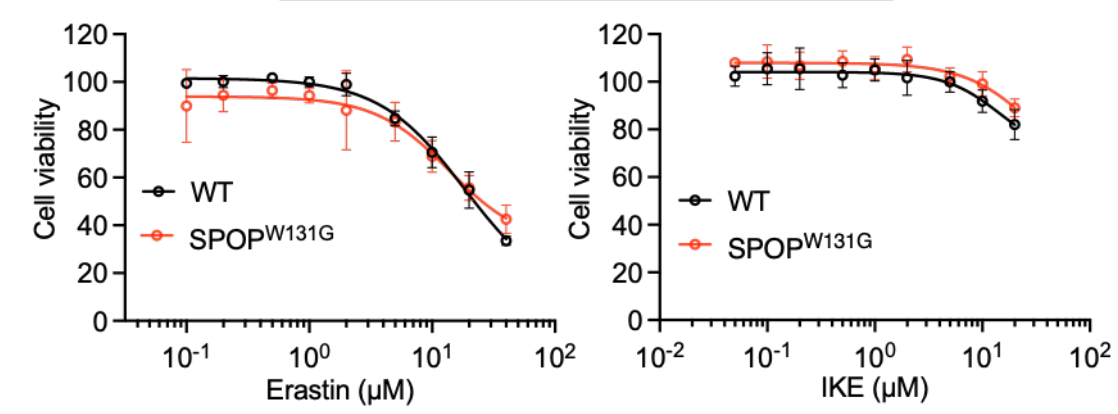

Rebuttal Figure 3. Dose-response curves of Erastin and IKE in WT versus SPOP^{W131G} LNCaP cells.

2. In Figure 6a, it is not clear how many mice were used in each experimental group. The authors should provide the number of animals per group to ensure clarity and reproducibility.

Response: The number of tumors has been added to Fig 6A and all animal experiments.

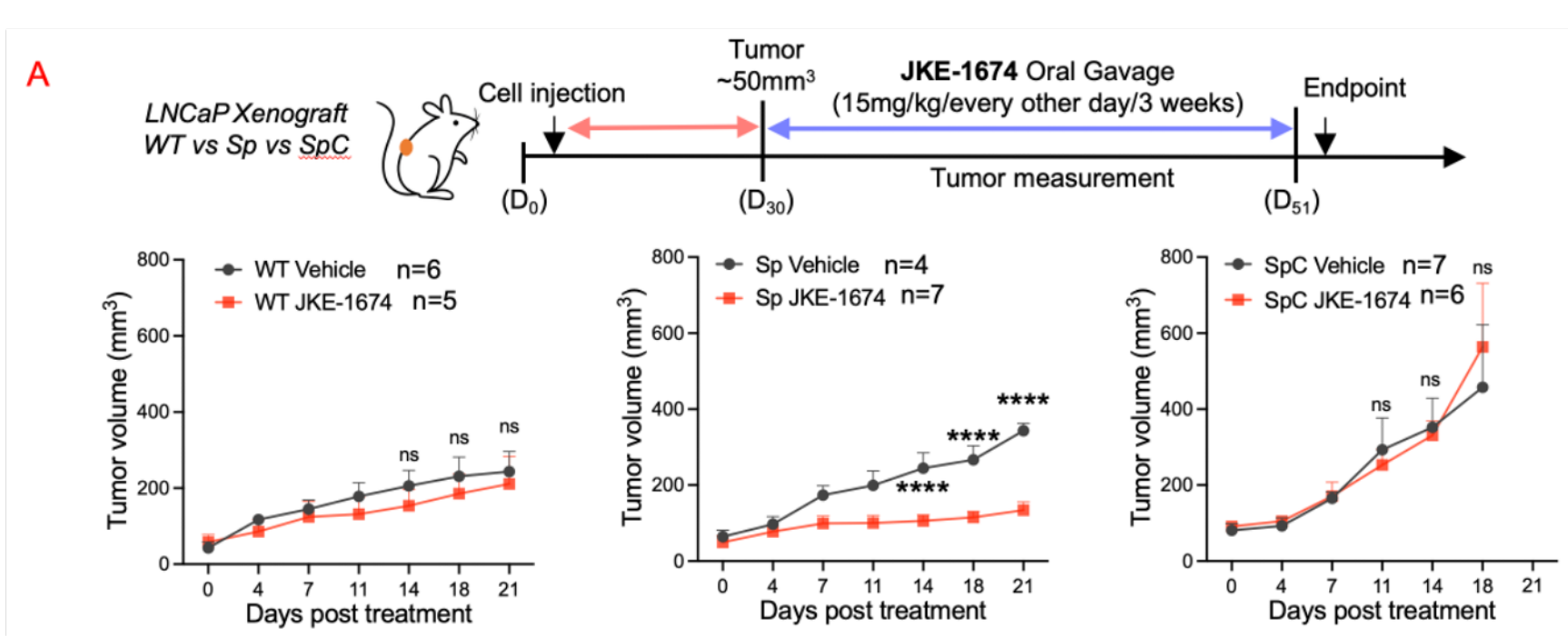

Figure 6A, Schematic of in vivo study design using wildtype (WT), SPOP^{W131G} (Sp), SPOP^{W131G}/CHD1^{KO} (SpC) LNCaP xenograft models. The number of tumors in each group is presented.

Reviewer #3 (Remarks to the Author):

This manuscript by Chen et al. investigates the important roles of SPOP mutations and CHD1 deletions in regulating ferroptosis susceptibility in prostate cancer. The authors propose a mechanistic model whereby SPOP mutations stabilize c-MYC, which in cooperation with CHD1 activates ACSL4 transcription, thereby sensitizing tumors to ferroptosis. Conversely, CHD1 loss abrogates this process, conferring ferroptosis resistance. Importantly, the authors demonstrate that cholesterol-lowering agents (statins) restore ACSL4 expression via a CHD1-independent cholesterol–SP1–ACSL4 axis, thereby re-sensitizing engineered cell lines, GEMMs, organoids, and xenografts. Overall, this is a timely and impactful study that addresses an unmet need of identifying genetic biomarkers that dictate ferroptosis susceptibility. The mechanistic depth, complementary use of models, and translational perspective make this work significant.

Response: We thank this reviewer for his/her positive feedback and constructive comments. During the revision period, we generated new data, revised the figures, and clarified some descriptions, as suggested. The manuscript has been significantly improved after addressing these comments. Please find our point-by-point responses below.

Major Concerns

1. While the ChIP experiments convincingly show that CHD1 facilitates c-MYC binding to the ACSL4 promoter, the broader chromatin remodeling function of CHD1 in this context is less directly addressed. Techniques such as ATAC-seq or CUT&RUN profiling would provide deeper resolution of how CHD1 loss alters global chromatin accessibility, thereby strengthening the mechanistic framework.

Response: To assess the broader chromatin remodeling function of CHD1 in SPOP-mutated PCa, we performed CHD1 ChIP-sequencing in SPOP-WT and SPOP-W131G LNCaP cells. The results showed that, compared to SPOP-WT cells, CHD1-binding peaks across the genome were markedly increased in SPOP-mutated LNCaP cells, and nearly 90% of peaks were localized to the promoters of target genes **(Fig. S5G,H; Line 252)**. It suggests that SPOP mutations promote CHD1's chromatin remodeling function in a more generic manner, implicating that CHD1 contributes to the transcriptomic reprogramming in PCa harboring SPOP mutations.

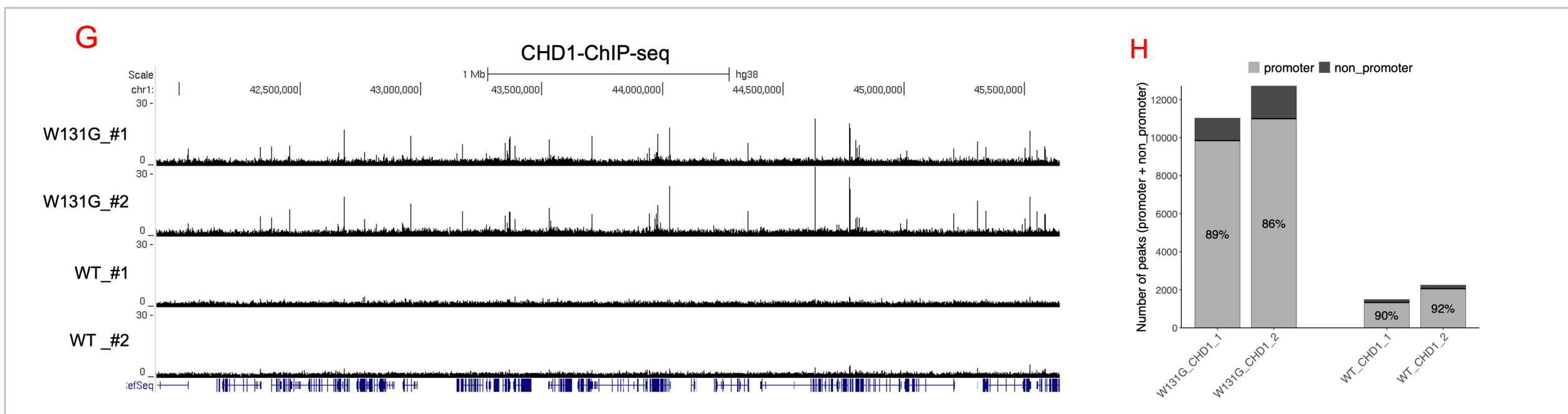

Figure S5G-H, ChIP-seq was performed in WT and $SPOP^{W131G}$ LNCaP cells using IgG control or CHD1 antibodies. Representative landscape of CHD1 in four samples was created using USCS genome browser (**G**). The number of peaks on promoter or none-promoters of CHD1 in WT versus $SPOP^{W131G}$ LNCaP cells are presented (**H**).

2. The identification of a CHD1-independent cholesterol–SP1–ACSL4 pathway is intriguing but remains less deeply explored compared to the SPOP–CHD1–MYC axis. The authors should provide direct evidence that SP1 binds to the ACSL4 promoter under cholesterol-depleted conditions. In addition, the mechanism linking cholesterol depletion to SP1 upregulation remains unclear. Does cholesterol loss affect SP1 protein stability, nuclear localization, or post-translational modification? Clarifying this regulatory step would significantly strengthen the proposed compensatory pathway.

Response: As suggested, we performed SP1 ChIP-qPCR in SPOP^{W131G}/CHD1^{KO} LNCaP cells treated with or without atorvastatin and found that SP1 directly binds to the ACSL4 promoter, and this binding was enriched in the presence of cholesterol-lowering drugs **(Fig. 7D; Line 325)**. To verify if cholesterol-lowering drugs regulate SP1 at the protein level, we first assessed SP1 protein stability using CHX. The results indicated that SP1 is a relatively stable protein with a half-life of > 8 hours in SPOP^{W131G}/CHD1^{KO} PCa cells, regardless of atorvastatin treatment **(Fig. S7B; Line 329)**. Next, we performed SP1 IHC staining on SPOP^{W131G}/CHD1^{KO} xenograft tumors. Atorvastatin treatment elevated total SP1 levels but had little impact on its nuclear localization in vivo **(Fig. S8B-C; Line 362)**. Prior studies reported that phosphorylation regulates SP1 protein stability and nuclear translocation. As suggested, we determined SP1 phosphorylation in SPOP^{W131G}/CHD1^{KO} LNCaP cells and found that atorvastatin treatment did not affect SP1 phosphorylation **(Fig. S7C; Line 329)**. Together with our previous studies at the mRNA levels, these new results indicated that cholesterol-lowering drugs induce SP1 expression by activating its transcription.

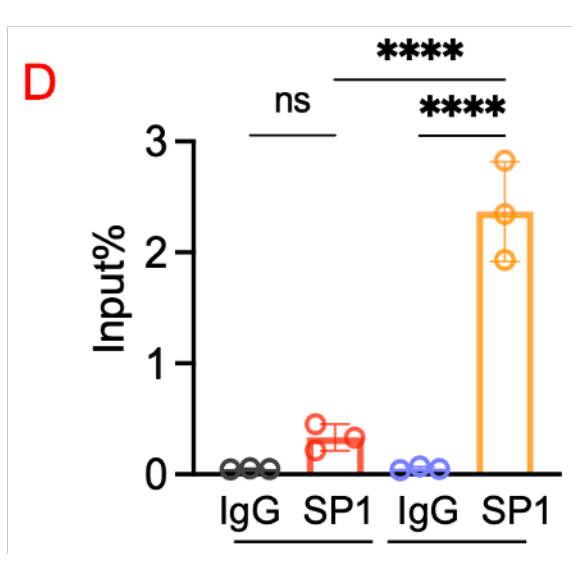

Figure 7D, SPOP^{W131G}/CHD1^{KO} LNCaP cells were treated with or without atorvastatin (10 μM) for 48h, followed by ChIP-qPCR using IgG control or SP1 antibodies. The enrichment of SP1 at the ACSL4 promoter is normalized to IgG control.

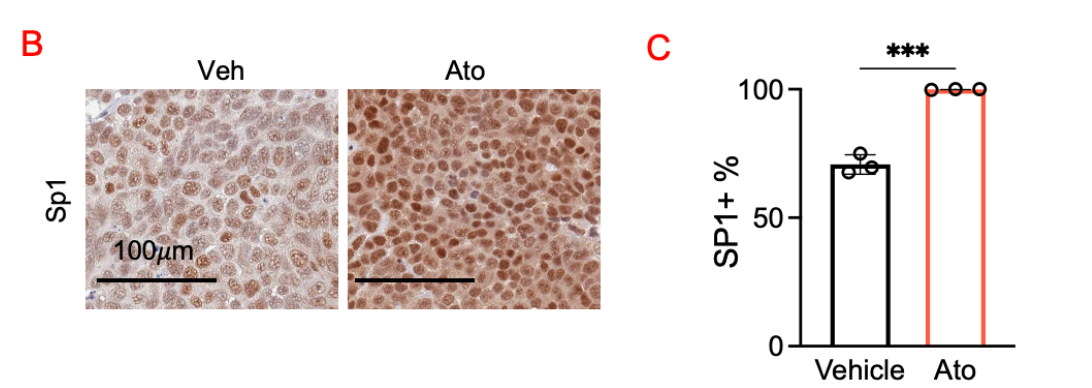

Figure S8B-C, IHC staining (**B**) and quantification (**C**) of SP1 in SpC xenograft tumors with or without atorvastatin treatment. Scale bar, 100 µm.

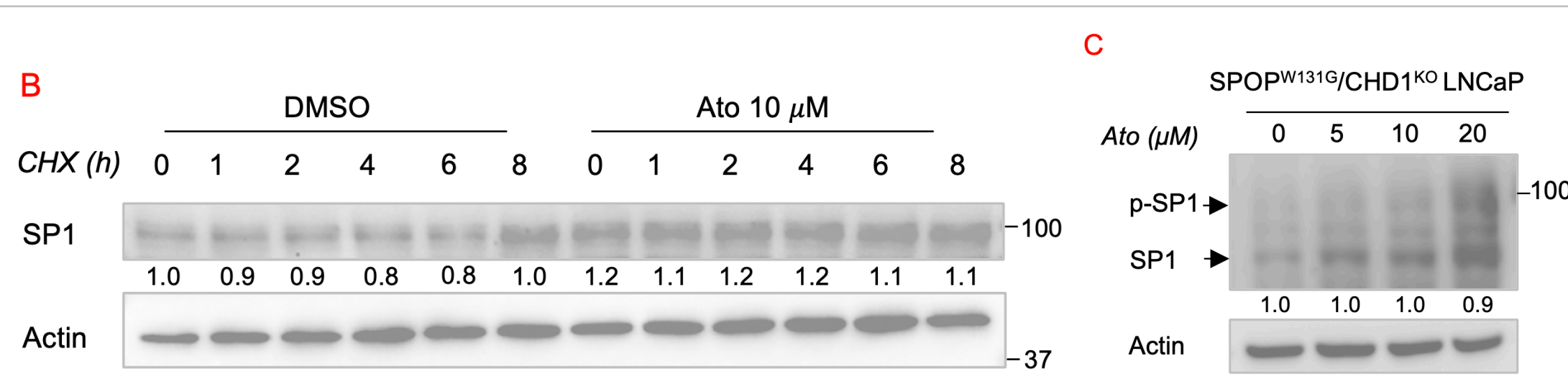

Figure S7, B, SPOP^{W131G}/CHD1^{KO} LNCaP cells treated with 48h atorvastatin (10 μM), followed by the treatment of cycloheximide (CHX, 100 µg/ml) for the indicated times (0, 1, 2, 4, 6 and 8 hr). Protein stability of SP1 was determined by Western blot analysis. Relative density ratios of indicated proteins/actin are shown. **C,** Western blot analysis of phosphorylated SP1 (p-SP1) and unphosphorylated SP1 in SPOP^{W131G}/CHD1^{KO} LNCaP cells treated with different doses of Atorvastatin. Relative density ratios of indicated p-SP1/SP1 are shown.

3. While ACSL4 expression correlations with SPOP/CHD1 alterations are shown using TCGA, additional clinical analyses would enhance the translational impact. Specifically (1) The authors should explicitly state the number of TCGA cases analyzed in the figure legends to ensure transparency. (2) Independent validation cohorts (SU2C or MSK-IMPACT) would strengthen the robustness of the correlations. (3) It would be informative to test whether ACSL4 expression correlates with clinical outcomes (survival or therapy response) in SPOP-mutant versus CHD1-deficient tumors.

Response: As suggested, we indicated the number of TCGA cases analyzed in the figure and legend (**Fig. S5D**). In addition to the TCGA dataset, we also observed that ACSL4 high expression showed a positive correlation trend with SPOP mutations and CHD1 mRNA expression in SU2C datasets (**Rebuttal Figure 4**). However, due to a smaller sample size, no statistical significance was demonstrated regarding these correlations or patient survival in publicly available datasets.

To strengthen clinical relevance and enhance translational impact, we assessed ACSL4 expression at both the mRNA and protein levels in several PCa PDX models harboring SPOP mutations or CHD1 deletions. Compared to PDX-274-2 with intact SPOP and CHD1, the SPOP-mutated PDX-316-2 tumor exhibited a marked elevation of ACSL4 expression, whereas the CHD1-deleted PDX-133-4 tumor expressed a lower level of ACSL4 **(Fig. 5L,M, and S5J; Line 266).** Together with our studies in PCa cell lines, xenograft tumors, GEMMs, and organoids, these new data in PCa PDX models provide additional evidence to support that SPOP mutations and CHD1 deletion play distinct roles in regulating ACSL4.

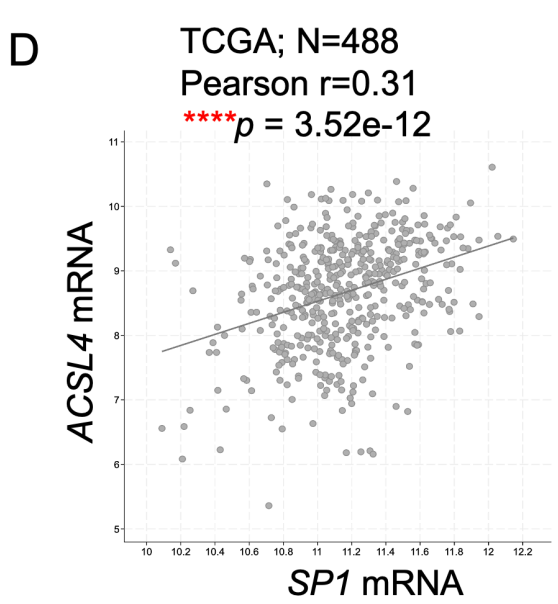

Figure S5D, Scatter plots illustrating the correlations between CHD1 and ACSL4 mRNA levels in human PCa samples (TCGA; n=488). The Pearson correlation coefficient (r) and *P* value are reported.

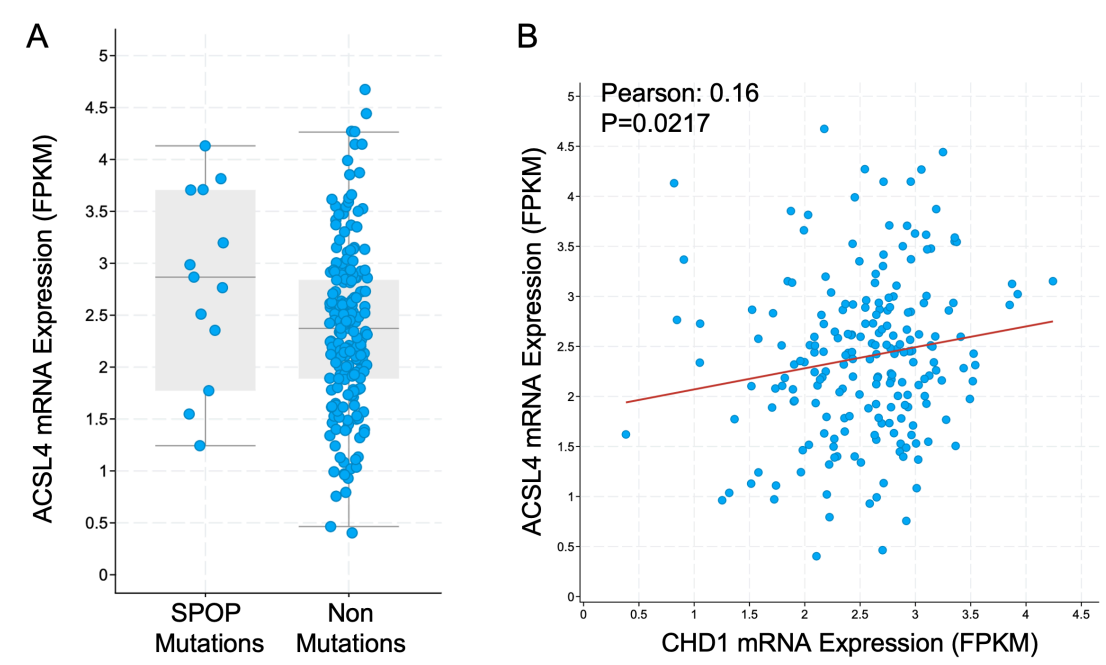

Rebuttal Figure 4, A, ACSL4 mRNA expression in prostate tumors with or without SPOP mutations in the SU2C dataset; **B,** Correlations between CHD1 and ACSL4 mRNA levels in the SU2C PCa dataset.

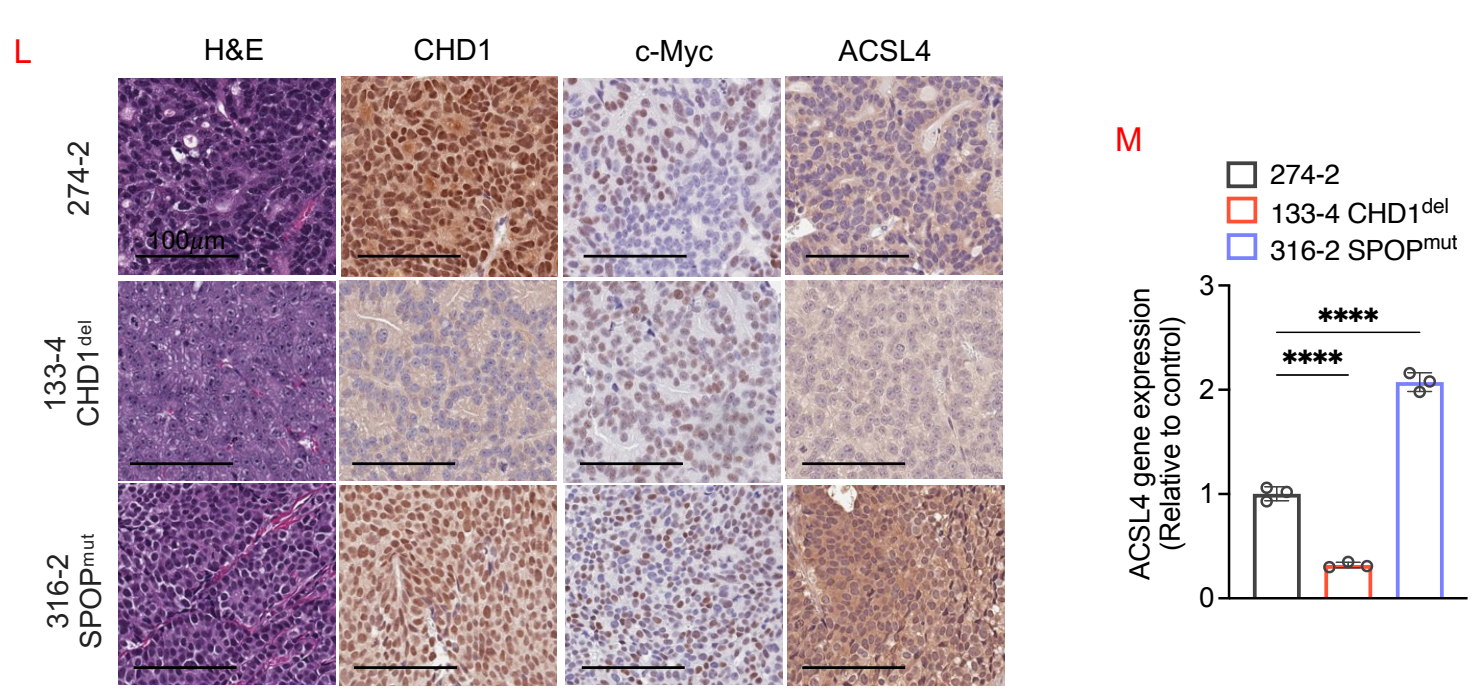

Figure 5. L, Representative H&E and IHC staining of CHD1, c-Myc, and ACSL4 in three prostate cancer patient-derived xenograft (PDX) tumors: 274-2 (intact SPOP/CHD1), 133-4 ($CHD1^{del}$), and 316-2 ($SPOP^{mut}$). Scale bar, 100 µm. **M,** qPCR analysis of ACSL4 mRNA expression in above PDX tumors.

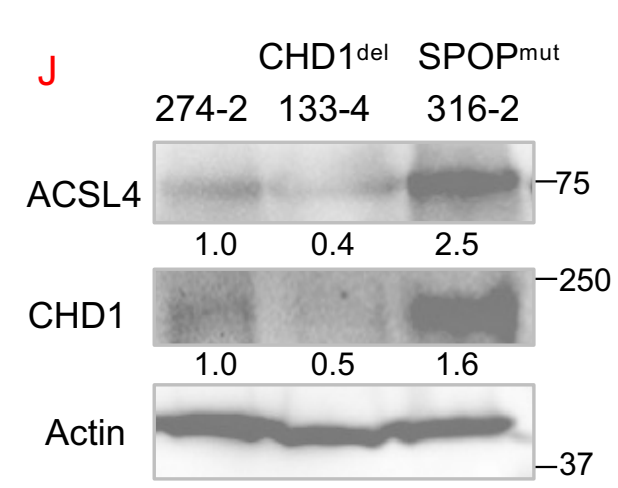

Figure S5J. Western blot analyses of CHD1 and ACSL4 in PCa patient-derived xenograft (PDX) tumors: 274-2 (intact SPOP/CHD1), 133-4 ($CHD1^{del}$), and 316-2 ($SPOP^{mut}$). Relative density ratios of indicated proteins/actin are shown.

4. The authors conclude that SPOP mutations sensitize to ferroptosis in an androgen-independent manner. Given prior evidence that AR signaling regulates ferroptosis-related enzymes, a systematic analysis of AR activity under ferroptosis-inducing conditions would clarify whether AR activity modulates the observed phenotypes. Testing the combination of a GPX4 inhibitor with a potent AR antagonist in SPOP-mutated, CHD1-intact models would also provide a complete therapeutic roadmap.

Response: To address this comment, we first treated SPOP^{W131G} LNCaP cells with RSL3 at varying doses for 24 hours and performed qPCR to assess AR target gene expression. As shown below (**Rebuttal Figure 5**), ferroptosis-inducer treatment has little impact on the transcription of AR target genes. Furthermore, we performed AR IHC staining on SPOP-mutated LNCaP xenograft tumors treated with JKE. The results indicated that ferroptosis-inducing treatment had no effect on AR expression or nuclear localization (**Rebuttal Figure 6**). These data suggest that AR transcriptional activity was not affected under ferroptosis-inducing conditions.

As suggested, we tested the combination of RSL3 with the AR antagonist, enzalutamide, in SPOP^{W131G} LNCaP cells. Dose-response matrix and drug interaction landscapes revealed the synergistic effects between RSL3 and enzalutamide on SPOP-mutated/CHD1-intact PCa cells (**Fig. S8E; Line 428**). This synergy was also discussed in the revised manuscript as a potent therapeutic roadmap in PCa containing SPOP mutations.

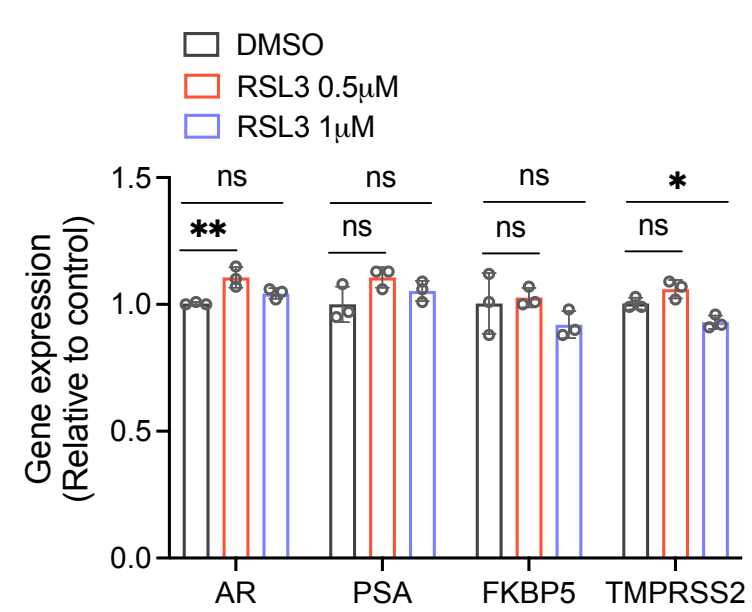

Rebuttal Figure 5. AR target gene expression following different doses of RSL3 treatment for 24 h in SPOP^{W131G} LNCaP cells.

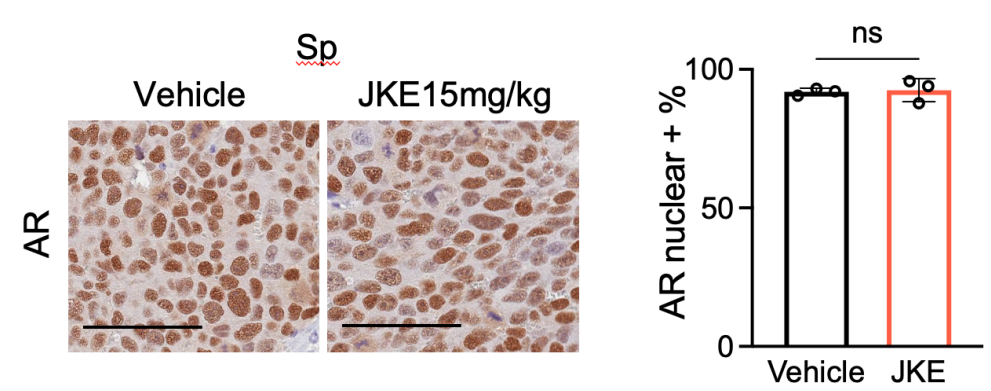

Rebuttal Figure 6. AR nuclear localization in Sp tumors (SPOP^{W131G} LNCaP xenograft tumors) with or without JKE-1674 treatment. Scale bar, 100 μm.

E

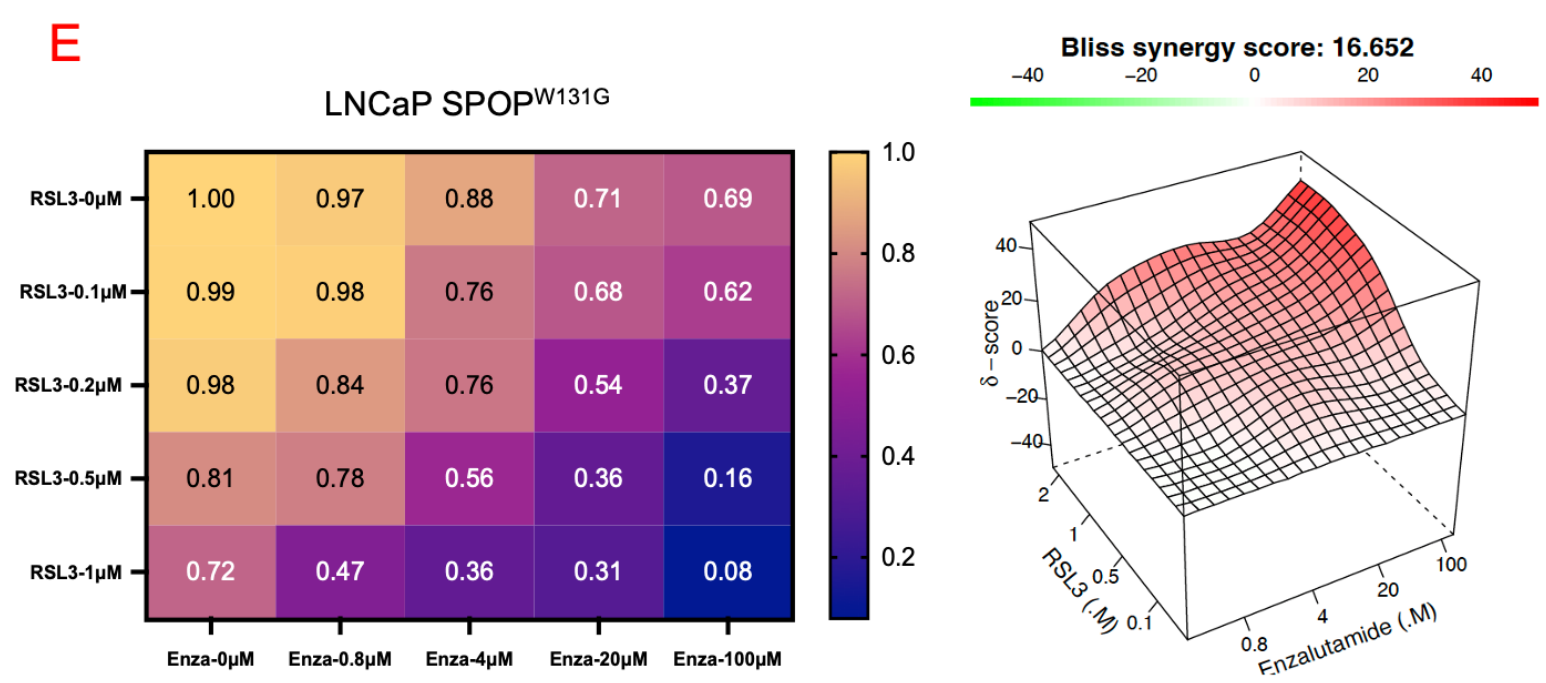

Figure S8. E, Dose-response matrix (left) and drug interaction landscapes (right) revealing synergistic effects of RSL3 and enzalutamide on SPOP^{W131G} LNCaP cells. Drug interaction landscapes and synergy scores were generated using SynergyFinder.

5. Although the mechanistic link between CHD1 loss and ACSL4 silencing is well supported in models, protein-level validation in independent human PCa tumor samples would provide important clinical confirmation that CHD1 deletion consistently associates with reduced ACSL4 expression.

Response: To strengthen the clinical relevance between CHD1 loss and ACSL4 silencing, we assessed ACSL4 expression at the mRNA and protein levels in PCa PDX models with or without CHD1 deletions. Compared to the PDX-274-2 tumor with intact CHD1, the CHD1-deleted PDX-133-4 tumor expressed a lower level of ACSL4 expression **(Fig. 5L,M, and S5J; Line 266).** Together with our studies in PCa cell lines, xenograft tumors, GEMMs, and organoids, these new data in PCa PDX models provide important clinical confirmation that CHD1 deletion consistently associates with reduced ACSL4 expression.

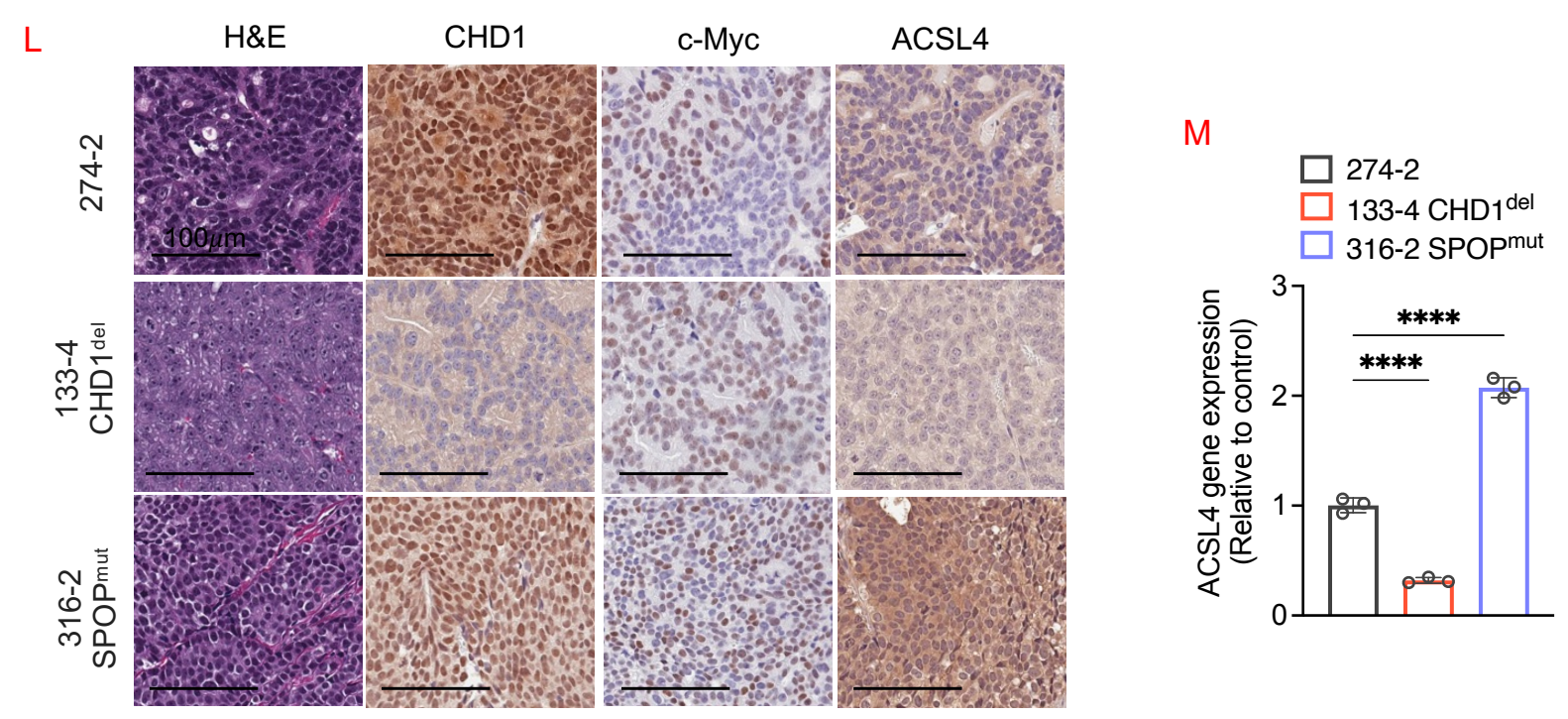

Figure 5. L, Representative H&E and IHC staining of CHD1, c-Myc, and ACSL4 in three prostate cancer patient-derived xenograft (PDX) tumors: 274-2 (intact SPOP/CHD1), 133-4 (CHD1^{del}), and 316-2 (SPOP^{mut}). Scale bar, 100 µm. **M,** qPCR analysis of ACSL4 mRNA expression in above PDX tumors.

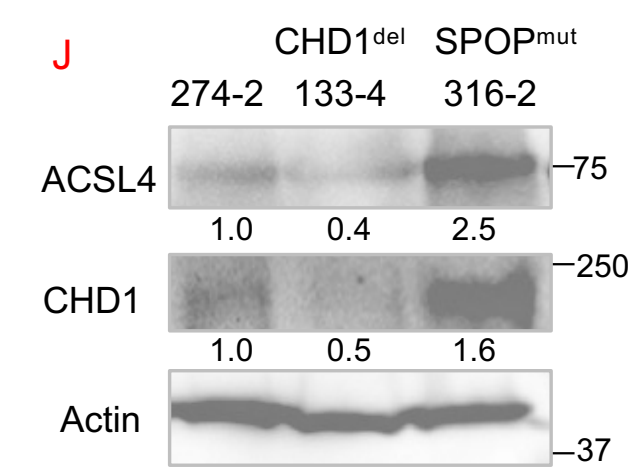

Figure S5J. Western blot analyses of CHD1 and ACSL4 in PCa patient-derived xenograft (PDX) tumors: 274-2 (intact SPOP/CHD1), 133-4 (CHD1^{del}), and 316-2 (SPOP^{mut}). Relative density ratios of indicated proteins/actin are shown.

6. The proposed statin and GPX4 inhibitor strategy is compelling. A powerful complementary analysis would investigate whether PCa patients with SPOP-mut/CHD1-deficient tumors who were incidentally prescribed statins in the clinic had improved outcomes compared to those who were not. Furthermore, since SPOP/CHD1-deficient tumors are aggressive and prone to lineage plasticity, the authors should examine whether long-term treatment of xenografts or GEMMs induces markers of lineage switching (NEPC or basal differentiation), which would indicate potential escape routes from ferroptosis-inducing pressure.

Response: We appreciate this reviewer highlighting the translational significance of combining statins with ferroptosis inducers in SPOP-mut/CHD1-deficient tumors. Due to limited access to data from statin clinical trials, we were unable to investigate correlations between statin prescription and clinical outcomes in PCa patients. Alternatively, we assessed the efficacy of this combination (JKE-1674 and atorvastatin) in our SPOP-mut/CHD1-deficient GEMMs, which better recapitulate the disease progression and therapy response of prostate tumors in patients. Consistent with our observations in xenograft models, the new preclinical studies in spontaneous GEMMs demonstrate the therapeutic potential of combining statin with ferroptosis-inducing drugs in SPOP-mut/CHD1-deficient PCa (below; **Fig. 8D-G; Line 364**). Histopathology analysis revealed that the combination treatment led to a marked reduction in cell proliferation, as assessed by Ki67, but had little impact on lineage plasticity, neither NEPC nor basal differentiation (below; **Fig. S8D; Line 367**).

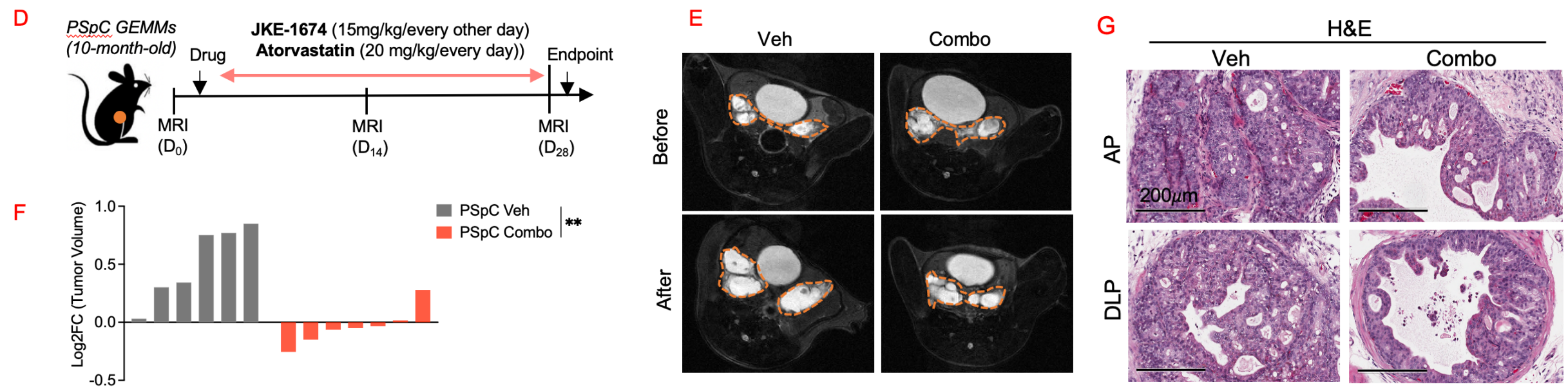

Figure 8. D, Schematic of combination treatment study design in GEMMs. 10-month-old PSpC GEMM mice were imaged by 7T-MRI to confirm tumor formation, followed by oral administration of JKE-1674 (15 mg/kg, every other day) and atorvastatin (20 mg/kg/day) for 4 weeks. Tumor progression was monitored by biweekly MRI imaging. **E**, Representative MRI images of PSpC tumors before and after vehicle (Veh) or combination treatment (Combo). Tumor areas are indicated by dashed outlines. **F,** Waterfall plot of log2 fold change in tumor volume for individual PSpC tumors in the Veh (n=6) and Combo (n=7) groups. **G,** Representative histological staining of PSpC tumors from the anterior prostate (AP) and dorsolateral prostate (DLP) lobes treated with Veh or Combo. Scale bar, 200 μm.

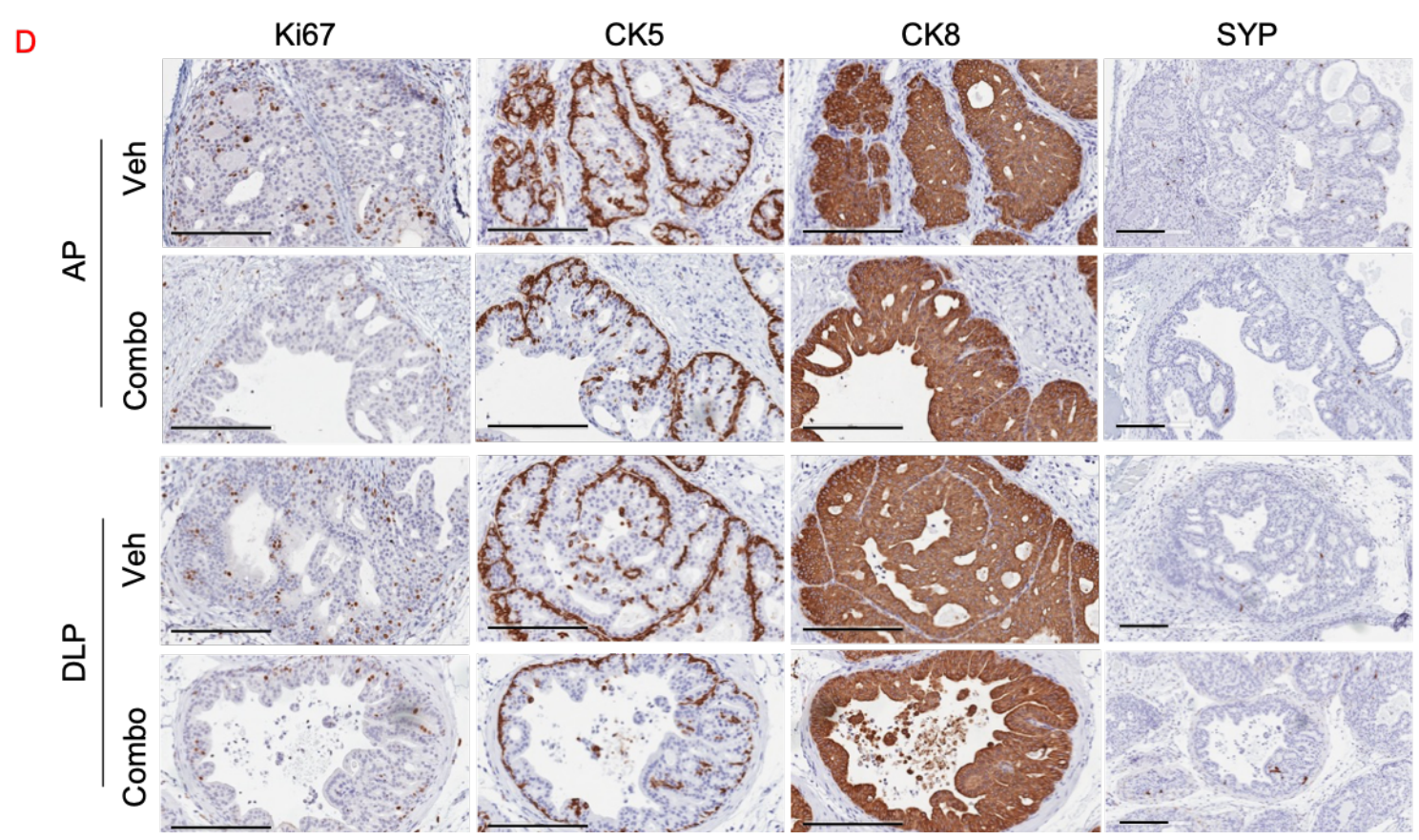

Figure S8D, Representative IHC staining of indicated markers in PSpC prostate tumors after vehicle or combination treatment. AP: anterior prostate; DLP: dorsolateral prostate. Scale bars, 200 μm.

Minor Points

1. Figures are generally clear, but some would benefit from more detailed legends (e.g., organoid dose–response curves, ChIP assays).

Response: The figure legends and Methods have been revised to include sufficient details of the experimental design.

2. Statistical methods are appropriate, but additional detail on sample size justification and blinding would improve rigor.

Response: Sample sizes and number of biological replicates have been indicated in the figure legends throughout the revised manuscript. Sample size justification and blinding were discussed in "**Statistics and Reproducibility" (Line 749).**

3. In figures illustrating patient data correlations (e.g., TCGA scatter plots), the number of samples analyzed should be clearly indicated in the legends.

Response: The number of samples analyzed has been clearly indicated in the revised figures and legends of Fig S5D and S7E.

REVIEWER COMMENTS

Reviewer #1 (Remarks to the Author):

Response: We thank this reviewer for the additional comments that help further improve the manuscript. All the comments have been addressed in the revised manuscript. Please find our point-by-point responses below.

Minor point:

1. IC50: Please report IC50 values in Figure 1C, E, F, G, Figure 2I, J, Figure 3G, Figure 4A, C and Figure 5F as well as in the relevant supplementary figures.

Response: Due to space limitations in the figures, IC50 values have been added to the source data file.

2. Figure 4E: Based on the figure label, it appears the comparison is CHD1 KO versus WT cells; however, the text suggests the intended comparison is CHD1 KO in a SPOP-mutant background versus SPOP-mutant cells. Please clarify the experimental context and labeling.

Response: The conclusion sentences have been revised to "*In contrast to what was observed following SPOP mutation (Fig. 2A), lipidomic profiling analysis revealed that CHD1 knockout led to a remarkable reduction of PUFA-containing phospholipids (Fig. 4E). Together, these results suggest that CHD1 plays a vital role in PUFA lipid metabolism, and CHD1 loss confers ferroptosis resistance in SPOP-mutated PCa.*" [Line 225]

3. Line 253 / Line 336: "CHD1-binding peaks" / "SP1-binding peaks" should be revised to "CHD1-binding sites" / "SP1-binding sites", respectively.

Response: These sentences have been revised as suggested [Line 250 and Line 333].

4. Line 273: The manuscript states that "loss of CHD1 abrogated c-Myc binding to target genes…," but the data presented demonstrate reduced MYC binding specifically at the ACSL4 promoter. Please revise the sentence to reflect this, unless additional evidence is provided to support a broader effect on multiple MYC target genes.

Response: This sentence has been revised to "loss of CHD1 abrogated c-Myc binding to ACSL4,…" [Line 270]

5. Figure S8E: This new panel should be introduced in the Results section (prior to its mention in the Discussion on line 430).

Response: The original Figure S8E has been moved to Figure S7I. A sentence has been added to introduce this result: "*Of note, RSL3 also showed a synergistic effect with the AR inhibitor Enzalutamide in SPOP-mutated PCa cells (Fig. S7I), suggesting the therapeutic potential of co-targeting ferroptosis and AR signaling in this context.*" [Line 349]

6. Endometrial carcinomas (EC; line 460): The basis for the statement that EC-associated SPOP mutations (M117V, D140G, E47K, and E50K) do not affect ACSL4 induction or ferroptosis sensitivity is unclear. If these data are included (e.g., in Rebuttal Figure 1), they should be incorporated as a supplementary figure and appropriately referenced in the manuscript; otherwise, the statement should be removed from the Discussion.

Response: The sentence "*Although 5-10% of endometrial carcinomas (EC) harbor SPOP mutations57, we didn't observe EC-associated SPOP mutations (M117V, D140G, E47K, and E50K) affecting ACSL4 induction or ferroptosis sensitivity in EC cells, suggesting that their effects and mechanisms of action may be context-dependent*" has been removed from the Discussion.

7. ChIP-seq tracks: Please include the MACS-called peak regions (peak calls) beneath the corresponding genome browser tracks to facilitate interpretation.

Response: The MACS-called peak regions (peak calls) have been added beneath the corresponding genome browser tracks in Figures S5G and S7F.

Reviewer #2 (Remarks to the Author):

The authors have adequately addressed all raised questions, the results are noteworthy and significant to the field of ferroptosis and related areas, and I therefore support publication in Nature Communications.

Response: We thank this reviewer for recognizing our work and for helping make this study more robust and significant.

Reviewer #3 (Remarks to the Author):

The authors have addressed most of my concerns and comments, and the revised manuscript has been significantly improved. I appreciate the effort the authors have put into revising the manuscript and responding to the reviewers' suggestions. Overall, the study is now much clearer and stronger.

Response: We thank this reviewer for recognizing our work and for helping make this study more robust and significant.
